# Join the Green and Sustainable Team: Magnesium Oxide Nanoparticles Boost Broad‐Spectrum Viral Resistance in Solanaceae Plants

**DOI:** 10.1111/pbi.70461

**Published:** 2025-11-14

**Authors:** Xiao‐Wen Wang, Li Ling, Ke‐Zheng Zhao, Jiangqi Wen, Zhaolin Ji, Xiao‐Ren Chen, Feng Zhu

**Affiliations:** ^1^ College of Plant Protection Yangzhou University Yangzhou China; ^2^ Department of Plant and Soil Sciences Oklahoma State University Stillwater Oklahoma USA

**Keywords:** magnesium oxide nanoparticles, phytohormones, plant immunity, reactive oxygen species (ROS), sustainable control, viral diseases

## Abstract

Plant viruses are so harmful to crops. It is an urgent need to develop modern, environment‐friendly, and sustainable plant viral epidemic‐management strategies that are safe for both human health and the environment. The field of nanotechnology is gaining increased interest in plant science. Magnesium oxide nanoparticles (MgONPs) have typical physical and chemical characteristics of nanomaterials. Hence, in this study, we systematically investigated the molecular mechanism of MgONPs triggering the plant immunity against viral pathogens. Foliar treatments allow MgONPs to enter *Nicotiana benthamiana* leaves through stomata and distribute within the intracellular space around chloroplasts through penetrating cell walls. MgONPs elevate plant growth and trigger dose‐dependent plant immunity against viral pathogens. Application of MgONPs triggers glutamate‐like receptors‐dependent Ca^2+^ flux and Ca^2+^ sensors. Exogenous application of MgONPs does not trigger resistance in Ca^2+^ channel‐blocked plants, and knockout of *NbGLR3.3* weakens the resistance induced by MgONPs. MgONPs induce early ROS bursts but reduce oxidative damage and accumulation of ROS after TMV infection at late stages. MgONPs activate Ca^2+^‐dependent SA‐, JA‐, and ET‐mediated signalling pathways, and the absence of SA‐, JA‐, or ET signals weakens the MgONPs‐triggered resistance. However, MgONPs fail to induce resistance to viral pathogens in plants simultaneously lacking SA, JA, and ET. Safety evaluation showed that MgONPs have desirable biocompatibility and biosafety for plants, as well as satisfactory biosafety for the aquatic environment. Overall, our discoveries point to a new direction for MgONPs as effective, non‐drug‐resistant, non‐toxic, sustainable, residual‐free, and eco‐friendly antiviral agents to simultaneously prevent diverse viral diseases.

## 1 Introduction

Plants are often attacked by various phytopathogens during their growth and development, such as bacteria, fungi, oomycetes, viruses, and so on. These phytopathogens cause serious plant diseases and cause an estimated crop loss of up to 20%–30% annually (Kashyap et al. 2017). Plant viruses are so harmful to crops that they are called “plant cancers”. At present, more than 2100 species of plant viruses have been identified by the ICTV (International Committee on Taxonomy of Viruses) (Walker et al. 2021). Plant viruses are a major cause of plant diseases, causing an estimated economic loss of more than $30 billion annually in global agricultural production (Scholthof et al. 2011; Jones and Naidu 2019). A list of the top 10 plant viruses was generated by plant virologists on the basis of their scientific or economic importance, and tobacco mosaic virus (TMV) was ranked atop the list (Scholthof et al. 2011). TMV belongs to the Tobamovirus genus, which can infect more than 350 species in more than 30 families, such as Solanaceae and Cucurbitaceae. TMV is easily transmitted and widely distributed all over the world. It can cause severe crop diseases and lead to great economic losses of up to $2.5 billion annually worldwide (Jiang et al. 2024). Unfortunately, there are no currently available, internationally recognised high‐efficiency anti‐TMV agrochemicals or effective strategies to control TMV. So far, although several methods have been used to control viral diseases, such as chemical and biological controls and resistance breeding, viral disease control is still a difficult task in plant disease management (Liu et al. 2024). In particular, the extensive use of chemical pesticides led to many environmental and social problems, such as threats to human health, agrochemical residues in food, disruption of agricultural biodiversity and ecosystem balance, and the development of resistant strains of pests and pathogens (Zhu et al. 2024). Hence, in order to achieve agricultural sustainability and food security, there is an urgent need to develop modern, environment‐friendly, and lasting plant viral epidemic‐management strategies that are safe for both human health and the environment.

Recently, significant technological advancements and innovations have been made in the field of agriculture (Xiao et al. 2013; Dwivedi et al. 2016; Kou et al. 2018). For example, activation of plant immunity by the application of plant immunity inducers has become popular as a promising eco‐friendly and sustainable strategy for future plant disease control (Zhu et al. 2024). It is well known that plants have evolved sophisticated multiple layers of defence mechanisms to restrict and prevent pathogen infections. PAMP‐triggered immunity (PTI), induced systemic resistance (ISR), effector‐triggered immunity (ETI), systemic acquired resistance (SAR), and RNA silencing (RNAi) are the primary modes of plant immunity (Chang et al. 2022; Zhu et al. 2024). The field of nanotechnology is gaining increased interest in plant science, especially for the application of nanomaterials (NMs) as vehicles of agrochemicals or biomolecules in plants, and the great potential to suppress pathogen infection, enhance crop productivity, and improve plant growth (Khan et al. 2017; Hao et al. 2018). NMs, as nanofertilizers, can efficiently deliver micronutrients to plants and trigger plant immunity for crop protection (Servin et al. 2015; Wang, Cunningham, et al. 2021; Wang, Gong, et al. 2021). For example, exogenous application of silica nanoparticles (SiO_2_NPs) in Arabidopsis and rice induces local and systemic resistance against the bacterial pathogen *Pseudomonas syringae* and the fungal pathogen *Magnaporthe oryza*, respectively (El‐Shetehy et al. 2021; Du et al. 2022). Application of copper oxide nanoparticles (CuONPs) on *Nicotiana benthamiana* not only directly breaks viral particles of TMV, but also triggers plant immunity by activating SAR against TMV infection (Liu et al. 2024). Nanomaterials have attracted special attention as new nano‐agricultural chemicals for the prevention and control of plant diseases because of their excellent physical and chemical properties (Elmer and White 2018; Zhao et al. 2022). Among them, magnesium oxide nanoparticles (MgONPs) are a new type of magnesium oxide with a particle size of about 50 nm. They have typical physical and chemical characteristics of nanomaterials. They are non‐toxic and easy to prepare from readily available and economical precursors (Imada et al. 2016). In addition, MgONPs are easy to be adsorbed by soil, improve fertiliser utilisation, and can be used as an element supplement to increase magnesium content in soil. They have been recognised as safe materials by the U.S. Food and Drug Administration (Kuo et al. 2023). The study showed that MgONPs promote plant growth (Gautam et al. 2023). In addition, MgONPs can not only directly inhibit the growth of pathogens but also induce plant resistance against pathogen infections (Salas‐Leiva et al. 2021). Application of MgONPs in the roots of tomato plants triggers systemic resistance against bacterial wilt disease caused by *Ralstonia solanacearum* (Imada et al. 2016). However, the molecular mechanism of how MgONPs induce plant immunity to enhance plant resistance to virus infection is still unknown.

Hence, in this study, we systematically investigated the molecular mechanism of MgONPs triggering plant immunity against viral pathogens. We found that MgONPs enhanced host resistance against TMV infection through activating Ca^2+^ flux via plant glutamate‐like receptors (GLRs), Ca^2+^‐dependent phytohormones and early ROS bursts, reducing oxidative damage and the accumulation of ROS after TMV infection at late stages, and improving plant growth and productivity. Toxicity assessment assays in plants and animals demonstrated that MgONPs possessed desirable biocompatibility and biosafety in plants and satisfactory biosafety in the aquatic environment. In particular, exogenous application of MgONPs triggered broad‐spectrum resistance against major viral diseases of vegetable crops in Solanaceae plants. Therefore, our breakthrough discoveries pave the way for nanomaterials to become efficient, non‐toxic, residual‐free, sustainable, non‐drug‐resistant, and environmentally friendly antiviral agents to resist viral diseases in agricultural production.

## 2 Results

### 2.1 Characterisation of MgONPs

Scanning/transmission electron microscopy (SEM/TEM) images (Figure S1a,b) showed the morphology of MgONPs. Most of the MgONP particles are spherical and exist in aggregate form, which is also consistent with the results of previous studies. The reason for this aggregation phenomenon may be the strong interaction between the particles of MgO at the nanoscale (Shahzadi et al. 2022). SEM images further confirmed that the size range of MgONPs particles is 46.76 ± 7.35 nm (Figure S1c). The energy‐dispersive X‐ray spectroscopy (EDS) spectrum and elemental mapping indicated that Mg (Weight 53.28%; Atom 42.88%) and oxygen (O; Weight 46.72%; Atom 57.12%) are the major elements of MgONPs (Figure S1d,e). Fourier transform infrared spectroscopy (FTIR) analysis confirmed the presence of various functional groups in MgONPs that provide insights into the chemical composition of MgONPs and have the effect of maintaining long‐term stability. According to the FTIR image, —OH in Mg(OH)_2_ stretched vibrations in the range of 3697 cm^−1^ and C—O stretched vibrations in the range of 1105 cm^−1^, indicating the presence of Mg(OH)_2_ and C—O. Similarly, the peak at 625 cm^−1^ was attributed to the tensile vibration of the Mg‐O bond, indicating the presence of MgONPs (Figure S1f) (Noman et al. 2024). Crystal characteristics of NPs are of great significance as they provide valuable insights into the physical state, release kinetics, and interaction capacity of NPs with the encapsulating substrate (Zahir et al. 2019). The crystalline features of MgONPs were obtained through X‐ray diffraction (XRD) spectra. Different spectral peaks were observed at 74.6° (311), 78.6° (222), 62.3° (220), 42.9° (200), and 36.9° (111) at 2θ degrees, which were indexed to the crystal structure and crystallographic facets of MgO (Ahmed et al. 2021). The XRD results indicated high quality and homogeneity of MgONPs. In addition, the presence of diffraction peaks corresponding to some Mg (OH)_2_ indicates that moisture in the air causes some MgONPs to absorb moisture in the form of Mg (OH)_2_ (Figure S1g).

### 2.2 Screening Concentration and MgONPs Trigger Dose Dependence of Plant Immunity

To investigate the effect of MgONPs in plant immunity against virus infection, *N. benthamiana* leaves were sprayed with water or various concentrations of MgONPs (0, 50, 100, 150, 200, and 250 μg/mL) and then inoculated with green fluorescent protein (GFP)‐tagged TMV, a recombinant TMV that is infectious. There was no obvious difference in the phenotype between various concentrations of MgONP treatments and the control (Figure S2). The results showed that there was a reduction in the number of GFP fluorescent foci in inoculated leaves of various concentrations of MgONP treatment by spraying, as compared to water‐treated plants over a time course of 7 days after TMV‐GFP infection (Figure S3). Among several concentrations, 150 μg/mL of MgONP treatment showed the best antiviral effect since it reduced the number of GFP fluorescent foci of TMV most significantly (Figure S3a,b). These results suggest that exogenous application of MgONPs induces resistance to TMV‐GFP infection. Using a standard log‐logistic dose–response model, we found a dose‐dependent manner between the antiviral effect and MgONP concentration under the dynamic range of 150 μg/mL (Figure S3c), indicating that MgONPs trigger dose dependence of plant immunity to viral pathogens. 150 μg/mL MgONPs was used in all subsequent experiments. Therefore, our results indicated that MgONP‐induced resistance against TMV was functional in a suitable range of relatively low concentrations and MgONPs have the potential for effective viral disease control.

### 2.3 Exogenous Application of MgONPs Enhances Plant Resistance Against TMV Infection

**FIGURE 1:**
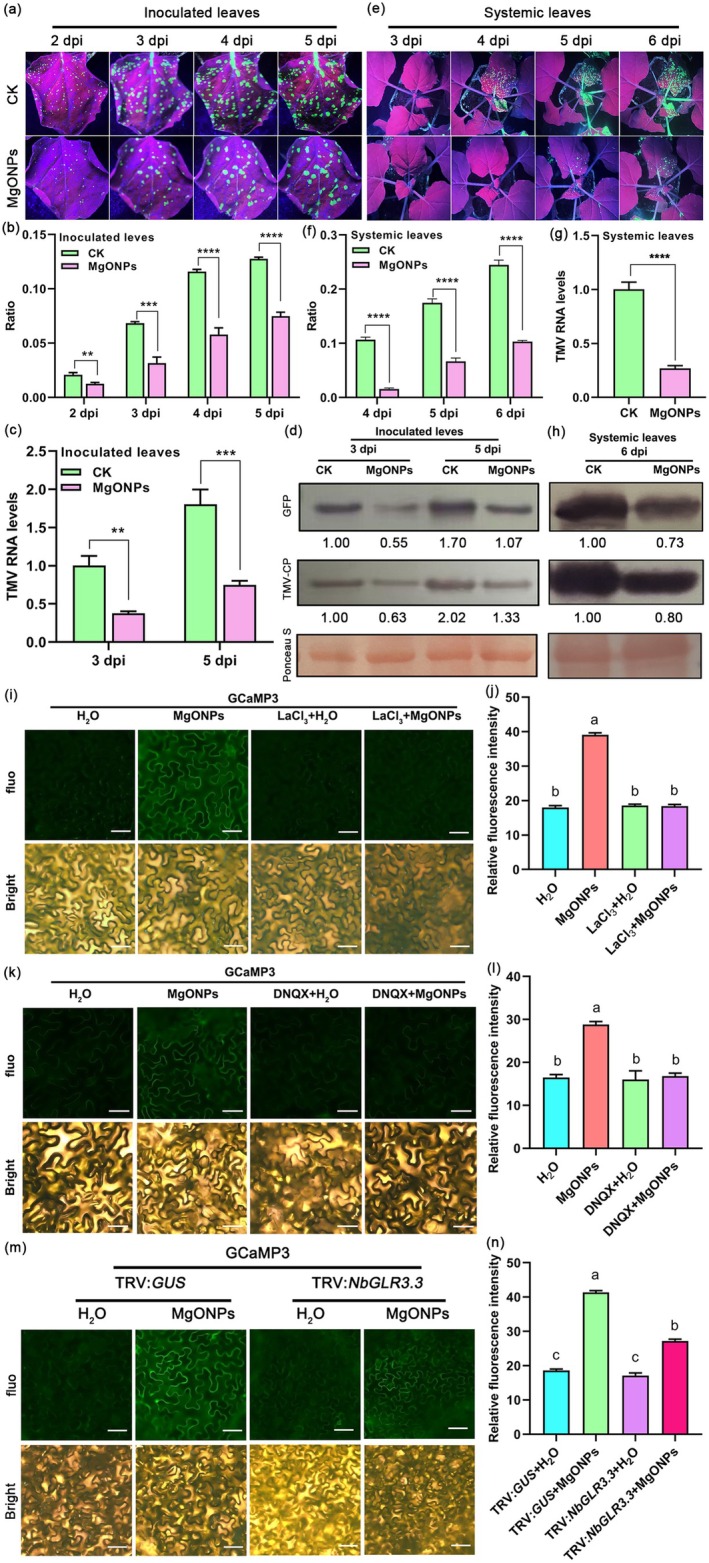
Exogenous application of MgONPs increases resistance to TMV‐GFP and triggers glutamate‐like receptor‐dependent Ca^2+^ influx. (a) Analysis of GFP fluorescence in the inoculated leaves of MgONP‐treated plants and water‐treated plants (CK) at different time points after infection with TMV‐GFP. (b) Ratio of infected area to the total area of the inoculated leaves of MgONP‐treated plants and water‐treated plants (CK) shown in (a). (c) TMV RNA levels in the inoculated leaves of MgONPs‐treated plants and water‐treated plants (CK) at 3 and 5 dpi with TMV‐GFP determined by RT‐qPCR. (d) Western blotting of the accumulation of TMV coat protein in the inoculated leaves of MgONP‐treated plants and water‐treated plants (CK) at 3 and 5 dpi after TMV‐GFP infection. Rubisco proteins were used as loading controls and were stained by Ponceau S. (e) Representative images of GFP fluorescence visualised in uninoculated upper leaves (systemic leaves) of MgONP‐treated plants and water‐treated plants (CK) at different time points after infection with TMV‐GFP. (f) The ratio of the GFP fluorescent area to the total area of the systemic leaves shown in (e) at 4–6 dpi. (g) RT‐qPCR analysis of TMV replication levels in the systemic leaves of MgONPs‐treated and water‐treated plants (CK) at 6 dpi after TMV‐GFP infection. (h) Western blotting of the accumulation of TMV coat proteins in the systemic leaves shown in (e) at 6 dpi. Rubisco proteins were used as loading controls and were stained by Ponceau S. (i) Fluorescence images of Ca^2+^ signaling in leaves of *GCaMP3*‐overexpressing transgenic *N. benthamiana* under different treatments. The *GCaMP3*‐transgenic *N. benthamiana* plants were treated with ddH_2_O, 150 μg/mL MgONPs, 5 mM LaCl_3_ (Ca^2+^ channel blocker) or 5 mM LaCl_3_ + 150 μg/mL MgONPs. Scale bar, 50 μm. (j) Relative fluorescence intensity of Ca^2+^ signals in the leaves shown in (i). (k) Fluorescence images of Ca^2+^ signaling in the leaves of *GCaMP3*‐transgenic *N. benthamiana* plants under different treatments. The *GCaMP3*‐transgenic plants were treated with ddH_2_O, 150 μg/mL MgONPs, 0.5 mM DNQX (glutamate receptor inhibitor) or 0.5 mM DNQX +150 μg/mL MgONPs. Scale bar, 50 μm. (l) Relative fluorescence intensity of Ca^2+^ signals of the leaves shown in (k). (m) Fluorescence images of Ca^2+^ signaling in the leaves of *NbGLR3.3*‐silenced plants in *GCaMP3*‐overexpressing *N. benthamiana* background under 150 μg/mL MgONPs or ddH_2_O treatments. Scale bar, 50 μm. (n) Relative fluorescence intensity of Ca^2+^ signals of the leaves shown in (m). Asterisks represent significant differences determined by Student's *t*‐test between two groups (**p < 0.01; ***p < 0.001; ****p < 0.0001). Different letters indicate significant differences as determined using one‐way ANOVA followed by Tukey's test between multiple groups (*p* < 0.05).

No obvious difference in the phenotype was observed between MgONP‐treated and control plants (Figure S4). Our results showed that there was a marked reduction in the number of GFP fluorescent foci in inoculated leaves of 150 μg/mL MgONP treatment by spraying, as compared to water‐treated plants at different time points after TMV‐GFP infection (Figure 1a,b). RT‐qPCR results revealed that TMV RNA levels were reduced in inoculated leaves of MgONP‐treated plants as compared to control plants (Figure 1c). Furthermore, western blotting indicated that the coat protein levels of TMV (TMV‐CP) and GFP protein levels were also reduced in inoculated leaves of MgONP treatment in comparison with the control (Figure 1d). We further monitored the TMV replication and spread in the systemic upper leaves for 6 days. Results from GFP fluorescence imaging (Figure 1e), GFP fluorescent foci quantification (Figure 1f), RT‐qPCR (Figure 1g), and western blotting (Figure 1h) confirmed that reduced viral accumulation was observed in upper systemic leaves of 150 μg/mL MgONP‐treated plants compared with the control.

### 2.4 MgONPs Subcellular Distribution Within the Leaves

The interaction of MgONPs with *N. benthamiana* plants was assessed by TEM 3 days after the application of MgONPs (Figure S5). Our previous experiments showed that at this time point, the MgONP‐exposed *N. benthamiana* plants had already developed viral resistance (Figure 1a–h). Results showed that the size range of ~40–50 nm of MgONPs under foliar treatment might allow them to enter *N. benthamiana* leaves through stomata and distribute within the intracellular space through penetrating cell walls. After further observation, TEM results indicated that the aggregation of nanoparticles around chloroplasts in leaves was observed (Figure S5). At the same time, chloroplasts maintained normal subcellular structure and morphology (Figure S5). Therefore, TEM observations of *N. benthamiana* leaves under MgONP treatments confirmed that MgONPs did not damage *N. benthamiana* leaf tissues and cell morphology, implying no toxic side effects of MgONPs on plant cells. These findings provided basic information for further evaluation of the molecular mechanism of MgONPs triggering plant immunity against viral pathogens in *N. benthamiana* plants.

### 2.5 Application of MgONPs Triggers Glutamate‐Like Receptors‐Dependent Ca^2+^ Flux and Ca^2+^ Sensors

Ca^2+^ signals play an important role in plant immunity (Köster et al. 2022). Plant glutamate‐like receptors (GLRs) have been associated with Ca^2+^ signalling by directly channelling its extracellular influx into the cytosol (Wudick et al. 2018). However, whether exogenous application of MgONPs can trigger Ca^2+^ flux to activate plant immunity against viral diseases is unknown. We examined Ca^2+^ signals in response to exogenous application of MgONPs in leaf tissues of *GCaMP3*‐overexpressing transgenic *N. benthamiana* (DeFalco et al. 2017) using fluorescence microscopy. A strong fluorescence signal was observed in MgONPs‐treated plants compared to those pretreated with water only (Figure 1i,k). However, when *N. benthamiana* plants were pretreated with LaCl_3_ (a Ca^2+^ channel blocker) or DNQX (6,7‐dinitroquinoxaline‐2,3‐dione, a glutamate receptor inhibitor), MgONP‐triggered fluorescence signals were markedly weakened (Figure 1i,k). GFP fluorescence quantification also confirmed that exogenous application of MgONP triggers Ca^2+^ flux (Figure 1j,l). GLR3.3 plays an important role in Ca^2+^ signalling (Stephens et al. 2007; Grenzi et al. 2022); hence, we employed VIGS on the basis of tobacco rattle virus (TRV) to suppress the expression of *NbGLR3.3* in *GCaMP3*‐overexpressing transgenic *N. benthamiana*. No obvious phenotypic difference was observed between the *NbGLR3.3*‐silenced and the control (TRV:*GUS*) plants (Figure S6a). RT‐qPCR was employed to examine the gene‐silencing efficiency of *NbGLR3.3* at 12 d post‐infiltration with the TRV constructs. Results indicated that the transcript levels of *NbGLR3.3* were significantly reduced in *NbGLR3.3*‐silenced plants compared to the control (Figure S6b). A strong fluorescence signal emerged in MgONPs‐treated leaves of control plants (TRV:*GUS*). However, the MgONP‐triggered fluorescence signal was markedly decreased in MgONP‐treated leaves of *NbGLR3.3*‐silenced plants (Figure 1m). GFP fluorescence quantification results further confirmed that silencing of *NbGLR3.3* attenuated the MgONP‐triggered Ca^2+^ flux (Figure 1n).

Next, we detected the expression of all *NbGLR* genes in MgONP‐treated plants in a 24 h time course by PCR analysis (Figure S7). RT‐qPCR results showed that the expressions of almost all *NbGLR* genes were significantly increased in the leaves of MgONPs early treatment, indicating that the exogenous application of MgONPs activates the expression of *GLRs* genes (Figure S7). Cytoplasmic Ca^2+^ concentration changes are perceived by Ca^2+^ sensor proteins (such as calmodulin, CaM, and Ca^2+^‐dependent protein kinase, CDPK) and activate downstream signal transduction pathways (Ca^2+^ signalling) (Jiang and Ding 2023). Previous studies suggest that Arabidopsis *AtRBOHD*‐dependent ROS production is of great importance to biotic stresses and AtCDPK5 phosphorylates AtRBOHD to regulate *AtRBOHD*‐dependent ROS generation (Dubiella et al. 2013; Liu and He 2016). The amino acid sequences of NbCDPK4, NbCDPK5, and NbCDPK6 from *N. benthamiana* show high homology with AtCDPK5 (data not shown). Thus, we examined the expression of *NbCaM1‐6* and *NbCDPK4‐6* genes in MgONPs‐treated plants in a 24 h time course (Figure S8). RT‐qPCR results indicated that the transcript levels of *NbCaM1*, *NbCaM2*, *NbCaM3*, *NbCaM4*, *NbCaM5*, *NbCaM6*, *NbCDPK4*, *NbCDPK5*, and *NbCDPK6* were all significantly increased in MgONP‐treated plants compared with water‐treated plants at different time points (Figure S8).

### 2.6 Exogenous Application of MgONPs Does Not Trigger Resistance in Ca^2+^ Channel‐Blocked Plants

**FIGURE 2:**
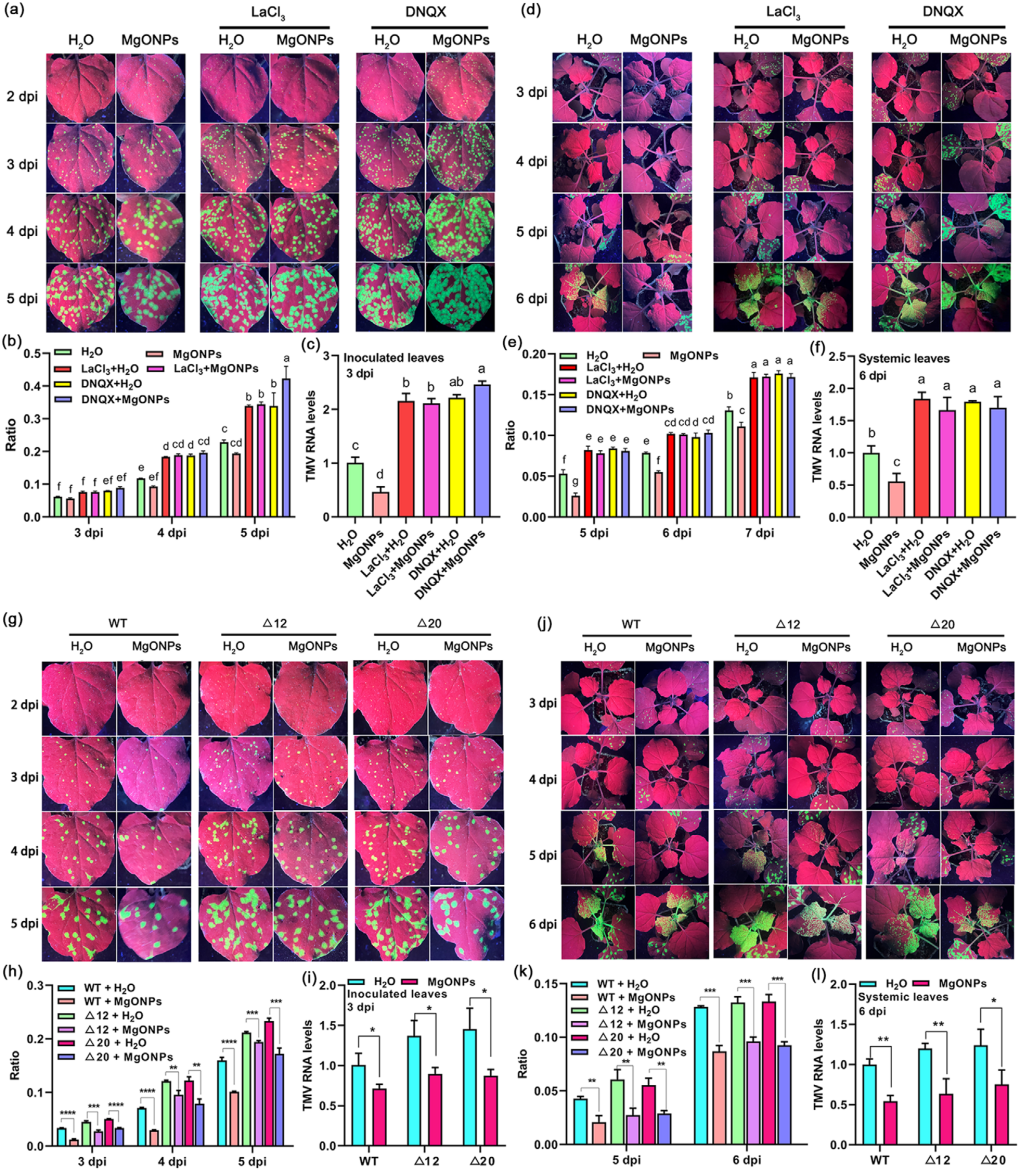
Exogenous application of MgONPs does not trigger resistance in Ca^2+^ channel‐blocked plants, and knockout of *NbGLR3.3* weakens the resistance induced by MgONPs. (a) Representative images of GFP fluorescence visualised in the inoculated leaves of Ca^2+^ channel‐blocked plants under MgONPs and water treatments at different time points after infection with TMV‐GFP. *N. benthamiana* plants pretreated with LaCl_3_ or DNQX were sprayed with MgONPs, then infected by TMV‐GFP. (b) The ratio of the GFP fluorescent area to the total area of the inoculated leaves shown at 3–5 dpi. (c) TMV RNA levels in the inoculated leaves of Ca^2+^ channel‐blocked plants under MgONP treatments at 3 dpi with TMV‐GFP, as determined by RT‐qPCR. *Actin* was used as the internal reference, and expression is relative to that in the WT with ddH_2_O treatment at 3 dpi, the value of which was set as 1. (d) Analysis of GFP fluorescence in the systemic leaves of Ca^2+^ channel‐blocked plants under MgONPs treatments at different time points after infection with TMV‐GFP. (e) The ratio of the GFP fluorescent area to the total area of the systemic leaves shown in (d) at 5–7 dpi. (f) TMV RNA levels in the systemic leaves of Ca^2+^ channel‐blocked plants under MgONPs treatments at different time points after infection with TMV‐GFP, as determined by RT‐qPCR. (g) Representative images of GFP fluorescence visualised in the inoculated leaves of *Nbglr3.3* mutant plants under MgONPs and water treatments at different time points after infection with TMV‐GFP. The *Nbglr3.3* mutants (∆12 and ∆20) were sprayed with water or 150 μg/mL MgONPs, and then inoculated with TMV‐GFP. (h) The ratio of the GFP fluorescent area to the total area of the inoculated leaves shown in (g) at 3–5 dpi. (i) RT‐qPCR analysis of the TMV RNA levels in the inoculated leaves of *Nbglr3.3* mutant plants under MgONPs and water treatments at 3 dpi with TMV‐GFP. *Actin* was used as the internal reference gene, and the expression is relative to that in the WT with ddH_2_O treatment at 3 dpi, the value of which was set as 1. (j) Analysis of GFP fluorescence in the systemic leaves of *Nbglr3.3* mutant plants under MgONPs and water treatments at different time points after infection with TMV‐GFP. (k) The ratio of the GFP fluorescent area to the total area of the systemic leaves shown in (j) at 5 dpi and 6 dpi. (l) TMV RNA levels in the systemic leaves of *Nbglr3.3* mutant plants under MgONPs and water treatments at 6 dpi after infection with TMV‐GFP, as determined by RT‐qPCR. *Actin* was used as the internal reference gene, and the expression is relative to that in the WT with ddH_2_O treatment at 6 dpi, the value of which was set as 1. Different letters indicate significant differences as determined using one‐way ANOVA followed by Tukey's test between multiple groups (*p* < 0.05). Asterisks represent significant differences determined by Student's *t*‐test between two groups (*p < 0.05; **p < 0.01; ***p < 0.001; ****p < 0.0001).

In order to investigate whether MgONPs can induce viral resistance in Ca^2+^ channel‐blocked plants, *N. benthamiana* plants pretreated with LaCl_3_ or DNQX were sprayed with MgONPs, then infected with TMV‐GFP. No obvious phenotypic difference was observed between the MgONPs, LaCl_3_ + H_2_O, LaCl_3_ + MgONPs, DNQX+H_2_O, DNQX+MgONP‐treated plants and the water‐treated plants (Figure S9). There was a significant increase in the number of GFP fluorescent foci in inoculated leaves and systemic leaves of the plants treated with LaCl_3_ or DNQX compared to the water‐treated plants in a 5–6‐day time course after TMV‐GFP infection (Figure 2a,b,d,e). RT‐qPCR results also showed that TMV RNA levels in the inoculated leaves and systemic leaves of the LaCl_3_ or DNQX‐treated plants were also enhanced at these time points compared to the control plants (Figure 2c,f), indicating that blocking of Ca^2+^ channels compromises plant local and systemic resistance to TMV. MgONPs markedly reduced the TMV accumulation in water‐treated plants (Figure 2a–f), however, the results of GFP fluorescence imaging (Figure 2a,d), GFP fluorescent quantification (Figure 2b,e), and RT‐qPCR (Figure 2c,f) at 5–6 days all confirmed that there were no significant differences in the viral accumulation in the inoculated leaves and systemic leaves of LaCl_3_ or DNQX‐treated plants pretreated with MgONPs compared to those pretreated with water only (Figure 2a–f). Overall, these results confirmed that the exogenous application of MgONPs does not trigger viral resistance in Ca^2+^ channel‐blocked plants.

### 2.7 Knocking Out *NbGLR3.3* Weakens the Resistance Induced by MgONPs

In order to further confirm that *NbGLR3.3* is required for MgONP‐induced plant immunity, we employed gene editing technology on the basis of the CRISPR/Cas9 system to knock out the expression of *NbGLR3.3* in *N. benthamiana*. Two target sites, Target 1 and Target 2, were designed for *NbGLR3.3* (Figure S10a). The PCR products were cloned into the pKSE401 vector by homologous recombination technology (Figure S10a). The recombinant plasmid was confirmed by plasmid PCR (Figure S10b), and 29 independent transgenic plants were obtained through *Agrobacterium*‐mediated transformation. PCR was performed to detect the expression levels of the *NptII* gene, indicating that the expression of the *NptII* gene was observed in all lines except lines 4, 18, and 29 (Figure S10c). PCR results confirmed that the target gene was edited in 1, 2, 3, 12, 13, 14, 17, 19, 20, 22 and 23 lines (Figure S10d,e). To further verify the editing types in *Nbglr3.3* mutants, these independent transgenic lines were analysed by sequencing. We found that there was a 1‐base insertion at Target site 1 and a 3‐base deletion at Target site 2 in the ∆12 line, and a 2‐base insertion at Target site 1 and an 8‐base deletion at Target site 2 in the ∆20 line (Figure S10f). Hence, we successfully generated *Nbglr3.3* mutants using the CRISPR/Cas9 gene editing technology. Next, ∆12 and ∆20 lines were selected to further study the role of MgONPs in plant immunity.

The *Nbglr3.3* mutants (∆12 and ∆20) were sprayed with water or 150 μg/mL MgONPs, and then inoculated with TMV‐GFP. No obvious phenotypic difference was observed between MgONPs‐treated *Nbglr3.3* mutants and control plants (Figure S11). There was a significant increase in the number of GFP fluorescent foci in the inoculated leaves and systemic leaves of the *Nbglr3.3* mutants (∆12 and ∆20) compared to WT plants with water treatment in a 5–6‐day time course after TMV‐GFP infection (Figure 2g,h,j,k). RT‐qPCR results suggest that TMV RNA levels in the inoculated leaves and systemic leaves of the *Nbglr3.3* mutants (∆12 and ∆20) were also enhanced at these time points compared to WT plants (Figure 2i,l). Remarkably, the inoculated leaves and systemic leaves of WT plants treated with MgONPs showed less GFP fluorescence and fewer GFP fluorescent foci than water‐treated plants at various time points following TMV‐GFP infection (Figure 2g,h,j,k). There was also a reduction in the number of GFP fluorescent foci and TMV RNA levels in the inoculated leaves and systemic leaves of the *Nbglr3.3* mutants (∆12 and ∆20) with MgONP treatment compared to water‐treated *Nbglr3.3* mutants (Figure 2g–l). However, the extent of reduction in the number of GFP fluorescent foci and TMV RNA levels in *Nbglr3.3* mutants with MgONPs treatment was less than the reduction in the number of GFP fluorescent foci and TMV RNA levels in WT plants treated with MgONPs (Figure 2g–l). These results indicated that knocking out *NbGLR3.3* weakens the resistance induced by MgONPs.

### 2.8 MgONPs Induce Early ROS Bursts but Reduce Oxidative Damage and Accumulation of ROS After TMV Infection at Late Stages

**FIGURE 3:**
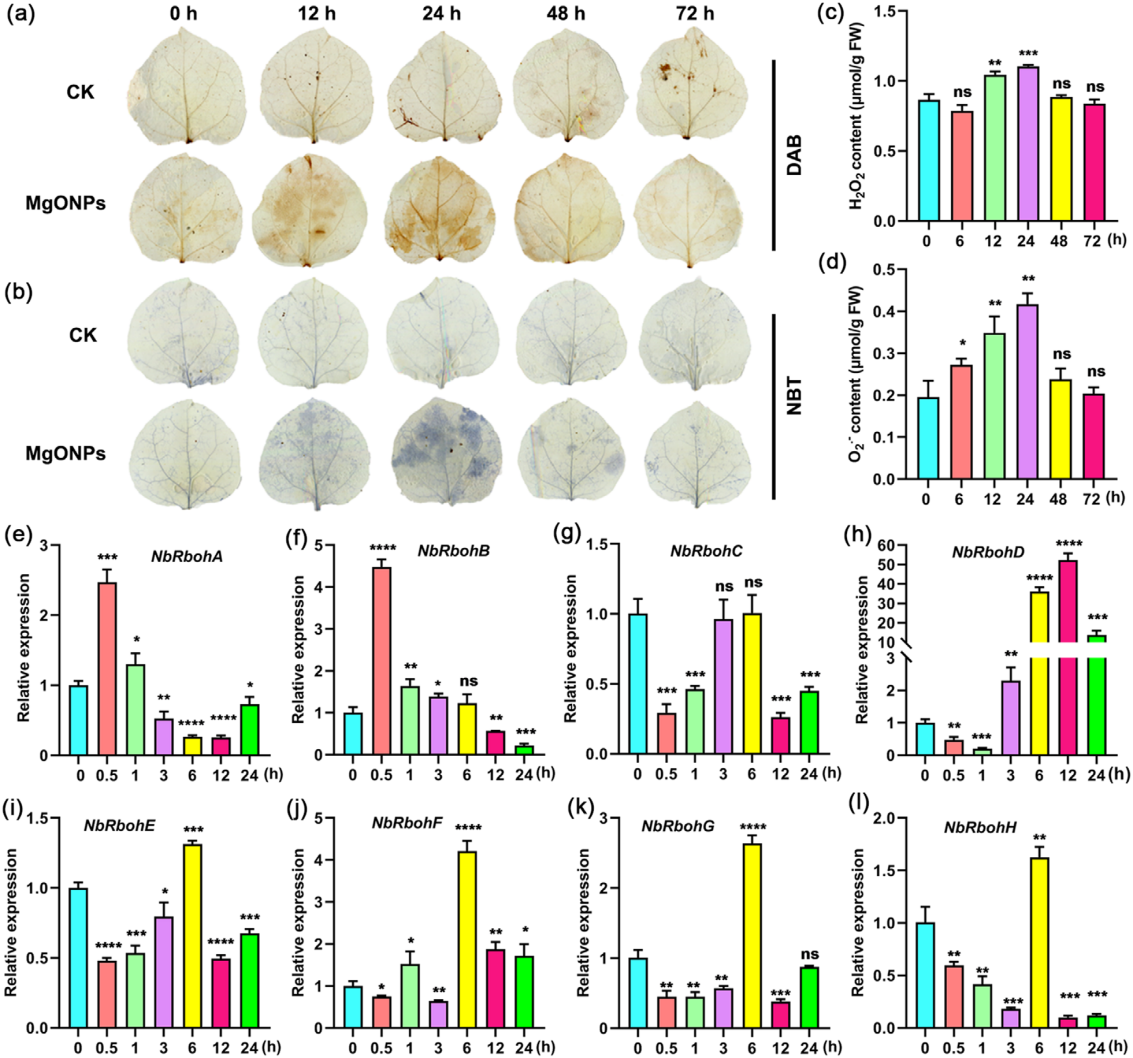
MgONP treatment induces early ROS bursts. (a, b) The levels of H_2_O_2_ and O_2_˙^−^ were visualized by DAB (a) and NBT (b) staining, respectively, of MgONPs (150 μg/mL) or water‐treated leaves at different time points. (c, d) The H_2_O_2_ (c) and O_2_˙^−^ (d) content were measured in MgONPs or water‐treated leaves at different time points by a Hydrogen Peroxide Assay Kit and a Superoxide Anion Activity Content Assay Kit, respectively. (e–l) Quantitative real‐time PCR analysis of the expression of eight *NbRbohs* genes in MgONPs (150 μg/mL)‐treated leaves at different time points. *Actin* was used as the internal reference gene, and the expression is relative to that in the WT with ddH_2_O treatment, the value of which was set as 1. Asterisks represent significant differences determined by Student's *t*‐test (*p < 0.05; **p < 0.01; ***p < 0.001; ****p < 0.0001).

The production of ROS is a key component of plant immune responses (Wu et al. 2023). An early ROS burst contributes to the resistance against plant viruses such as TMV (Zhu et al. 2023). Therefore, we first investigated the levels of ROS in MgONP‐treated plants in a 72‐h time course. The dark‐brown DAB polymer products from H_2_O_2_ and blue NBT polymer products from O_2_˙^−^ were significantly increased in MgONPs‐treated plants at 12 h and 24 h of treatment (Figure 3a,b). To determine the H_2_O_2_ and O_2_˙^−^ contents more precisely, we used a Hydrogen Peroxide Assay Kit to examine the H_2_O_2_ levels and a Superoxide Anion Activity Content Assay Kit to detect the O_2_˙^−^ levels in these samples. Consistent with ROS staining assays, our results showed that the levels of H_2_O_2_ and O_2_˙^−^ were significantly higher in MgONPs‐treated plants at 12 h and 24 h of treatment compared with water‐treated plants (Figure 3c,d). Plant NADPH oxidases, also known as respiratory burst oxidase homologues (RBOHs), are involved in active ROS production (Torres and Dangl 2005). Thus, we examined the expression patterns of eight *NbRbohs* in MgONPs‐treated plants in a 24‐h time course (Figure 3e–l). The expression of *NbRbohA* and *NbRbohB* was significantly increased in MgONPs‐treated plants at 0.5 h and 1 h of treatment (Figure 3e,f). The transcript levels of *NbRbohD* were significantly higher in the MgONP‐treated plants after 6 h, 12 h and 24 h treatment than those in water‐treated plants (Figure 3h). The expression of *NbRbohE‐H* was significantly induced at 6 h after MgONPs treatment (Figure 3i–l). Overall, these results indicated that exogenous application of MgONPs activates early ROS bursts.

However, the over‐accumulation of ROS at late stages of pathogen infection (e.g., at 2–3 dpi) can cause oxidative stress to plants and make them more vulnerable to various other pathogens (Zhu et al. 2021, 2023). Thus, we investigated the levels of H_2_O_2_ and O_2_˙^−^ in MgONP‐treated plants infected with TMV‐GFP using DAB and NBT staining, respectively. ROS staining assays showed that the levels of H_2_O_2_ and O_2_˙^−^ were significantly increased in both MgONP‐treated and water‐treated plants at 3 dpi with TMV‐GFP infection. However, the levels were noticeably lower in MgONP‐treated plants (Figure S12a). Direct measurements of H_2_O_2_ and O_2_˙^−^ showed that their contents were markedly reduced in MgONP‐treated plants compared with water‐treated plants at 3 dpi with TMV‐GFP (Figure S12b,c). Next, the levels of H_2_O_2_ were visualised with the H_2_O_2_ fluorescent probe 2′,7′‐dichlorofluorescein diacetate (H2DCF‐DA), using a fluorescence microscope (Figure S12d). At 3 dpi with TMV‐GFP infection, the levels of H_2_O_2_ were clearly enhanced in both MgONP‐treated and water‐treated plants. However, MgONP‐treated plants showed lower fluorescence intensity than control plants after TMV‐GFP infection (Figure S12d). Fluorescence quantification results further confirmed that the levels of H_2_O_2_ were markedly lower in MgONP‐treated plants than in control plants (Figure S12e). Oxidative damage induced by virus infection could adversely affect the cytomembranes. Increases in the MDA content and the electrolyte leakage are usually considered as indicators for membrane lipid peroxidation, cell death and penetrability of cytomembranes (Zhu et al. 2021). Therefore, we examined the MDA content and electrolyte leakage in MgONP‐treated plants infected with TMV‐GFP at 3 dpi. There was no significant difference in the electrolyte leakage and MDA content between MgONP‐treated plants and control plants without TMV‐GFP (Figure S12f,g). However, the level of electrolyte leakage was significantly reduced in MgONP‐treated plants compared with control plants at 3 days after TMV‐GFP inoculation, implying that the cytomembranes of MgONP‐treated plants suffered less oxidative damage under TMV‐GFP infection (Figure S12f). Consistent with the electrolyte leakage, the MDA contents were decreased in MgONP‐treated plants compared with control plants after TMV‐GFP infection, indicating that the exogenous application of MgONPs weakened peroxidation of membrane lipids under TMV‐GFP infection (Figure S12g). Taken together, these results indicated that the exogenous application of MgONPs reduces oxidative damage and the accumulation of ROS after TMV infection at late stages.

### 2.9 Foliar Application of MgONPs Activates the Activity and Expression of ROS‐Scavenging Enzymes

ROS‐scavenging enzymes play important roles in ROS cellular homeostasis under normal and stressful conditions (Zhu et al. 2021). In order to determine whether MgONPs regulate ROS‐scavenging enzymes, we measured the activity of several ROS‐scavenging enzymes and the expression levels of genes related to ROS scavenging in MgONP‐treated *N. benthamiana* plants with TMV‐GFP infection in a 72‐h time course. Our results showed that the activities of CAT, POD, SOD, PPO, and PAL were significantly increased in MgONP‐treated plants without TMV‐GFP infection compared with those in water‐treated plants (Figure S13a–e). Furthermore, the activities of CAT, POD, SOD, PPO, and PAL were markedly enhanced in MgONPs‐treated plants inoculated with TMV‐GFP in a 72‐h time course compared to the controls (Figure S13a–e). RT‐qPCR results showed that the expression levels of *NbCAT1*, *NbCu/Zn‐SOD*, *NbFeSOD*, *NbAPX1*, *NbAPX3*, *NbAPX4*, *NbAPX5*, and *NbAPX7* were all significantly enhanced in MgONP‐treated plants with TMV‐GFP inoculation in a 72‐h time course compared to those in water‐treated plants (Figure S13f–m).

### 2.10 Application of MgONPs Induces Partial Resistance in *NbRboh* ‐Silenced Plants

**FIGURE 4:**
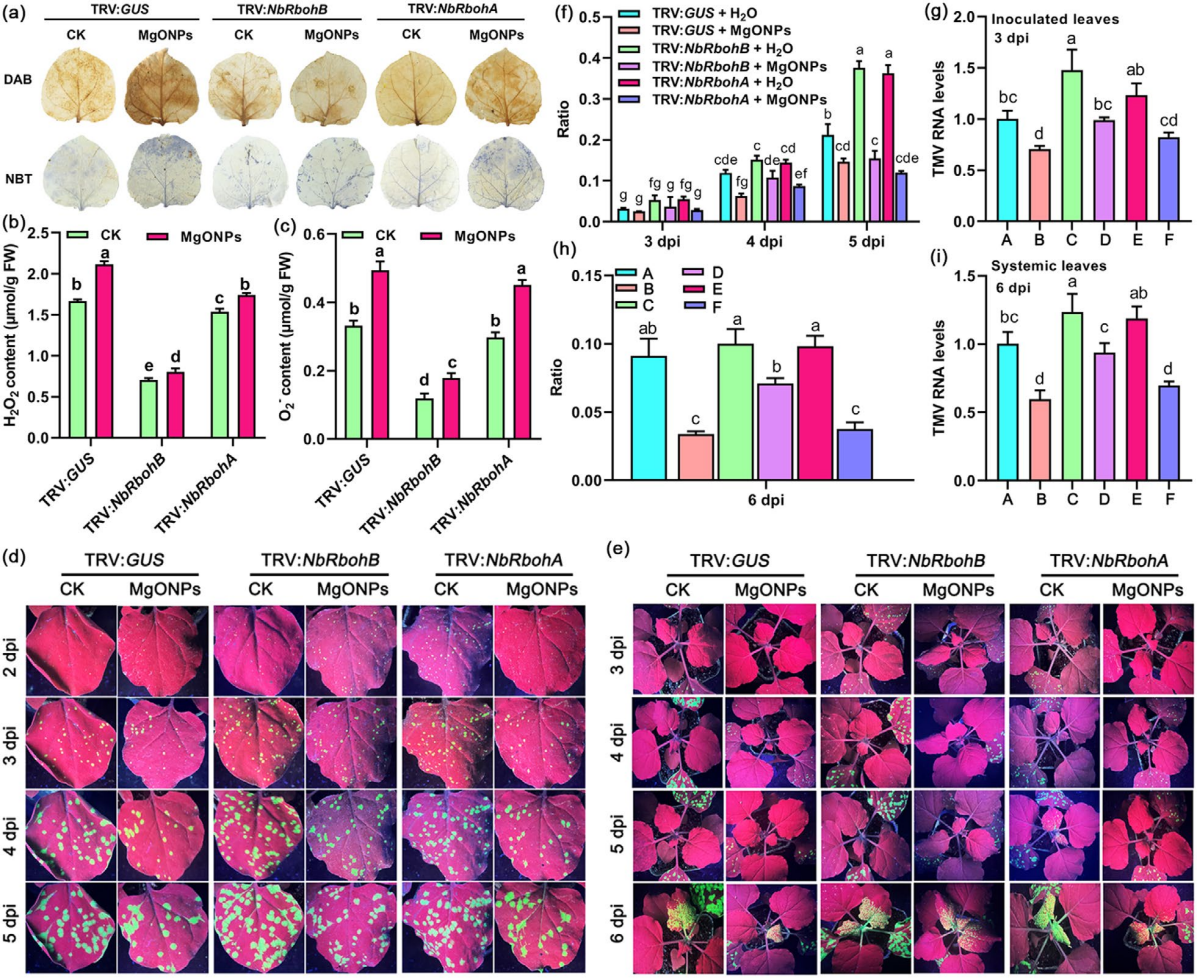
MgONPs induce partial resistance in *NbRboh*‐silenced plants. (a) The levels of H_2_O_2_ and O_2_˙^−^ were visualised by DAB and NBT staining, respectively, in *NbRbohA*‐ or *NbRbohB‐*silenced plants under MgONPs and water treatments. (b, c) The H_2_O_2_ (b) and O_2_˙^−^ (c) contents were measured in leaves of *NbRbohA*‐ or *NbRbohB‐*silenced plants under MgONPs and water treatments by a Hydrogen Peroxide Assay Kit and a Micro Superoxide Anion Assay Kit, respectively. (d) Representative images of GFP fluorescence visualised in the inoculated leaves of *NbRbohA*‐ or *NbRbohB‐*silenced plants under MgONPs and water treatments at different time points after infection with TMV‐GFP. (e) Representative images of GFP fluorescence visualised in the systemic leaves of *NbRbohA*‐ or *NbRbohB‐*silenced plants under MgONPs and water treatments at different time points after infection with TMV‐GFP. (f) The ratio of the GFP fluorescent area to the total area of the inoculated leaves shown in (d) at 3–5 dpi. (g) RT‐qPCR analysis of the TMV RNA levels in the inoculated leaves of *NbRbohA*‐ or *NbRbohB‐*silenced plants under MgONPs and water treatments at 3 dpi after infection with TMV‐GFP. *Actin* was used as the internal reference gene, and the expression is relative to that in the TRV:*GUS* with ddH_2_O treatment at 3 dpi, the value of which was set as 1. (h) The ratio of the GFP fluorescent area to the total area of the systemic leaves shown in (e) at 6 dpi. (i) TMV RNA levels in the systemic leaves of *NbRbohA*‐ or *NbRbohB‐*silenced plants under MgONPs and water treatments at 6 dpi after infection with TMV‐GFP, as determined by RT‐qPCR. *Actin* was used as the internal reference gene, and the expression is relative to that in the TRV:*GUS* with ddH_2_O treatment at 6 dpi, the value of which was set as 1. A: TRV:*GUS* + H_2_O, B: TRV:*GUS* + MgONPs, C: TRV:*Nbrbohb* + H_2_O, D: TRV:*Nbrbohb* + MgONPs, E: TRV:*Nbrboha* + H_2_O, F: TRV:*Nbrboha* + MgONPs. Different letters indicate significant differences as determined using one‐way ANOVA followed by Tukey's test between multiple groups (*p* < 0.05).

*NbRbohA* or *NbRbohB*‐silenced plants were treated with MgONPs, and then TMV‐GFP was infected to verify if MgONPs induce plant immunity in these plants. There was no discernible phenotypic change between the *NbRbohA*‐ or *NbRbohB*‐silenced plants and the control (TRV:*GUS*) (Figure S14a). The gene‐silencing efficiency of *NbRbohA* or *NbRbohB* at 12 d post‐infiltration was examined using RT‐qPCR. Results showed that the transcript levels of *NbRbohA* and *NbRbohB* were significantly reduced in *NbRbohA*‐ or *NbRbohB*‐silenced plants compared to the controls (Figure S14b,c). ROS staining assays showed that the levels of H_2_O_2_ and O_2_˙^−^ were significantly decreased in *NbRbohA*‐ or *NbRbohB*‐silenced plants treated with MgONPs compared with control plants treated with MgONPs (Figure 4a). Direct measurements of H_2_O_2_ and O_2_˙^−^ also confirmed that their contents were markedly reduced in *NbRbohB*‐silenced plants pretreated with MgONPs compared with the control plants (TRV:*GUS*) pretreated with MgONPs (Figure 4b,c). There was a significant increase in the number of GFP fluorescent foci in the inoculated leaves and systemic leaves of the *NbRbohA‐* or *NbRbohB*‐silenced plants compared to control plants (TRV:*GUS*) with only water treatment in a 5–6‐day time course after TMV‐GFP infection (Figure 4d–f,h). RT‐qPCR results also showed that TMV RNA levels in the inoculated leaves and systemic leaves of the *NbRbohA*‐ or *NbRbohB*‐silenced plants were also enhanced at these time points compared to the control plants (Figure 4g,i), indicating that silencing of *NbRbohA* or *NbRbohB* compromises plant resistance to TMV. In addition, the results of GFP fluorescence imaging (Figure 4d,e), GFP fluorescent foci quantification (Figure 4f,h), and RT‐qPCR (Figure 4g,i) in a 5–6‐day time course all confirmed that reduced viral accumulation occurred in the inoculated leaves and systemic leaves of *NbRbohA‐* or *NbRbohB*‐silenced plants pretreated with MgONPs compared to those pretreated with water. Reduced viral accumulation occurred in the *NbRbohA*‐ or *NbRbohB*‐silenced plants pretreated with MgONPs; however, the levels of TMV were the least in control plants (TRV:*GUS*) pretreated with MgONPs (Figure 4d–i). Overall, these results confirmed that exogenous application of MgONPs could trigger partial resistance in *NbRboh*‐silenced plants.

### 2.11 Exogenous Application of MgONPs Activates SA‐, JA‐ and ET‐Mediated Signalling Pathways

SA‐, JA‐ and ET‐mediated signalling pathways are central regulators of plant defence (Berens et al. 2017). Several studies suggest that Ca^2+^ influx through glutamate receptor‐gated channels is involved in SA, JA, and ET biosynthesis pathways (Ludwig et al. 2005). Calmodulin (CaM)‐binding protein CBP60g, guanosine triphosphate (GTP)‐binding protein Gα, and Ca^2+^‐dependent protein kinase CDPK2 positively regulate the expression of SA, JA, ET biosynthetic genes *ICS1*, *OPR3*, and *ACCOx*, respectively (Vidhyasekaran 2015; Ludwig et al. 2005; Zhang et al. 2010). Thus, we examined the transcript levels of *NbCBP60g*, *NbGα*, and *NbCDPK2* in MgONP‐treated plants in a 24‐h time course. The results showed that the expression levels of *NbCBP60g*, *NbGα*, and *NbCDPK2* were all significantly enhanced in MgONP‐treated plants compared with water‐treated plants (Figure S15).

We next examined the endogenous SA, JA, and ET contents, the expression of SA, JA, and ET biosynthetic and signalling genes, and the expression of SA, JA, and ET‐related defence genes. The MgONP‐pretreated plants showed significantly higher SA, JA and ET contents than water‐treated plants (CK) (Figure S16a–c). The expression of the SA biosynthetic and signalling genes *NbICS1* (Figure S16d), *NbNPR1* (Figure S16e), *NbTGA2.1* (Figure S16f), and *NbTGA2.2* (Figure S16g), JA biosynthetic and signalling genes *NbOPR3* (Figure S16k), *NbCOI1* (Figure S16l), and *NbMYC2* (Figure S16m), ET biosynthetic and signalling genes *NbACCOx* (Figure S16p), *NbEIN2* (Figure S16q), and *NbETR1* (Figure S16r) was also significantly increased in the MgONPs‐treated plants. In addition, the transcript levels of the SA‐related defence genes *NbPR1* (Figure S16h), *NbPR2* (Figure S16i), and *NbPR5* (Figure S16j), JA‐mediated defence genes *NbPDF1.2* (Figure S16n) and *NbPR3* (Figure S16o), and ET‐related defence gene *NbPR4* (Figure S16s) were all significantly higher in MgONP‐treated plants than in water‐treated plants (CK). Thus, these results confirmed that foliar application of MgONPs activates SA‐, JA‐, and ET‐mediated signalling defence pathways.

### 2.12 Exogenous Application of MgONPs Stimulates Partial Resistance in SA‐Deficient Plants

**FIGURE 5:**
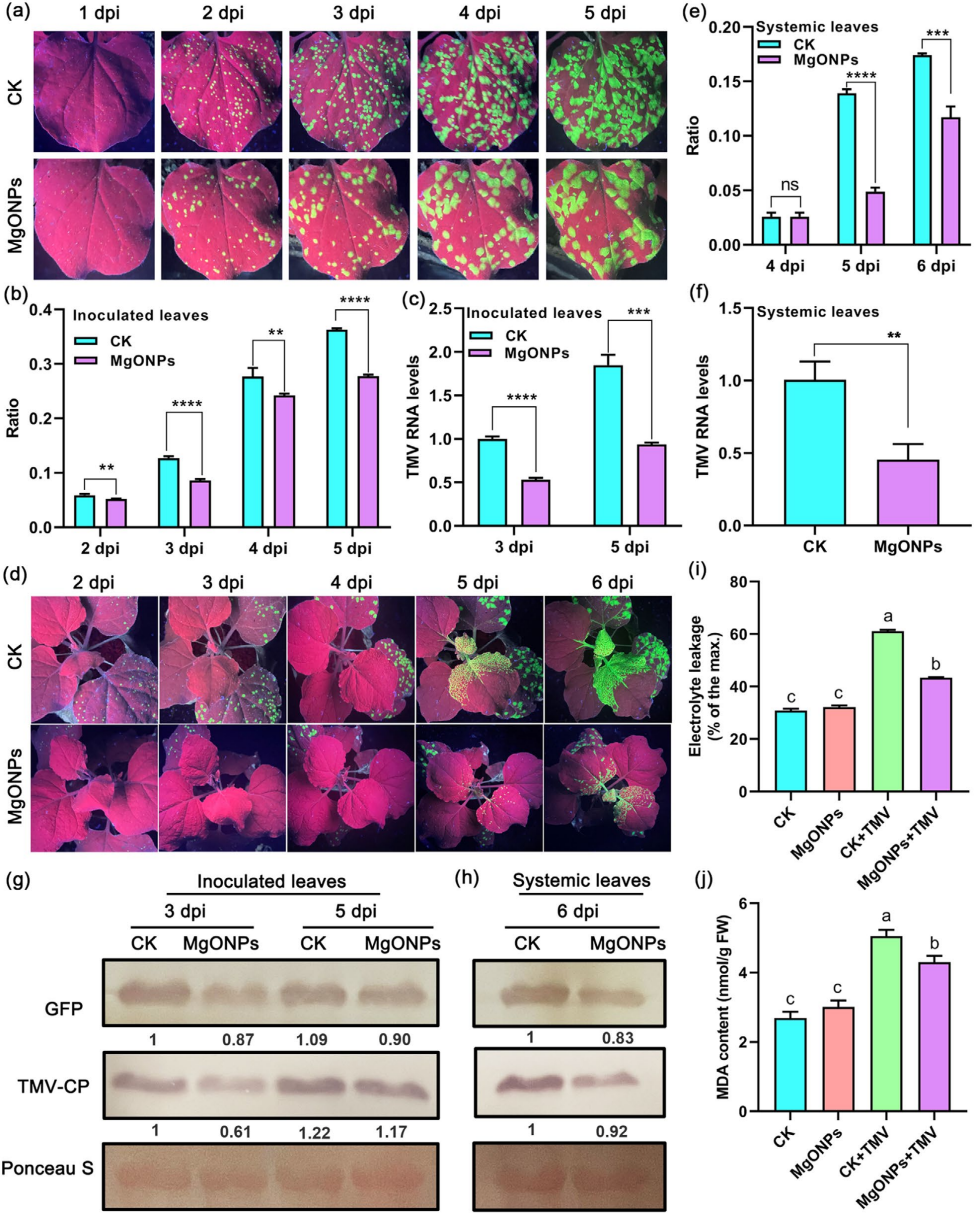
Exogenous application of MgONPs stimulates partial resistance in SA‐deficient plants. (a) Representative images of GFP fluorescence visualised in the inoculated leaves of *NahG*‐transgenic plants under MgONPs or water treatments at different time points after infection with TMV‐GFP. (b) The ratio of the GFP fluorescent area to the total area of the inoculated leaves shown in (a) at 2–5 dpi. (c) TMV RNA levels in the inoculated leaves shown in (a) at 3 and 5 dpi, as determined by RT‐qPCR. *Actin* was used as the internal reference gene, and the expression is relative to that in the *NahG* plants with ddH_2_O treatment at 3 dpi, the value of which was set as 1. (d) Representative images of GFP fluorescence visualised in the systemic leaves of *NahG*‐transgenic plants under MgONPs or water treatments at different time points after infection with TMV‐GFP. (e) The ratio of the GFP fluorescent area to the total area of the systemic leaves shown in (d) at 4–6 dpi. (f) TMV RNA levels in the systemic leaves shown in (d) at 6 dpi, as determined by RT‐qPCR. *Actin* was used as the internal reference gene, and the expression is relative to that in the *NahG* plants with ddH_2_O treatment at 6 dpi, the value of which was set as 1. (g) Western blotting of the accumulation of TMV coat proteins in the inoculated leaves shown in (a) at 3 and 5 dpi. Rubisco proteins were used as loading controls and were stained by Ponceau S. (h) Western blotting of the accumulation of TMV coat proteins in the systemic leaves shown in (d) at 6 dpi. Rubisco proteins were used as loading controls and were stained by Ponceau S. (i, j) Electrolyte leakage (i) and MDA contents (j) measured in the leaves of *NahG*‐transgenic plants under MgONPs and water treatments at 3 dpi with TMV‐GFP. Asterisks represent significant differences determined by Student's *t* test between two groups (**p < 0.01, ***p < 0.001, ****p < 0.0001). Different letters indicate significant differences as determined using one‐way ANOVA followed by Tukey's test between multiple groups (*p* < 0.05).

To determine whether MgONPs induce resistance in SA‐deficient plants, *NahG* (encoding the SA‐degrading enzyme salicylate hydroxylase) transgenic *N. benthamiana* plants were sprayed with MgONPs, after which TMV‐GFP was inoculated. There was no significant phenotypic difference between MgONPs‐treated *NahG* plants and water‐treated *NahG* plants without TMV‐GFP (Figure S17). There was a significant decrease in the number of GFP fluorescent foci in the inoculated leaves and systemic leaves of MgONP‐treated *NahG* plants compared to *NahG* plants with water treatment in a 5–6‐day time course after TMV‐GFP infection (Figure 5a,b,d,e). RT‐qPCR results also showed that TMV RNA levels in the inoculated leaves and systemic leaves of MgONPs‐treated *NahG* plants were reduced at these time points compared to control plants (Figure 5c,f). In addition, western blotting indicated that the protein levels of TMV‐CP and GFP were also reduced in inoculated leaves of MgONPs‐treated *NahG* plants in comparison with CK at 3 dpi and 5 dpi (Figure 5g). We further monitored the TMV replication and spread in the systemic upper leaves at 6 dpi using western blot. The results confirmed that reduced viral accumulation was observed in upper systemic leaves of MgONPs‐treated *NahG* plants compared with CK (Figure 5h).

There was no obvious difference in electrolyte leakage and MDA contents between MgONPs‐treated *NahG* plants and water‐treated *NahG* plants (CK) without TMV‐GFP (Figure 5i,j). Electrolyte leakage and MDA contents were markedly increased in water‐treated *NahG* plants with TMV‐GFP infection (Figure 5i,j). However, the level of electrolyte leakage and MDA contents was significantly decreased in MgONP‐treated *NahG* plants compared with CK at 3 days after TMV‐GFP inoculation, implying that the cytomembranes of MgONPs‐treated *NahG* plants suffered less oxidative damage under TMV‐GFP infection (Figure 5i,j). DAB and NBT staining assays showed that the levels of H_2_O_2_ and O_2_˙^−^ were significantly increased in both MgONP‐treated *NahG* plants and water‐treated *NahG* plants at 3 dpi with TMV‐GFP infection (Figure S18a). However, the levels of H_2_O_2_ and O_2_˙^−^ were markedly lower in MgONPs‐treated *NahG* plants in comparison with CK at 3 dpi under TMV‐GFP infection (Figure S18a). Direct measurements of H_2_O_2_ and O_2_˙^−^ showed that their contents were significantly reduced in MgONP‐treated *NahG* plants compared with water‐treated *NahG* plants at 3 dpi with TMV‐GFP (Figure S18b,c). Visualisation of H_2_O_2_ and fluorescence quantification confirmed that the levels of H_2_O_2_ were clearly increased in both MgONP‐treated *NahG* plants and water‐treated *NahG* plants at 3 dpi with TMV‐GFP infection; however, MgONP‐treated *NahG* plants showed lower fluorescence intensity than the controls after TMV‐GFP infection (Figure S18d,e).

Phytohormone measurement results showed that JA and ET contents were significantly enhanced in MgONP‐treated *NahG* plants compared with water‐treated *NahG* plants (CK) (Figure S18f,g). RT‐qPCR results indicated that the expression of the JA biosynthetic gene *NbOPR3* (Figure S18h) and signalling gene *NbCOI1* (Figure S18i) and ET biosynthetic gene *NbACCOx* (Figure S18l) and signalling gene *NbEIN2* (Figure S18m) was significantly increased in MgONP‐treated *NahG* plants. In addition, the transcript levels of the JA‐mediated defence gene *NbPR3* (Figure S18k) and ET‐related defence gene *NbPR4* (Figure S18n) were significantly higher in MgONPs‐treated *NahG* plants than in water‐treated *NahG* plants (CK). Thus, our results confirmed that foliar application of MgONPs activates JA‐ and ET‐mediated signalling defence pathways and triggers partial resistance in SA‐deficient plants against viral pathogens.

### 2.13 Foliar Application of MgONPs Induces Partial Resistance in JA‐Deficient Plants

**FIGURE 6:**
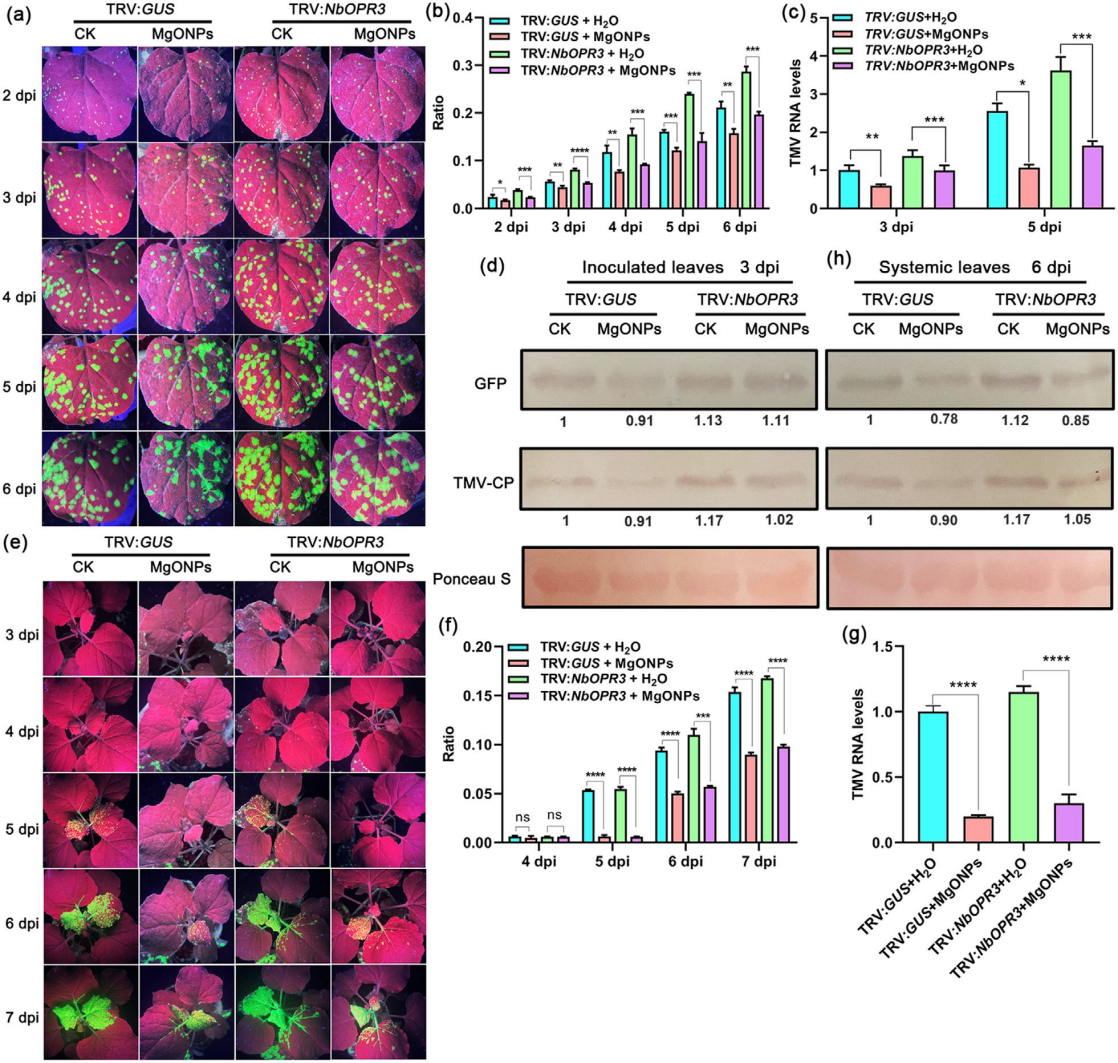
Foliar application of MgONPs induces partial resistance in *NbOPR3*‐silenced *N. benthamiana* plants. (a) Representative images of GFP fluorescence visualised in the inoculated leaves of *NbOPR3*‐silenced *N. benthamiana* plants under MgONPs or water treatments at different time points after infection with TMV‐GFP. (b) The ratio of the GFP fluorescent area to the total area of the inoculated leaves shown at 2–6 dpi. (c) TMV RNA levels in the inoculated leaves shown in (a) at 3 dpi and 5 dpi, as determined by RT‐qPCR. *Actin* was used as the internal reference gene, and the expression is relative to that in the TRV:*GUS* with ddH_2_O treatment at 3 dpi, the value of which was set as 1. (d) Western blotting of the accumulation of TMV coat proteins in the inoculated leaves shown in (a) at 3 and 5 dpi. Rubisco proteins were used as loading controls and were stained by Ponceau S. (e) Representative images of GFP fluorescence visualised in the systemic leaves of *NbOPR3*‐silenced *N. benthamiana* plants under MgONPs or water treatments at different time points after infection with TMV‐GFP. (f) The ratio of the GFP fluorescent area to the total area of the systemic leaves shown in (e) at 4–7 dpi. (g) RT‐qPCR analysis of the TMV RNA levels in the systemic leaves shown in (e) at 6 dpi. *Actin* was used as the internal reference gene, and the expression is relative to that in the TRV:*GUS* with ddH_2_O treatment at 6 dpi, the value of which was set as 1. (h) Western blotting of the accumulation of TMV coat proteins in the systemic leaves shown in (e) at 6 dpi. Rubisco proteins were used as loading controls and were stained by Ponceau S. Asterisks represent significant difference determined by Student's *t* test (*p < 0.05, **p < 0.01, ***p < 0.001, ****p < 0.0001).

**FIGURE 7:**
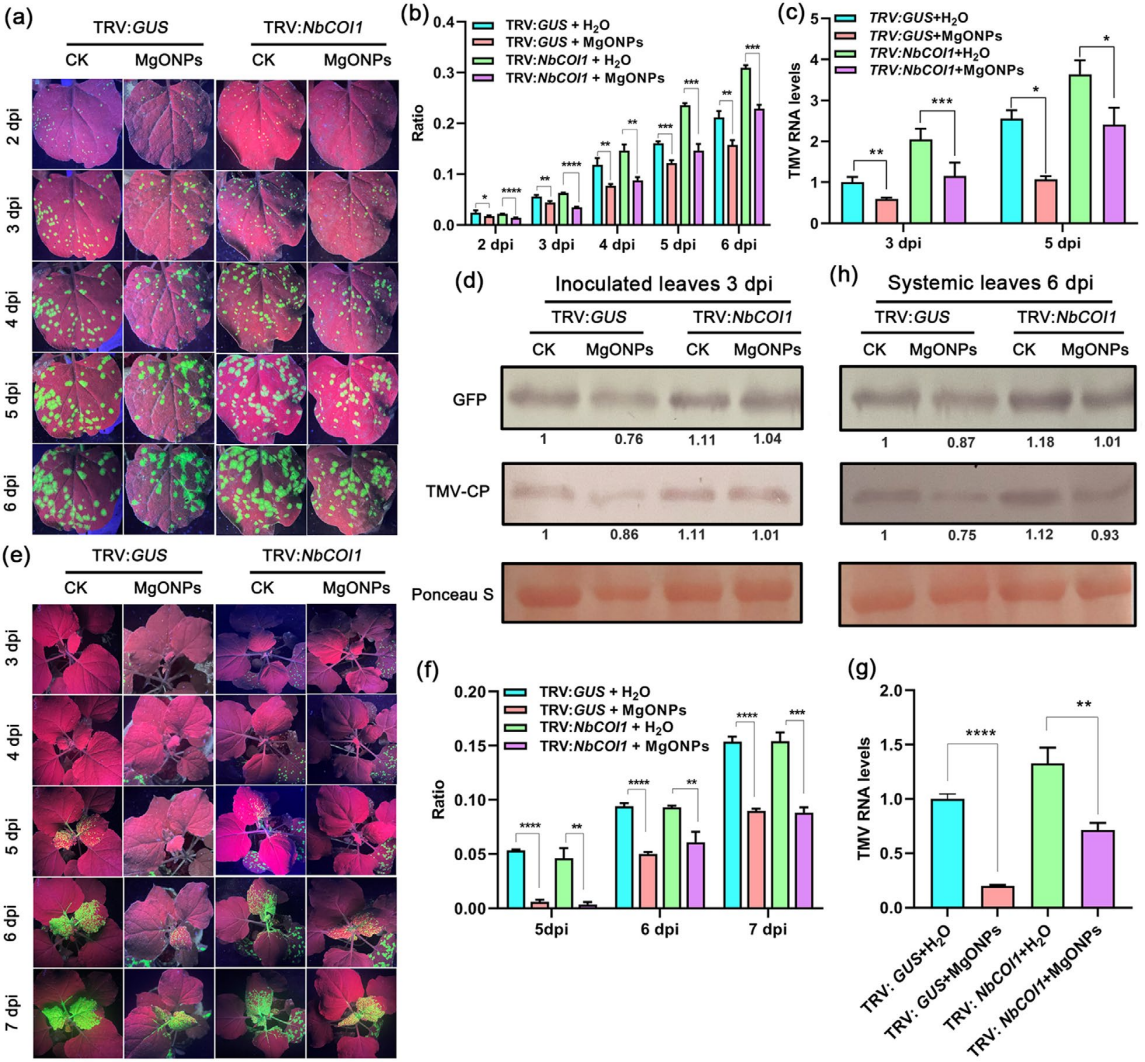
Foliar application of MgONPs triggers partial resistance in *NbCOI1*‐silenced *N. benthamiana* plants. (a) Representative images of GFP fluorescence visualised in the inoculated leaves of *NbCOI1*‐silenced *N. benthamiana* plants under MgONPs or water treatments at different time points after infection with TMV‐GFP. (b) The ratio of the GFP fluorescent area to the total area of the inoculated leaves shown in (a) at 2–6 dpi. (c) TMV RNA levels in the inoculated leaves shown in (a) at 3 dpi and 5 dpi, as determined by RT‐qPCR. *Actin* was used as the internal reference gene, and the expression is relative to that in the TRV:*GUS* with ddH_2_O treatment at 3 dpi, the value of which was set as 1. (d) Western blotting of the accumulation of TMV coat proteins in the inoculated leaves shown in (a) at 3 and 5 dpi. Rubisco proteins were used as loading controls and were stained by Ponceau S. (e) Representative images of GFP fluorescence visualised in the systemic leaves of *NbCOI1*‐silenced *N. benthamiana* plants under MgONPs or water treatments at different time points after infection with TMV‐GFP. (f) The ratio of the GFP fluorescent area to the total area of the systemic leaves shown in (e) at 5–7 dpi. (g) TMV RNA levels in the systemic leaves shown in (e) at 6 dpi, as determined by RT‐qPCR. *Actin* was used as the internal reference gene, and the expression is relative to that in the TRV:*GUS* with ddH_2_O treatment at 6 dpi, the value of which was set as 1. Asterisks represent significant difference determined by Student's *t* test (*p < 0.05, **p < 0.01, ***p < 0.001, ****p < 0.0001).

To examine whether MgONPs activate plant immunity in JA‐deficient plants, we employed TRV‐mediated VIGS to suppress the expression of JA biosynthetic or signalling genes *NbOPR3* or *NbCOI1* in *N. benthamiana*. *NbOPR3*‐ or *NbCOI1*‐silenced *N. benthamiana* plants were sprayed with MgONPs, then TMV‐GFP was inoculated. No obvious phenotypic difference was observed between *NbOPR3*‐ (Figure S19a) or *NbCOI1*‐silenced plants (Figure S20a) and the control (TRV:*GUS*) plants. The gene‐silencing efficiency of *NbOPR3* or *NbCOI1* at 12 d post‐infiltration was examined using RT‐qPCR. Results showed that the transcript levels of *NbOPR3* (Figure S19b) or *NbCOI1* (Figure S20b) were significantly reduced in *NbOPR3‐* or *NbCOI1*‐silenced plants compared to the controls. There was a significant increase in the number of GFP fluorescent foci in the inoculated leaves and systemic leaves of the *NbOPR3*‐ (Figure 6a,b,e,f) or *NbCOI1*‐silenced plants (Figure 7a,b,e,f) compared to the control plants (TRV:*GUS*) with only water treatment in a 6–7‐day time course after TMV‐GFP infection. RT‐qPCR and western blotting results also showed that the levels of TMV RNA and TMV‐CP in the inoculated leaves and systemic leaves of the *NbOPR3*‐ (Figure 6c,d,g,h) or *NbCOI1*‐silenced plants (Figure 7c,d,g,h) were also enhanced at these time points compared to the control plants, indicating that silencing of *NbOPR3* or *NbCOI1* compromises plant resistance to TMV. In addition, the results of GFP fluorescence imaging (Figure 6a,e and Figure 7a,e), GFP fluorescent foci quantification (Figure 6b,f and Figure 7b,f), RT‐qPCR (Figure 6e,g and Figure 7e,g), and western blotting (Figure 6d,h and Figure 7d,h) at 5–6‐day time course all confirmed that reduced viral accumulation occurred in the inoculated leaves and systemic leaves of *NbOPR3*‐ or *NbCOI1*‐silenced plants pretreated with MgONPs compared to those pretreated with water only. Reduced viral accumulation occurred in *NbOPR3*‐ or *NbCOI1*‐silenced plants pretreated with MgONPs; however, the levels of TMV were the least in the control plants (TRV:*GUS*) pretreated with MgONPs (Figure 6 and Figure 7). Overall, these results confirmed that the exogenous application of MgONPs could activate partial plant immunity in JA‐deficient plants against virus infection.

### 2.14 MgONPs Activate Partial Resistance in ET‐Deficient Plants

**FIGURE 8:**
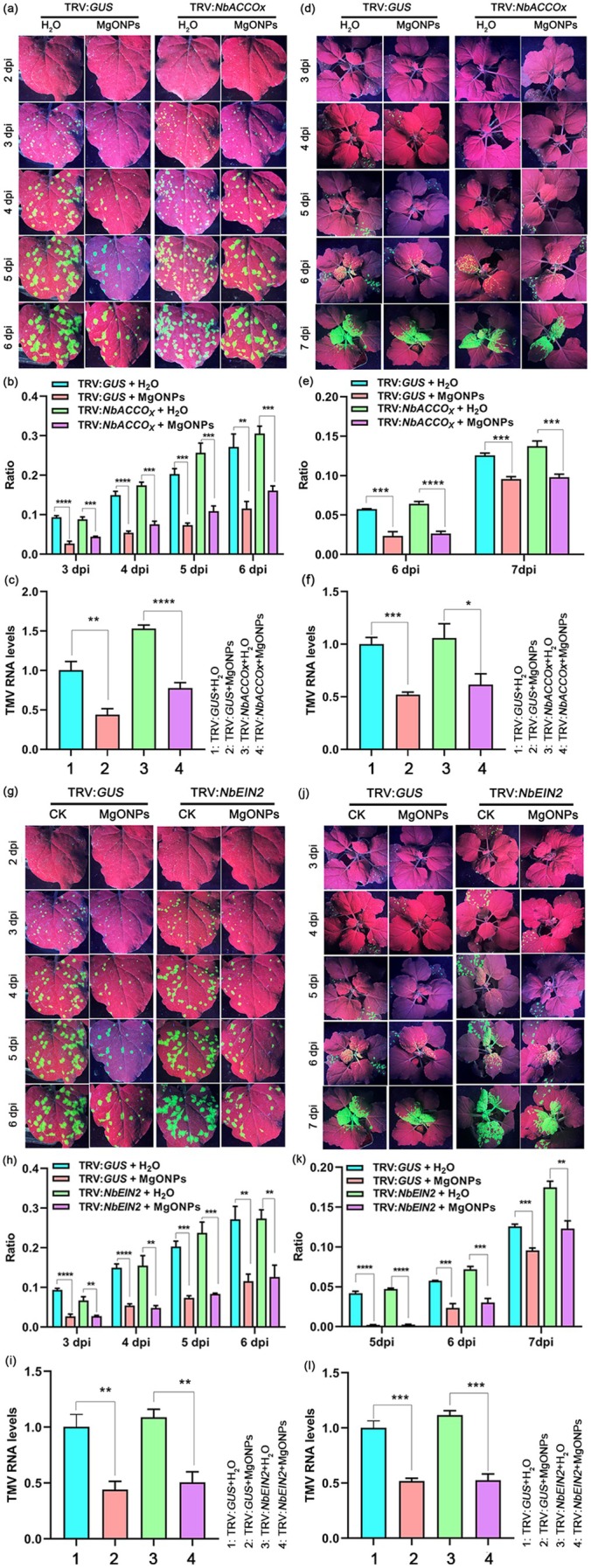
Foliar application of MgONPs induces partial resistance in *NbACCOx*‐ and *NbEIN2*‐silenced *N. benthamiana* plants. (a, g) Representative images of GFP fluorescence visualised in the inoculated leaves of *NbACCOx*‐ and *NbEIN2*‐silenced *N. benthamiana* plants under MgONPs or water treatments at different time points after infection with TMV‐GFP. (b, h) The ratio of the GFP fluorescent area to the total area of the inoculated leaves shown in (a, g) at 3–6 dpi. (c, i) TMV RNA levels in the inoculated leaves of the leaves shown in (a, g) at 3 dpi, as determined by RT‐qPCR. *Actin* was used as the internal reference gene, and the expression is relative to that in the TRV:*GUS* with ddH_2_O treatment at 3 dpi, the value of which was set as 1. (d, j) Representative images of GFP fluorescence visualised in the systemic leaves of *NbACCOx*‐ and *NbEIN2*‐silenced *N. benthamiana* plants under MgONPs or water treatments at different time points after infection with TMV‐GFP. (e, k) The ratio of the GFP fluorescent area to the total area of the systemic leaves shown in (d, j) at 6 dpi and 7 dpi. (f, l) TMV RNA levels in the systemic leaves of the leaves shown in (d, j) at 6 dpi, as determined by RT‐qPCR. *Actin* was used as the internal reference gene, and the expression is relative to that in the TRV:*GUS* with ddH_2_O treatment at 6 dpi, the value of which was set as 1. Asterisks represent significant difference determined by Student's *t* test (*p < 0.05, **p < 0.01, ***p < 0.001, ****p < 0.0001).

To investigate whether MgONPs induce plant immunity in ET‐deficient plants, *NbACCOx*‐ or *NbEIN2*‐silenced *N. benthamiana* plants were sprayed with MgONPs, then TMV‐GFP was inoculated. There was no significant phenotypic difference between *NbACCOx*‐ (Figure S21a) or *NbEIN2*‐silenced plants (Figure S22a) and the control (TRV:*GUS*) without TMV‐GFP infection. RT‐qPCR results showed that the transcript levels of *NbACCOx* (Figure S21b) or *NbEIN2* (Figure S22b) were significantly decreased in the *NbACCOx‐* or *NbEIN2*‐silenced plants compared to the controls. There was a significant increase in the number of GFP fluorescent foci in the inoculated leaves and systemic leaves of the *NbACCOx*‐ (Figure 8a,b,d,e) or *NbEIN2*‐silenced plants (Figure 8 g,h,j,k) compared to the control plants (TRV:*GUS*) with water treatment in a 6–7‐day time course after TMV‐GFP infection. RT‐qPCR results also showed that the TMV RNA levels in the inoculated leaves and systemic leaves of the *NbACCOx*‐ (Figure 8c,f) or *NbEIN2*‐silenced plants (Figure 8i,l) were also elevated at these time points compared to the control plants, indicating that the silencing of *NbACCOx* or *NbEIN2* compromises plant resistance to TMV. Interestingly, the results of GFP fluorescence imaging (Figure 8a,d,g,j), GFP fluorescent foci quantification (Figure 8b,e,h,k), and RT‐qPCR (Figure 8c,f,i,l) in a 5–6‐day time course, all confirmed that a reduced viral accumulation occurred in the inoculated leaves and systemic leaves of *NbACCOx*‐ or *NbEIN2*‐silenced plants pretreated with MgONPs compared to those pretreated with water only. Reduced viral accumulation occurred in the *NbACCOx*‐ or *NbEIN2*‐silenced plants pretreated with MgONPs; however, the levels of TMV were the least in the control plants (TRV:*GUS*) pretreated with MgONPs (Figure 8). Thus, these results confirmed that the exogenous application of MgONPs activates partial plant resistance in ET‐deficient plants against viral diseases.

### 2.15 MgONPs Fail to Induce Resistance to Viral Pathogens in Plants Simultaneously Lacking SA, JA, and ET

**FIGURE 9:**
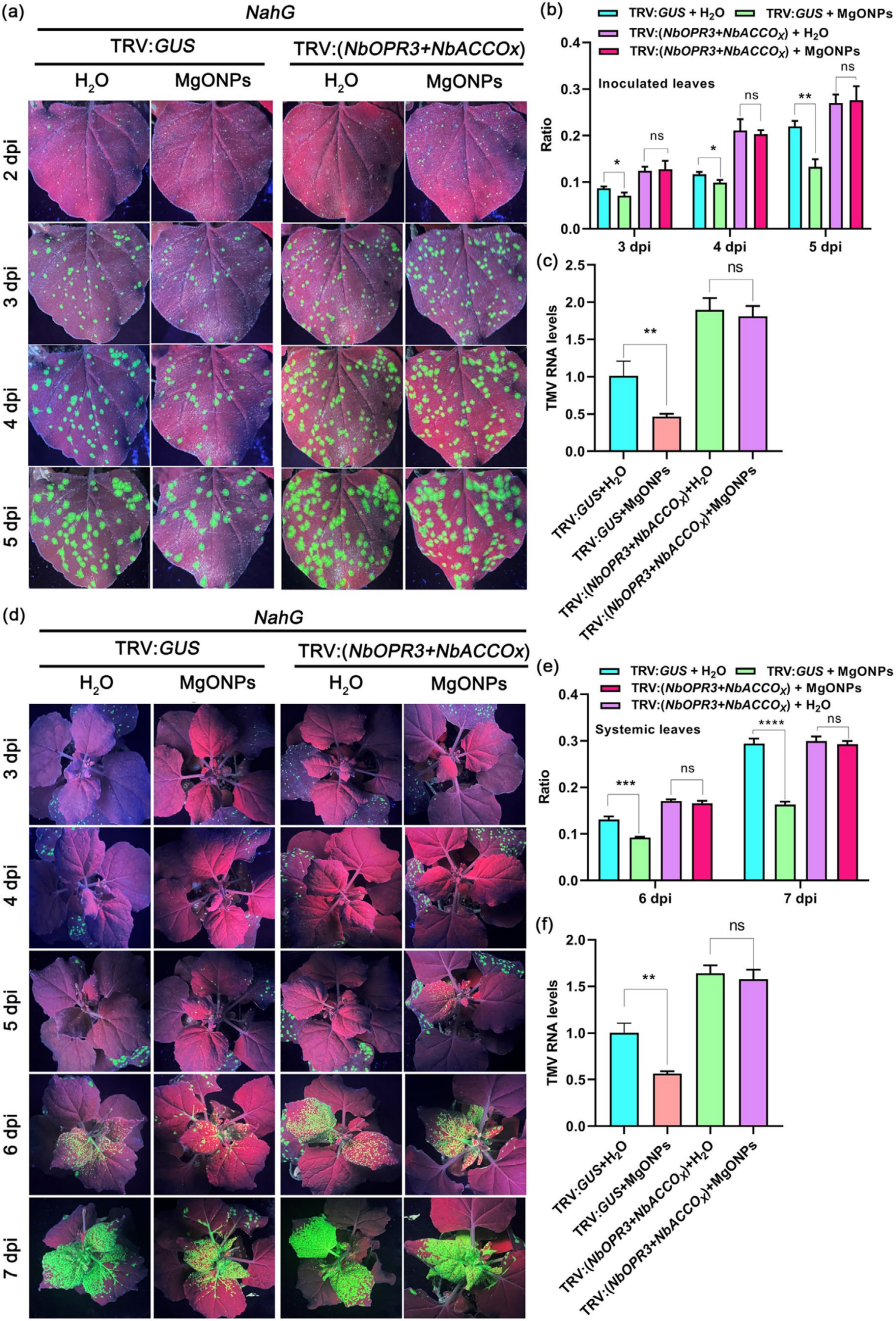
Application of MgONPs on plants simultaneously lacking SA, JA, and ET does not induce resistance to viral pathogens. (a) Representative images of GFP fluorescence visualised in the inoculated leaves of (*NbOPR3 + NbACCOx*)‐silenced *NahG* plants under MgONPs or water treatments at different time points after infection with TMV‐GFP. (b) The ratio of the GFP fluorescent area to the total area of the inoculated leaves shown in (a) at 3–5 dpi. (c) TMV RNA levels in the inoculated leaves of the leaves shown in (a) at 3 dpi, as determined by RT‐qPCR. *Actin* was used as the internal reference gene, and the expression is relative to that in the TRV:*GUS* with ddH_2_O treatment at 3 dpi, the value of which was set as 1. (d) Representative images of GFP fluorescence visualised in the systemic leaves of (*NbOPR3 + NbACCOx*)‐silenced *NahG* plants under MgONPs or water treatments at different time points after infection with TMV‐GFP. (e) The ratio of the GFP fluorescent area to the total area of the systemic leaves shown in (d) at 6 dpi and 7 dpi. (f) TMV RNA levels in the systemic leaves of the leaves shown in (d) at 6 dpi, as determined by RT‐qPCR. *Actin* was used as the internal reference gene, and the expression is relative to that in the TRV:*GUS* with ddH_2_O treatment at 6 dpi, the value of which was set as 1. Asterisks represent significant differences determined by Student's *t* test (*p < 0.05, **p < 0.01, ***p < 0.001, ****p < 0.0001).

To examine whether MgONPs trigger plant immunity in SA‐, JA‐ and ET‐deficient plants, we employed TRV‐mediated VIGS to simultaneously suppress the expression of JA and ET biosynthetic genes *NbOPR3* and *NbACCOx* in SA‐deficient (*NahG*) transgenic *N. benthamiana* plants. *NbOPR3*‐ and *NbACCOx*‐silenced *NahG* plants were sprayed with MgONPs, then TMV‐GFP was inoculated. No obvious phenotypic difference was observed between the *NbOPR3*‐ and *NbACCOx*‐silenced *NahG* plants and the control plants (TRV:*GUS*) (Figure S23a). RT‐qPCR results showed that the transcript levels of *NbOPR3* and *NbACCOx* were significantly reduced in the *NbOPR3*‐ and *NbACCOx*‐silenced *NahG* plants compared to the controls (Figure S23b,c). There was a significant increase in the number of GFP fluorescent foci in the inoculated leaves and systemic leaves of the *NbOPR3*‐ and *NbACCOx*‐silenced *NahG* plants compared to the control *NahG* plants (TRV:*GUS*) with water treatment in a 5–7‐day time course after TMV‐GFP infection (Figure 9a,b,d,e). RT‐qPCR results also showed that the TMV RNA levels in the inoculated leaves and systemic leaves of the *NbOPR3*‐ and *NbACCOx*‐silenced *NahG* plants were enhanced at these time points compared to the control *NahG* plants (Figure 9c,f). Interestingly, the results of GFP fluorescence imaging (Figure 9a,d), GFP fluorescent foci quantification (Figure 9b,e), and RT‐qPCR (Figure 9c,f) in the 5–7‐day time course, all confirmed that there was no difference in viral accumulation in the inoculated leaves and systemic leaves of *NbOPR3*‐ and *NbACCOx*‐silenced *NahG* plants pretreated with MgONPs compared to those pretreated with water only. Taken together, these results confirmed that the exogenous application of MgONPs fails to trigger resistance in SA‐, JA‐, and ET‐deficient plants.

### 2.16 MgONPs Promote Plant Growth

In order to investigate whether MgONPs coordinate both plant defence and growth, we examined the growth performance of *N. benthamiana* plants treated with different concentrations of MgONPs (0, 100, 150, and 250 μg/mL). Our results showed that exogenous application of MgONPs significantly improved the agronomic traits compared to water‐treated plants (Figure S24a). The *N. benthamiana* plants grown with MgONPs showed an increase in root length (Figure S24b), plant height (Figure S24c), leaf areas (Figure S24d), fresh weight (Figure S24e), dry weight (Figure S24f), Chl*a* content (Figure S24g), Chl*b* content (Figure S24h), and total Chl content (Figure S24i) compared to the control plants. These results suggest that MgONP treatment enhances the overall growth and productivity of *N. benthamiana* plants.

### 2.17 MgONPs Induce Broad‐Spectrum Resistance Against Major Viral Diseases of Vegetable Crops

Viral diseases of vegetable crops seriously threaten the yield and quality of agricultural products (Jones and Naidu 2019). In order to investigate whether MgONPs activate broad‐spectrum virus resistance against diverse viral diseases of vegetable crops, *N. benthamiana* and *Solanum lycopersicum* (tomato) plants were sprayed with MgONPs, and then, respectively, infected with TuMV‐GFP, ToMV‐GFP, PVY‐GFP, PVX‐GFP, TSWV‐GFP, and TMV‐GFP. There was a significant decrease in the number of GFP fluorescent foci in the inoculated leaves (Figure S25a,b) and systemic leaves (Figure S25c,d) of MgONP‐treated *N. benthamiana* plants compared to water‐treated plants (CK) in a 3–7‐day time course after TuMV‐GFP infection. Likewise, GFP fluorescence imaging and GFP fluorescent foci quantification confirmed that reduced viral accumulation occurred in the inoculated leaves (Figure S25e–h) and systemic leaves (Figure S25i,j) of *N. benthamiana* plants pretreated with MgONPs compared to the CK in a 3–7‐day time course after ToMV‐GFP infection (Figure S25e–j). In addition, for PVY, PVX, and TSWV, a significant decrease in the number of GFP fluorescent foci was observed in the inoculated leaves and systemic leaves of MgONPs‐treated *N. benthamiana* plants compared to the CK in a 3–7‐day time course after PVY‐GFP, PVX‐GFP, or TSWV‐GFP infection (Figure S25k–t).

Next, we found that there was a significant decrease in the number of GFP fluorescent foci in the inoculated leaves of MgONP‐treated tomato plants compared to water‐treated plants (CK) in a 10‐day time course after TMV‐GFP infection (Figure S26a,b). GFP fluorescence imaging and GFP fluorescence quantification confirmed that reduced viral accumulation occurred in the inoculated leaves of tomato plants pretreated with MgONPs compared to the CK in a 3‐day time course after ToMV‐GFP (Figure S26c,d), TuMV‐GFP (Figure S26e,f), or PVX‐GFP (Figure S26g,h) infection. Overall, these results suggest that the exogenous application of MgONPs triggers broad‐spectrum resistance against major viral diseases of vegetable crops.

### 2.18 Safety Evaluation of MgONPs in Mung Bean Plants

Recently, the potential ecotoxicity of nanomaterials has attracted rising concern. Hence, to investigate the potential ecotoxicity of MgONPs, the toxicity assessment of MgONPs was performed in plants and animals. First, the phytotoxicity of MgONPs was evaluated with mung bean ( *Vigna radiata* L.) by assessing seed germination, shoot and root growth under MgONPs treatment, which are the more frequently used variables to assess the effects of plant exposure to harmful substances (Ruttkay‐Nedecky et al. 2017; Vijayakumar et al. 2013). Our results indicate that there was no difference in the seed germination of mung beans between various concentrations of MgONPs (50, 150, 250, 500 μg/mL) treatment and CK (Figure S27a,b). In addition, no obvious difference in root length (Figure S27a,c), shoot length (Figure S27a,d) and fresh weight (Figure S27e) was observed between different concentrations of MgONPs (50, 150, 250 μg/mL)‐treated and water‐treated mung bean seedlings. However, when the concentration of MgONPs was 500 μg/mL, root lengths (Figure S27a,c), shoot lengths (Figure S27a,d) and fresh weight (Figure S27e) were significantly decreased in MgONP‐treated mung bean seedlings compared to the CK. The phytotoxicity assessment of MgONPs confirmed that there were no significant effects on seed germination, shoot and root lengths, and fresh weight of the mung bean. MgONPs affected shoot and root length, and fresh weight of the mung bean at a high concentration (500 μg/mL). Thus, our results demonstrated that MgONPs have desirable biocompatibility and biosafety on mung bean plants.

### 2.19 In Vivo Safety Evaluation of MgONPs in Zebrafish

Next, the biosafety of MgONPs was evaluated using zebrafish, a well‐established model organism for in vivo toxicity assessment (Figure S27f–i). Throughout the experiment, no fish died in the control treatment (Figure S27f–i). After 24, 48, 72, and 96 h of incubation, the survival of zebrafish incubated with MgONPs at 400 mg/mL was approximately 87% (Figure S27f–i). On the basis of the concentration‐response curves, the median lethal concentration (LC_50_) values of MgONPs at 24 (Figure S27f), 48 (Figure S27g), 72 (Figure S27h), and 96 h (Figure S27i) toward zebrafish were calculated as 0.77, 0.54, 0.45, and 0.41 mg/mL. According to the obtained LC_50_ values, the toxicity grade of MgONPs on zebrafish was defined as lowly toxic (≥ 10 mg/L). Overall, our results indicated that MgONPs have satisfactory biosafety in the aquatic environment at the given dosage.

## 3 Discussion

### 3.1 MgONPs Elevates Plant Growth and Triggers Dose‐Dependent Plant Immunity Against Viral Pathogens

It has been proven that many nanomaterials (NMs) can improve plant growth and development and increase plant immunity against pathogens and pests (Fu et al. 2019; Dos Santos et al. 2024). Choudhary et al. (2017) found that Cu‐chitosan nanoparticle treatments in maize not only improved the growth in terms of chlorophyll content, plant height, root length, stem diameter, and grain yield, but also increased the resistance to Curvularia leaf spot (CLS) disease. Likewise, the application of functional carbon nanoparticles (FCN) to roots promoted the growth and development of rice and Arabidopsis, which also significantly increased the yields (Guo et al. 2023). The yield of rice increased by 31.1% and the nutritional quality improved by 6.4%–7.2% by foliar application of selenium nanomaterials (Chen et al. 2024). Arabidopsis plants primed with single‐walled carbon nanotubes (SWNTs) showed a 29% improvement in pathogen resistance (Cui et al. 2024). Previous studies have indicated that MgONPs affect the growth and development of various plant species, such as black gram, mung bean, mustard, lentils, horse gram, and chickpea (Gautam et al. 2023). Therefore, MgONPs have great potential to be developed into nano‐fertilisers. Nevertheless, it is still unclear whether MgONPs affect the growth and development of tobacco. In this study, we investigated the effects of MgONPs on the growth of *N. benthamiana* plants. The growth parameters (root length, plant height, fresh weight, dry weight, and Chl content) of *N. benthamiana* seedlings were promoted by exogenous application of MgONPs (Figure S24).

Although numerous studies have shown that MgONPs directly suppress the growth of various pathogens, such as *Ralstonia solanacearum* , *Campylobacter jejuni* , *Escherichia coli* , *Salmonella*, and *Pseudomonas aeruginosa* (He et al. 2016; Cai et al. 2018; Nguyen et al. 2018), only a few studies have reported that MgONPs induce host resistance against the bacterial pathogen *R. solanacearum* and fungal pathogen *Fusarium oxysporum* infections (Imada et al. 2016; Fujikawa et al. 2021). In particular, MgONP‐induced plant immunity against viral pathogens is unknown. We found that the application of MgONPs remarkably boosts plant resistance to TMV infection in *N. benthamiana* plants and MgONPs trigger host resistance in a dose‐dependent manner under the dynamic range of 150 μg/mL (Figure S3 and Figure 1). Previous studies showed that the application of silica nanoparticles (SiO_2_NPs) in Arabidopsis also induces SAR against *Pseudomonas syringae* in a dose‐dependent manner (El‐Shetehy et al. 2021). A higher concentration of 200 μg/mL MgONPs was less effective, and a concentration of 250 μg/mL MgONPs was ineffective in inducing host resistance, demonstrating a detrimental effect of higher concentrations of MgONPs on the induction of plant immunity (Figure S3). This is the first report that MgONPs trigger host resistance against viral pathogens in a dose‐dependent manner.

### 3.2 MgONPs Triggers Ca^2+^ Flux Through Glutamate‐Like Receptors

GLRs‐dependent Ca^2+^ flux signals play significant roles in the regulation of plant local and systemic resistance against various pathogens (Köster et al. 2022; Jiang and Ding 2023). Previous studies showed that an Arabidopsis *glr2.7/2.8/2.9* triple mutant produced using CRISPR‐Cas9 had a 25% reduction in the cytosolic calcium concentration ([Ca^2+^]_cyt_) in response to Pep1, flg22, and elf18 treatments (Bjornson et al. 2021). Interestingly, the triple mutant *glr2.7/2.8/2.9* was more susceptible to *Pseudomonas syringae* pv. tomato DC3000 infection by infiltration, which was consistent with the lower [Ca^2+^]_cyt_ response (Bjornson et al. 2021). Liu et al. (2021) identified a GLR‐encoding gene *GhGLR4.8* from *Gossypium hirsutum* , which is involved in conferring resistance to Fusarium wilt (FW) disease of cotton caused by the soil‐borne fungus, *Fusarium oxysporum* f. sp. *vasinfectum* (*Fov*). Sequence analysis suggests that *GhGLR4.8* is a homologue of the Arabidopsis *GLR3.3* gene (Liu et al. 2021; Ahmed et al. 2023). A single nucleotide substitution (C to A) in *GhGLR4.8* causes an amino acid change from leucine to isoleucine, which is responsible for resistance to Fusarium wilt in Upland cotton (Ahmed et al. 2023). Knockdown of *GhGLR4.8* using TRV‐mediated VIGS compromises cotton plant resistance to Fusarium wilt. Treatment with total secreted proteins (SEPs) of *Fov* also induced Ca^2+^ influx in the roots of cotton plants carrying the *GhGLR4.8* (Jones and Naidu 2019) allele, indicating that the *GhGLR4.8* functions in the regulation of Ca^2+^ influx (Liu et al. 2021). The study confirmed that *AtGLR3.3* and *AtGLR3.6* play an important role in plant systemic resistance to both piercing‐sucking and chewing insects (Xue et al. 2022). However, whether nanomaterials can trigger GLRs‐dependent Ca^2+^ flux to initiate plant immunity against viral disease is unknown. We thus investigated the role of MgONPs in GLRs‐dependent Ca^2+^ flux. Excitedly, this is the first report of exogenous application of MgONPs in boosting the transcript levels of numerous GLRs‐related genes in *N. benthamiana* plants by triggering the Ca^2+^ flux (Figure S7). Previous studies have shown that LaCl_3_ and DNQX function as a Ca^2+^ channel blocker and a glutamate receptor inhibitor, respectively (Cheng et al. 2018; Wang and Luan 2024). Silencing *NbGLR3.3* by VIGS and treatment with LaCl_3_ or DNQX in *GCaMP3*‐overexpressing *N. benthamiana* remarkably reduced MgONP‐triggered Ca^2+^ flux (Figure 1i–n). Several studies suggest that GLRs are involved in plant immune responses against pathogens and pests (Wudick et al. 2018). *GhGLR3.4* from cotton is involved in intracellular calcium flux. Knocking out *GhGLR3.4* by CRISPR‐Cas9 inhibits the synthesis of JA and increases cotton susceptibility to *Spodoptera litura* and *Bemisia tabaci* (Wang and Luan 2024). Genetic evidence confirms that Arabidopsis *GLR3.3* is a key component of resistance against *Hyaloperonospora arabidopsidis* (Manzoor et al. 2013). However, the role of GLRs in plant immunity against viral pathogens is rarely deciphered. In this study, we generated *Nbglr3.3* mutants through CRISPR‐Cas9. *Nbglr3.3* plants were more susceptible to TMV infection (Figure 2g–l). Application of MgONPs only partially rescued the resistance of *Nbglr3.3* mutants (Figure 2g–l). In addition, LaCl_3_ or DNQX treatments also remarkably diminished the resistance to viral pathogens (Figure 2a–f). In conclusion, MgONPs could not trigger resistance in Ca^2+^ channel‐blocked plants (Figure 2a–f).

### 3.3 MgONPs Activate Ca^2+^‐Dependent SA‐, JA‐, and ET‐Mediated Signalling Pathways

The phytohormones SA, JA, and ET play significant roles in plant immunity against diverse viruses (Zhu et al. 2014, 2025; Yang et al. 2015; Zhao and Li 2021). The concentrations of several phytohormones are regulated by NMs, such as SiNPs (Silicon nanoparticles) (Tripathi et al. 2025). Ca^2+^ influx through GLRs is involved in SA, JA, and ET biosynthesis pathways (Vidhyasekaran 2015). CaM and CDPKs recognise the changes of cytoplasmic Ca^2+^ concentration to initiate downstream signal transduction pathways (Wang and Luan 2024). Excitedly, we found that the application of MgONPs remarkably enhanced the transcript levels of *NbCaM1‐6* and *NbCDPK2* (Figure S8 and Figure S15). CaM binds to *CBP60g* or *Gα* to activate SA or JA biosynthesis (Vidhyasekaran 2015). It is well known that *CBP60g*, *Gα*, and *CDPK2* positively regulate the expression of SA, JA and ET biosynthetic genes *ICS1*, *OPR3*, and *ACCOx*, respectively (Zhang et al. 2010; Vidhyasekaran 2015). Our results showed that the application of MgONPs dramatically increased the expression of *NbCBP60g* and *NbGα*, which is consistent with the change of *NbCDPK2* (Figure S15). Meanwhile, MgONP treatments significantly increased the transcript levels of *NbICS1*, *NbOPR3*, and *NbACCOx* (Figure S16d,k,p), which enhanced the biosynthesis of SA, JA, and ET (Figure S16a–c). SA, JA, and ET signaling genes *NbNPR1*, *NbTGA2.1*, *NbTGA2.1*, *NbCOI1*, *NbMYC2*, *NbEIN2*, and *NbETR1* were also markedly up‐regulated in MgONPs‐treated plants (Figure S16e–g,l,m,q,r). Likewise, MgONP treatments remarkably enhanced the transcript levels of SA‐, JA‐, and ET‐mediated defense genes *NbPR1*, *NbPR2*, *NbPR5*, *NbPDF1.2*, *NbPR3*, and *NbPR4* (Figure S16h–j,n,o,s). Thus, our study demonstrated that MgONPs positively regulate plant immunity against viral invasion by up‐regulating SA, JA, and ET biosynthesis and downstream signaling components, which subsequently activate pathogenesis‐related proteins. Next, we confirmed that foliar application of MgONPs activates JA‐ and ET‐mediated defence signaling pathways and triggers partial resistance in *NahG*‐transgenic plants against viral pathogens (Figure 5 and Figure S18). Exogenous application of MgONPs also induced partial plant immunity against TMV infection in JA‐deficient plants generated by VIGS (Figure 6 and Figure 7). Similarly, the application of MgONPs also partially rescued resistance to viral diseases in ET‐deficient plants produced by TRV‐based VIGS (Figure 8). However, exogenous application of MgONPs did not trigger resistance to TMV in SA‐, JA‐ and ET‐deficient plants generated by TRV‐mediated VIGS in *NahG*‐transgenic *N. benthamiana* (Figure 9). Hence, SA‐, JA‐, and ET‐mediated signaling pathways are essential for MgONP‐triggered plant immunity against viral pathogens.

### 3.4 MgONPs Trigger Early ROS Bursts but Reduce ROS Accumulation at Late Stages of Viral Infections

An early ROS burst confers resistance to viral pathogens in plants (Rossetti and Bonatti 2001; Zhu et al. 2023). Excitedly, ROS staining (Figure 3a,b) and quantification (Figure 3c,d) confirmed that the application of MgONPs significantly induced the accumulation of ROS (e.g., H_2_O_2_ and O_2_˙^−^) at 12 h and 24 h treatment. Moreover, MgONP treatment at 0.5–24 h remarkably enhanced the transcript levels of numerous RBOHs, which are involved in active ROS production (Figure 3e–l). This is the first report of the application of MgONPs triggering early ROS bursts. Nevertheless, overproduction of ROS at late stages of pathogen invasion (e.g., at 2–3 dpi) often causes cell death and oxidative damage to the plants and increases susceptibility to multiple pathogens (Mittler 2002; Zhu et al. 2023). Oxidative damage induced by virus infection adversely affects the cytomembranes. Increased MDA content and electrolyte leakage are usually considered indicators of membrane lipid peroxidation, cell death, and penetrability of cytomembranes (Zhu et al. 2021). Previous studies indicate that the level of electrolyte leakage, the MDA content, and ROS accumulation were dramatically reduced in *NbLTP1*‐overexpressing plants, whereas the suppression of *NbLTP1* remarkably boosted these oxidative stress parameters during the late phases of TMV invasion (Zhu et al. 2023). In this study, ROS staining, direct measurements of ROS, and H_2_O_2_ fluorescent probe assays showed that TMV infection at late stages (at 3 dpi) markedly increased the levels of H_2_O_2_ and O_2_^•‐^, the MDA content, and the electrolyte leakage. However, exogenous application of MgONPs prominently reduced these oxidative damage parameters, including ROS accumulation (Figure S12). The reason for the MgONPs decreased ROS accumulation after TMV infection at late stages could be that MgONPs remarkably activate the activity and transcript levels of ROS‐scavenging enzymes (e.g., CAT, SOD, POD, PAL, and PPO) (Figure S13). Several studies have confirmed that an early ROS burst, followed by ROS reduction at late stages of virus infection, is essential for enhanced resistance to plant viruses (Mittler 2002; Shang et al. 2019; Zhu et al. 2021, 2023). Therefore, exogenous application of MgONPs is able to increase plant resistance by maintaining redox homeostasis and the stability of cell membranes. Overall, we confirmed that MgONPs trigger early ROS bursts but reduce ROS accumulation induced by viruses at late stages, thereby enhancing plant resistance to viral pathogens.

### 3.5 Safety Evaluation of MgONPs

The continuous release of nanomaterials into soil, water, and air has attracted rising concerns about the possible risk of nanomaterials to environmental and human health safety (Johnstona et al. 2020; Ma et al. 2023). Hence, safety assessment and evaluation of ecotoxicity are essential for risk assessment of NMs (Fadeel et al. 2018). Gao et al. assessed the ecotoxicity of the PRO‐MON‐CaC, a mesoporous organosilica nanoparticle, in zebrafish and rapeseed plants. PRO‐MON‐CaC could reduce the threats of PRO to aquatic organisms, and MON‐CaC nanocarriers exhibit desirable biosafety effects on rapeseed plants (Gao et al. 2020). Verma et al. described that MgONPs synthesised by utilising the extract of *Calotropis gigantea*, show high biocompatibility concerning morphological alterations, notochord development and heartbeat frequency in embryonic zebrafish. (Verma et al. 2020). We tested the potential ecotoxicity of MgONPs in zebrafish and mung bean plants. The phytotoxicity assessment of MgONPs confirmed that there were no significant effects on plant growth and only high concentrations (500 μg/mL) of MgONPs affected shoot and root lengths of mung bean plants (Figure S27a–e), which is consistent with previous reports (Vijayakumar et al. 2013), suggesting that MgONPs exhibit desirable biocompatibility and biosafety on mung bean plants. With in vivo safety evaluation of MgONPs in zebrafish, toxicity grades of MgONPs on zebrafish belong to lowly toxic levels (Figure S27f–i), indicating that MgONPs have satisfactory biosafety to the aquatic environment.

### 3.6 MgONPs Confer Broad‐Spectrum Viral Resistance

Various viruses often simultaneously infect vegetable crops in the process of vegetable production. TuMV, ToMV, PVY, PVX, TSWV, and TMV are well‐known agricultural threats, which can significantly reduce the yield and quality of potatoes, tobacco, oilseed rape, tomatoes, peppers, radishes, and other plants (Scholthof et al. 2011; Gao et al. 2022; Tatineni and Hein 2023). Hence, there is an urgent need to develop effective, non‐drug‐resistant, non‐toxic, sustainable, residual‐free, and eco‐friendly antiviral agents to tackle the simultaneous infection of multiple viruses. Currently, several nanomaterials have been demonstrated to increase resistance to single virus infection. Foliar exposure of Fe_3_O_4_ nanoparticles enhanced plant resistance to TMV (Cai et al. 2020). Carbon‐based nanomaterials also induced resistance against TMV in *N. benthamiana* (Adeel et al. 2021). In the present study, we provide evidence that MgONPs confer broad‐spectrum resistance against diverse viral diseases of vegetable crops, such as TuMV, ToMV, PVY, PVX, TSWV, and TMV (Figures S25 and S26).

**FIGURE 10:**
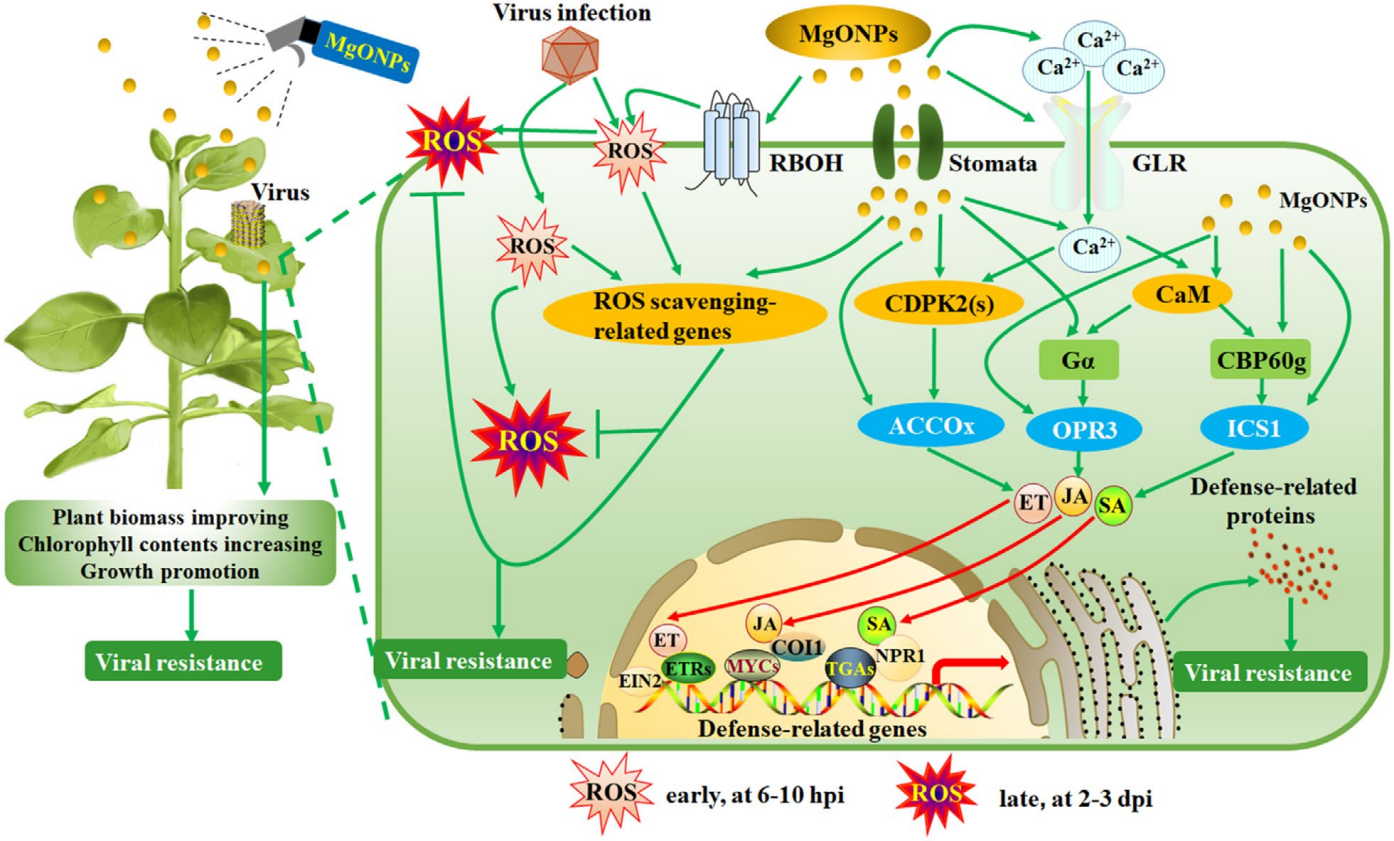
A working model of possible signalling pathways for MgONPs‐triggered plant immunity in *Nicotiana benthamiana* to viral pathogens. MgONPs trigger Ca^2+^ influx through glutamate‐like receptors, Ca^2+^ sensor proteins, NbCaMs and NbCDPKs, and the regulators of SA, JA, ET biosynthesis, *NbCBP60g*, *NbGα*, and *NbCDPK2*. This in turn stimulates SA, JA, ET biosynthesis and their downstream signalling components, which activate defence‐related proteins, consequently enhancing plant resistance to viral pathogens. MgONPs also activate *NbRBOHs* expression and trigger early ROS bursts. At the same time, MgONPs activate the activity and transcript levels of ROS‐scavenging enzymes at late phases of viral infection to maintain the redox homeostasis and the stability of cell membranes, thereby increasing plant resistance to viral pathogens. In addition, MgONPs promote plant growth, biomass, and chlorophyll contents, thus increasing plant resistance against viral pathogens.

A possible signalling pathway for MgONP‐triggered plant immunity to viral pathogens that integrates our results is summarised in Figure 10. Firstly, MgONPs remarkably boost plant resistance to TMV infection in *N. benthamiana* plants in a dose‐dependent manner. Secondly, MgONPs trigger Ca^2+^ flux through glutamate‐like receptors and Ca^2+^ sensor proteins NbCaMs and NbCDPKs. CaM binds to *CBP60g* or *Gα* to activate SA or JA biosynthesis. The expressions of SA, JA, and ET biosynthesis genes *NbICS1*, *NbOPR3*, and *NbACCOx* are up‐regulated by *NbCBP60g*, *NbGα*, and *NbCDPK2*, respectively. This in turn stimulates SA, JA, and ET biosynthesis and their downstream signalling components encoded by *NbNPR1*, *NbTGA2.1*, *NbTGA2.1*, *NbCOI1*, *NbMYC2*, *NbEIN2*, and *NbETR1*, which activate pathogenesis‐related proteins, thereby enhancing plant resistance to viruses. Thirdly, MgONPs promote *NbRBOH* expression and trigger early ROS bursts. Meanwhile, MgONPs activate the activity and transcript levels of ROS‐scavenging enzymes at late phases of viral infection to ensure redox homeostasis and to maintain cell membrane stability. Fourthly, the application of MgONPs improves plant biomass, increases chlorophyll contents and promotes growth, consequently enhancing plant performance. Safety evaluation has shown that MgONPs have desirable biocompatibility and biosafety for plants, as well as satisfactory biosafety for the aquatic environment. Finally, MgONPs confer broad‐spectrum resistance against various viral diseases in Solanaceae plants. Overall, MgONPs have great potential to be developed into plant immunity inducers and our discoveries point to a new direction for MgONPs as effective, non‐drug resistant, non‐toxic, sustainable, residual‐free, and eco‐friendly antiviral agents to simultaneously prevent diverse viral diseases.

## 4 Materials and Methods

### 4.1 Plant Materials and Growth Conditions

The wild‐type *Nicotiana benthamiana*, *NahG*‐transgenic *N. benthamiana*, *GCaMP3*‐transgenic *N. benthamiana*, *NbGLR3.3* knockout *N. benthamiana*, and *Solanum lycopersicum* wild‐type ‘Ailsa Craig’ (AC) were planted in a greenhouse at 25°C with 16‐h light/8‐h dark cycles.

### 4.2 MgO Nanoparticles, DNQX, LaCl_3_ and Characterisation of MgONPs

MgONPs (CAS No.: 1309‐48‐4) and LaCl_3_ (CAS No.: 10099‐58‐8) were provided by MACKLIN. DNQX (CAS No.: 2379‐57‐9) was purchased from MedChem Express. In all experiments, MgONPs were suspended in distilled water, and the suspension was ultrasonicated for 5 min before use. The scanning/transmission electron microscopy (SEM: S‐4800II, Hitachi, Tokyo, Japan; TEM: HT7800, Tokyo, Japan) techniques were utilised to determine the surface morphology and size of the MgONPs. To prepare samples for SEM and TEM, an aluminium stub and a carbon‐coated copper grid were utilised, respectively. The compositional fractions of MgONPs were obtained through EDS (NORAN System seven, Thermo Fisher Scientific, USA) at a voltage of 20 KeV. Surface properties, including the presence of biomolecule capping agents around the MgONPs were investigated through FTIR (Cary 610/670, Varian, USA); the particle powder was scanned in the spectral region ranging from 4000 to 500 cm^−1^. The crystalline structure of MgONPs was analysed by XRD (D8 Advance, Bruker AXS, Germany), which was conducted at a 10° to 80° 2θ diffraction angle, operating at 45 KeV of working voltage and 40 mA of current.

### 4.3 Screening Concentration and Dose Dependence of Plant Immunity

*N. benthamiana* leaves were sprayed with various concentrations of MgONPs (0, 50, 100, 150, 200, and 250 μg/mL), and then inoculated with TMV‐GFP. GFP imaging and GFP fluorescent foci quantification were used to screen for the best antiviral effect under an appropriate concentration of MgONPs. A standard log‐logistic dose–response model was employed to analyse the relationship between MgONP‐triggered viral resistance and its concentration.

### 4.4 Virus Inoculation, GFP Imaging, and GFP Fluorescent Foci Quantification

Tobacco mosaic virus (TMV) expressing green fluorescent protein (TMV‐GFP) was maintained in an aqueous suspension of 0.02 M sodium phosphate buffer (PBS, pH = 7.0) at 4°C. Potato virus Y‐GFP (PVY‐GFP), potato virus X‐GFP (PVX‐GFP), and tomato spotted wilt virus‐GFP (TSWV‐GFP) were kindly provided by Prof. Xiaorong Tao (Nanjing Agricultural University, China); turnip mosaic virus‐GFP (TuMV‐GFP) was kindly provided by Dr. Zhenggang Li (Guangdong Academy of Agricultural Sciences, China); and tomato mosaic virus‐GFP (ToMV‐GFP) was kindly provided by Dr. Peng Liu (Ningbo University, China). Virus inoculation was carried out as described previously (Manzoor et al. 2013). GFP fluorescence was imaged under ultraviolet (UV) light using a B‐100AP longwave‐UV lamp (Ultra‐Violet Products) or fluorescence stereo microscope (Manzoor et al. 2013; Zhu et al. 2023). Quantification of the GFP fluorescent foci was determined as the ratio of GFP fluorescent area to the total area, and was measured using ImageJ.

### 4.5 pKSECRISPR/Cas9‐*NbGLR3.3* Vector Construction and Generation of Transgenic Plants

To generate *NbGLR3.3* mutants using the CRISPR‐Cas9 system, the CRISPR‐P web tool (http://crispr.hzau.edu.cn/) was used to select two target sequences for *NbGLR3.3*. The scaffold containing two gRNAs was amplified by PCR using a pGTR vector as the template. PCR products were used to build the final CRISPR expression vector pKSE401 by the homologous recombination method. The reconstructed vector was introduced into the DH5α strain of *Escherichia coli* and confirmed using Sanger sequencing. The verified vector (named as pKSE401‐*NbGLR3.3*) was introduced into the *Agrobacterium tumefaciens* strain GV3101 by electroporation and transformed into *N. benthamiana*. Transgenic plants were generated by leaf disc transformation through an *Agrobacterium*‐mediated method as previously described. Briefly, the well‐cultured tobacco leaves were cultured in *Agrobacterium* culture medium carrying pKSE401‐*NbGLR3.3* for 8–10 min. Subsequently, the leaf discs were incubated on a co‐cultivation medium for 72 h at 25°C in the dark. After co‐cultivation, the leaf discs were transferred to a shoot regeneration medium and incubated in a growth chamber for 2–3 weeks until the shoots reached a length of 1–2 cm. The shoots were then separated from the callus and transferred to a rooting medium. The transgenic lines were selected on the basis of kanamycin resistance (Kan). Oligonucleotide primers used for recombinant pYLCRISPR/Cas9 vector construction are listed in Table S3.

### 4.6 Mutation Identification

The genomic DNA was extracted from freshly frozen leaves with a DNA quick Plant System Kit (Tiangen, Beijing, China). The integration of the introduced gene was confirmed by extracting genomic DNA from regenerated plants and using primers to analyze the *NptII* gene in transgenic plants by PCR. The PCR products were further verified by sequencing. Homozygous T2 lines were used in subsequent experiments.

### 4.7 Transmission Electron Microscopy (TEM) Observation of MgONPs Distribution in *N. Benthamiana* Leaves

We employed TEM to analyse the effects of MgONPs treatment on *N. benthamiana* leaves. *N. benthamiana* leaves were exposed to 150 μg/mL of MgONPs for 3 days before observation. The leaf sample collection, processing and TEM observation were performed according to the protocol described by Du et al. (2022).

### 4.8 Real‐Time Ca^2+^ Imaging and Analysis

*N. benthamiana* plants expressing the GFP‐based Ca^2+^ indicator, *GCaMP3*, were imaged with a BX53 fluorescence stereo microscope (Olympus, Japan) (Zhu et al. 2020). The green fluorescent signal in acquired images was analysed by ImageJ software (https://imagej.nih.gov/ij/).

### 4.9 Determination of Malondialdehyde (MDA) Content and Electrolyte Leakage

The MDA content was measured using a Malondialdehyde Assay Kit (Solarbio, Cat#bc0020) according to the manufacturer's protocol. Electrolyte leakage was determined as previously described (Zhu et al. 2023).

### 4.10 MgONPs‐Triggered Broad‐Spectrum Viral Resistance

*N. benthamiana* and tomato ( *S. lycopersicum* ) plants were used to investigate the role of MgONPs in broad‐spectrum viral resistance. *N. benthamiana* plants were sprayed with MgONPs, then respectively infected by TuMV‐GFP, ToMV‐GFP, PVY‐GFP, PVX‐GFP, and TSWV‐GFP. Tomato plants were pretreated with MgONPs and then respectively infected by TMV‐GFP, ToMV‐GFP, TuMV‐GFP, and PVY‐GFP. GFP fluorescent foci imaging and determination were carried out using ultraviolet (UV) light or a fluorescence stereo microscope (Zhu et al. 2023; Huang et al. 2024).

### 4.11 Planta Growth Estimation

*N. benthamiana* plants were harvested at 14 d post‐treatment with MgONPs and washed to remove adhered potting mix particles, followed by the estimation of growth characteristics, including root length, plant height, leaf area, fresh weight, dry weight, Chl*a* content, Chl*b* content, and total Chl content (Raliya et al. 2015). The *N*. b*enthamiana* samples were oven‐dried overnight at 80°C, and the dry weight was measured.

### 4.12 H_2_O_2_ and O_2_˙^−^ Visualisation and Determination

Production of O_2_˙^−^ and H_2_O_2_ was visualised using nitroblue tetrazolium (NBT) and 3,3′‐diaminobenzidine (DAB), respectively. Tobacco leaves were excised at the base with a razor blade and soaked in either 0.5 mg/mL NBT solution for 2 h or 2 mg/mL DAB solution for 8 h under a vacuum. The leaves were then destained in boiling ethanol (95%) for 15–20 min. For fluorescence microscopy, H_2_O_2_ was visualised using the H_2_O_2_ fluorescent probe 2′,7′‐dichlorofluorescein diacetate (H_2_DCF‐DA) (CAS No.: 4091‐99‐0, MedChem Express) (Deng et al. 2016). Leaf fragments were pre‐loaded with 50 μM H_2_DCF‐DA in Tris/KCl loading buffer (10 mM Tris and 50 mM KCl, pH 7.2) for 10 min in darkness at 25°C. After that, the leaves were washed three times using Tris/KCl loading buffer for 5 min each at 25°C. GFP fluorescence was visualised using a BX53 fluorescence stereo microscope. O_2_˙^−^ and H_2_O_2_ concentrations were determined using a Micro Superoxide Anion Assay Kit (Solarbio, Cat#bc1290) and a Hydrogen Peroxide Assay Kit (Solarbio, Cat#bc3590), respectively.

### 4.13 Determination of Defence‐Related Enzyme Activities and SA, JA, ET Measurement

The activities of catalase (CAT), polyphenol oxidase (PPO), peroxidase (POD), superoxide dismutase (SOD), and phenylalanine ammonialyase (PAL) were investigated in 150 mg/mL MgONPs‐treated *N. benthamiana* plants in a 72‐h time course (0, 6, 12, 24, 48, and 72 h) after TMV‐GFP inoculation. *N. benthamiana* leaves were collected at different time points (0, 6, 12, 24, 48, and 72 h) after TMV‐GFP infection and frozen in liquid nitrogen immediately. Each frozen sample was treated as described previously (Ji et al. 2020). Finally, the activities of CAT, PPO, POD, SOD, and PAL were determined by assay kits (Solarbio, Beijing, China) according to the manufacturer's instructions. The contents of SA, JA, and ET were measured according to previous methods (Zhu et al. 2024). The SA, JA, and ET were extracted as described previously (Pan et al. 2010; Zhu et al. 2024). Finally, the total SA, JA, and ET contents were measured using Plant SA ElISA Kit (Shanghai Qiaodu, CAS No.: YX‐190100P), Plant JA ElISA Kit (Shanghai Qiaodu, CAS No.: YX‐100100P), and Plant ETH ElISA Kit (Shanghai Qiaodu, CAS No.: YX‐052008P) according to the manufacturer's instructions.

### 4.14 Construction of VIGS Vector and TRV‐Mediated VIGS Assays

The construction of the *NbGLR3.3‐*, *NbRbohA‐*, *NbRbohB‐*, *NbOPR3‐*, *NbCOI1‐*, *NbACCOx‐* and *NbEIN2*‐silencing VIGS vectors and the TRV‐mediated VIGS assay were performed according to the protocol described by Zhu et al. (Zhu et al. 2021; Noman et al. 2024). Partial cDNA of these target genes was amplified by RT‐PCR. Firstly, the PCR products of these target genes were cloned into the pCR8/GW/TOPO vector (Invitrogen, Cat#K250020). Then, partial fragments of target genes were inserted into the TRV vector (pTRV‐RNA2) using Gateway LR Clonase (Invitrogen, Cat#11791020). TRV‐mediated VIGS assays were performed according to the protocol described by Zhu et al. (2023). The TRV‐*GUS* vector (kindly provided by Prof. Kai Xu, Nanjing Normal University, China) was used as the control. The gene‐specific primer sequences for constructing the VIGS vector (*NbGLR3.3*‐*VIGS*, *NbRbohA*‐*VIGS*, *NbRbohB*‐*VIGS*, *NbOPR3*‐*VIGS*, *NbCOI1*‐*VIGS*, *NbACCOx*‐*VIGS*, and *NbEIN2*‐*VIGS*) are listed in Table S2.

### 4.15 RT‐PCR and Quantitative Real‐Time PCR (RT‐qPCR) Analysis

RT‐PCR was used to construct the VIGS silencing vectors. Total RNAs were isolated with FreeZol Reagent (Vazyme, Cat#R711) and 5 μg of RNA was reverse‐transcribed into cDNA with HiScript III 1st Strand cDNA Synthesis Kit (Vazyme, Cat#R312) according to the manufacturer's protocol. RT‐qPCR analysis was performed in a BIORAD qPCR instrument using ChamQ SYBR qPCR Master Mix (Vazyme, Cat#Q311) according to the manufacturer's instructions. Primers used in RT‐qPCR and RT‐PCR in this study are listed in Table S1. *ACTIN* was used as an internal control for normalisation in RT‐qPCR, and data were analysed by Prism8 software (Graphpad) (Zhu et al. 2023).

### 4.16 Protein Extraction and Western Blot Assay

Total proteins were extracted from fresh leaf samples using the extraction buffer (50 mM Tris‐Cl, pH 6.8, 10% glycerol, 5% mercaptoethanol, 4 M urea, 4% SDS) in an ice bath. Western blot assays were performed as previously described (Zhu et al. 2023).

### 4.17 In Vivo Toxicity of MgONPs to Zebrafish

Zebrafish ( *Danio rerio* ) were purchased from Haowangjiao Aquarium (Yangzhou, Jiangsu Province, China) as the in vivo model to assess the toxicity of MgONPs. Before exposure, adult zebrafish were acclimated in an aquaculture system at 28°C ± 0.5°C with a 12 h/12 h light/dark cycle and oxygen saturation greater than 70%. After acclimatisation, fish were exposed to MgONPs at different concentrations of 0, 0.5, 1, 1.5, and 2 mg/mL for 4 days. All fish were unfed during the exposure period. Zebrafish deaths were recorded daily to evaluate the acute toxicity of the MgONPs.

### 4.18 Safety Assessment of MgONPs in Mung Bean Plants

The phytotoxicity of MgONPs was assessed with mung bean ( *Vigna radiata* L.). Before exposure, the seed surface was disinfected with NaOCl (1.2%) washing solution. Sterilized seeds were placed on plates with 50, 150, 250, and 500 mg/mL of MgONPs for 96 h. Sterile water was used as the negative control. Seed germination was assessed after 18 h of treatment with MgONPs. Shoot and root lengths and fresh weight were determined after 96 h of treatment with MgONPs.

### 4.19 Quantification and Statistical Analysis

The values are presented as mean ± standard deviation of at least three biological replicates, each with three technical repeats. The statistical significance of differences was analysed by a two‐tailed Student's *t*‐test between two groups (*p < 0.05, **p < 0.01, ***p < 0.001, ****p < 0.0001) and by one‐way ANOVA followed by Tukey's test between multiple groups (Different letters indicate significant differences, *p* < 0.05). For quantification analysis, the intensities of fluorescence were quantified using the ImageJ software (https://imagej.nih.gov/ij/).

## Author Contributions

F.Z. conceived the study. F.Z. designed experiments and wrote the manuscript. X.‐W.W., L.L., and K.‐Z.Z. performed experiments. X.‐W.W., Z.J., X.‐R.C., and F.Z. analysed data. F.Z., and J.W. reviewed and edited the manuscript.

## Conflicts of Interest

The authors declare no conflicts of interest.

## Supporting information


**Figure S1:** Physico‐morphological properties of MgONPs.
**Figure S2:** Phenotype of *Nicotiana benthamiana* under white light after 3 days of foliar treatment of ddH_2_O or different concentrations of MgONPs.
**Figure S3:** Screening of concentrations and MgONPs trigger dose‐dependent plant immunity.
**Figure S4:** The phenotype of *N. benthamiana* plants treated with 150 μg/mL MgONPs or ddH_2_O for 3 days.
**Figure S5:** TEM of MgONPs distribution in *N. benthamiana* leaves.
**Figure S6:** Silencing the expression of *NbGLR3.3* in *N. benthamiana* plants by TRV‐mediated virus‐induced gene silencing (VIGS).
**Figure S7:** MgONPs induce the expression of *NbGLRs*.
**Figure S8:** MgONPs trigger the expression of Ca^2+^ sensor genes.
**Figure S9:** Phenotype of *N. benthamiana* plants treated with ddH_2_O, MgONPs, LaCl_3_ + ddH_2_O, LaCl_3_ + MgONPs, DNQX + ddH_2_O, or DNQX + MgONPs.
**Figure S10:** CRISPR‐Cas9‐mediated targeted mutation of *NbGLR3.3*.
**Figure S11:** Phenotype of WT and *Nbglr3.3* mutants (∆12 and ∆20) treated with 150 μg/mL MgONPs or ddH_2_O for 3 days.
**Figure S12:** MgONPs reduce oxidative damage and the accumulation of ROS after TMV infection at late stages.
**Figure S13:** MgONPs induce the activities of antioxidant enzymes and the expression of ROS‐scavenging enzyme genes.
**Figure S14:** Silencing of *NbRbohA* and *NbRbohB* in *N. benthamiana* plants by TRV‐mediated VIGS.
**Figure S15:** MgONPs induce the expression of Ca^2+^ downstream‐related genes in *N. benthamiana*.
**Figure S16:** MgONPs treatment activates the SA, JA and ET‐mediated signalling pathways.
**Figure S17:** Phenotype of *NahG‐*transgenic plants treated with 150 μg/mL MgONPs or ddH_2_O for 3 days.
**Figure S18:** MgONPs reduce TMV‐induced ROS and activate JA‐ and ET‐mediated defence pathways in *NahG‐*transgenic plants.
**Figure S19:** Silencing *NbOPR3* in *N. benthamiana* plants by TRV‐mediated VIGS.
**Figure S20:** Silencing *NbCOI1* in *N. benthamiana* plants by TRV‐mediated VIGS.
**Figure S21:** Silencing *NbACCOx* in *N. benthamiana* plants by TRV‐mediated VIGS.
**Figure S22:** Silencing *NbEIN2* in *N. benthamiana* plants by TRV‐mediated VIGS.
**Figure S23:** Simultaneously silencing *NbOPR3* and *NbACCOx* in *NahG*‐transgenic plants by TRV‐mediated VIGS.
**Figure S24:** MgONPs promote plant growth.
**Figure S25:** Foliar application of MgONPs induces broad‐spectrum resistance of *N. benthamiana* to major viral diseases of vegetable crops.
**Figure S26:** MgONPs induce broad‐spectrum resistance of *S. lycopersicum* to major viral diseases of vegetable crops.
**Figure S27:** Safety evaluation of MgONPs in mung bean plants and zebrafish.
**Table S1:** Primer sequences used for quantitative real‐time PCR analysis of gene expression.
**Table S2:** Primer sequences used for semiquantitative RT‐PCR analysis of vector construction for VIGS.
**Table S3:** Primer sequences used for semiquantitative RT‐PCR analysis of *NbGLR3.3* vector construction for CRISPR and knockout validation.

## Data Availability

The data that supports the findings of this study are available in the Supporting Information of this article.
