# Supplementary material for: Join the Green and Sustainable Team: Magnesium Oxide Nanoparticles Boost Broad‐Spectrum Viral Resistance in Solanaceae Plants
Source: Plant Biotechnol J. 2025 Nov 14;24(4):1966–91. doi: 10.1111/pbi.70461 (PMC13140349; doi:10.1111/pbi.70461)
Supplement: Supplementary file 1 — Figure S1: Physico‐morphological properties of MgONPs. Figure S2: Phenotype of Nicotiana benthamiana under white light after 3 days of foliar treatment of ddH2O or different concentrations of MgONPs. Figure S3: Screening of concentrations and MgONPs trigger dose‐dependent plant immunity. Figure S4: The phenotype of N. benthamiana plants treated with 150 μg/mL MgONPs or ddH2O for 3 days. Figure S5: TEM of MgONPs distribution in N. benthamiana leaves. Figure S6: Silencing the expression of NbGLR3.3 in N. benthamiana plants by TRV‐mediated virus‐induced gene silencing (VIGS). Figure S7: MgONPs induce the expression of NbGLRs. Figure S8: MgONPs trigger the expression of Ca2+ sensor genes. Figure S9: Phenotype of N. benthamiana plants treated with ddH2O, MgONPs, LaCl3 + ddH2O, LaCl3 + MgONPs, DNQX + ddH2O, or DNQX + MgONPs. Figure S10: CRISPR‐Cas9‐mediated targeted mutation of NbGLR3.3. Figure S11: Phenotype of WT and Nbglr3.3 mutants (∆12 and ∆20) treated with 150 μg/mL MgONPs or ddH2O for 3 days. Figure S12: MgONPs reduce oxidative damage and the accumulation of ROS after TMV infection at late stages. Figure S13: MgONPs induce the activities of antioxidant enzymes and the expression of ROS‐scavenging enzyme genes. Figure S14: Silencing of NbRbohA and NbRbohB in N. benthamiana plants by TRV‐mediated VIGS. Figure S15: MgONPs induce the expression of Ca2+ downstream‐related genes in N. benthamiana. Figure S16: MgONPs treatment activates the SA, JA and ET‐mediated signalling pathways. Figure S17: Phenotype of NahG‐transgenic plants treated with 150 μg/mL MgONPs or ddH2O for 3 days. Figure S18: MgONPs reduce TMV‐induced ROS and activate JA‐ and ET‐mediated defence pathways in NahG‐transgenic plants. Figure S19: Silencing NbOPR3 in N. benthamiana plants by TRV‐mediated VIGS. Figure S20: Silencing NbCOI1 in N. benthamiana plants by TRV‐mediated VIGS. Figure S21: Silencing NbACCOx in N. benthamiana plants by TRV‐mediated VIGS. Figure S22: Silencing NbEIN2 in N. benthamiana pla [file PBI-24-1966-s001.docx]

**Supporting information**


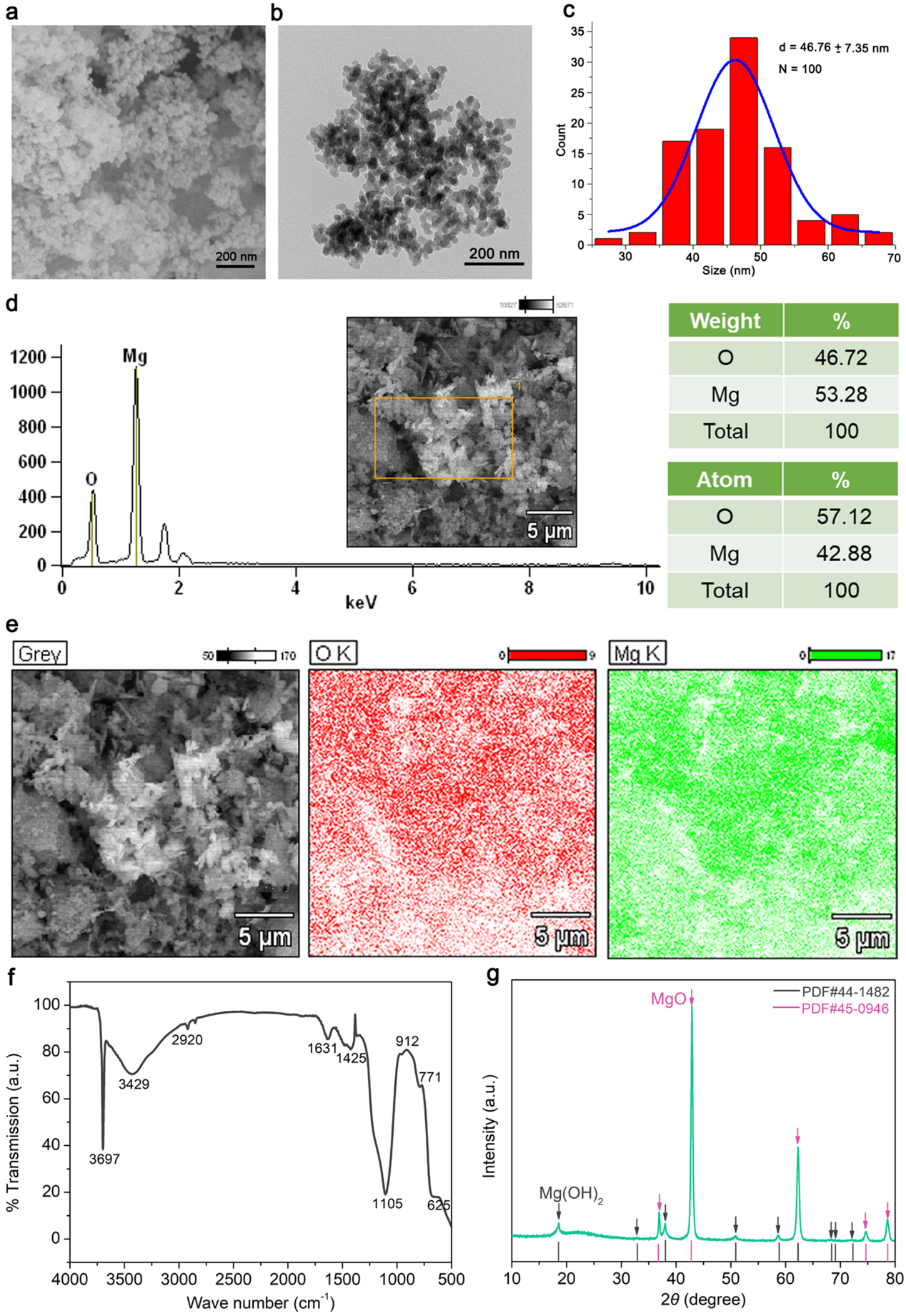


**Figure S1 Physico-morphological properties of MgONPs. a,b,** SEM images (**a**) and TEM images (**b**) showing the morphology of MgONPs. **c,** Size distribution of MgONPs calculated from SEM micrographs (**b**). **d,e,** EDS spectra (**d**) and elemental mapping (**e**) showing chemical composition and fractions of MgONPs. The most abundant elements are displayed by fluorescence: O - red; Mg - green. **f**, FTIR spectra indicating the presence of different functional groups around the surface of MgONPs. **g**, XRD spectra revealing the crystalline phase of MgONPs.

**
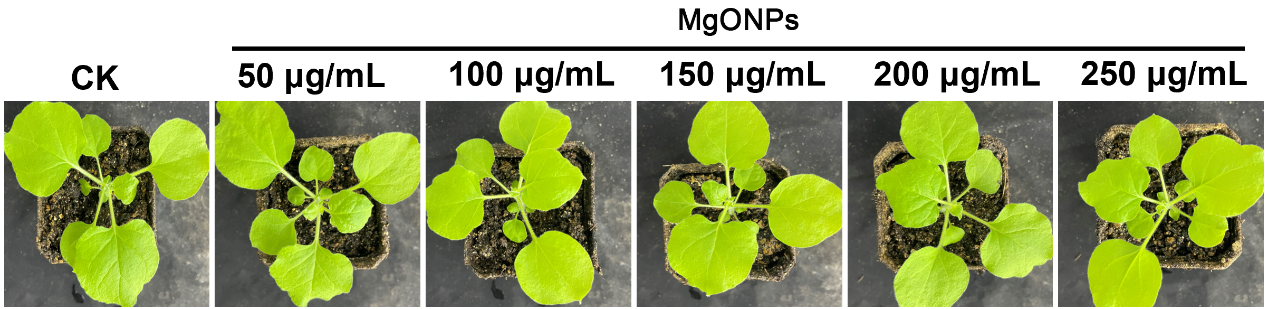
**

**Figure S2 Phenotype of *Nicotiana benthamiana* under white light after three days of foliar treatment of ddH_2_O or different concentrations of MgONPs.**

**
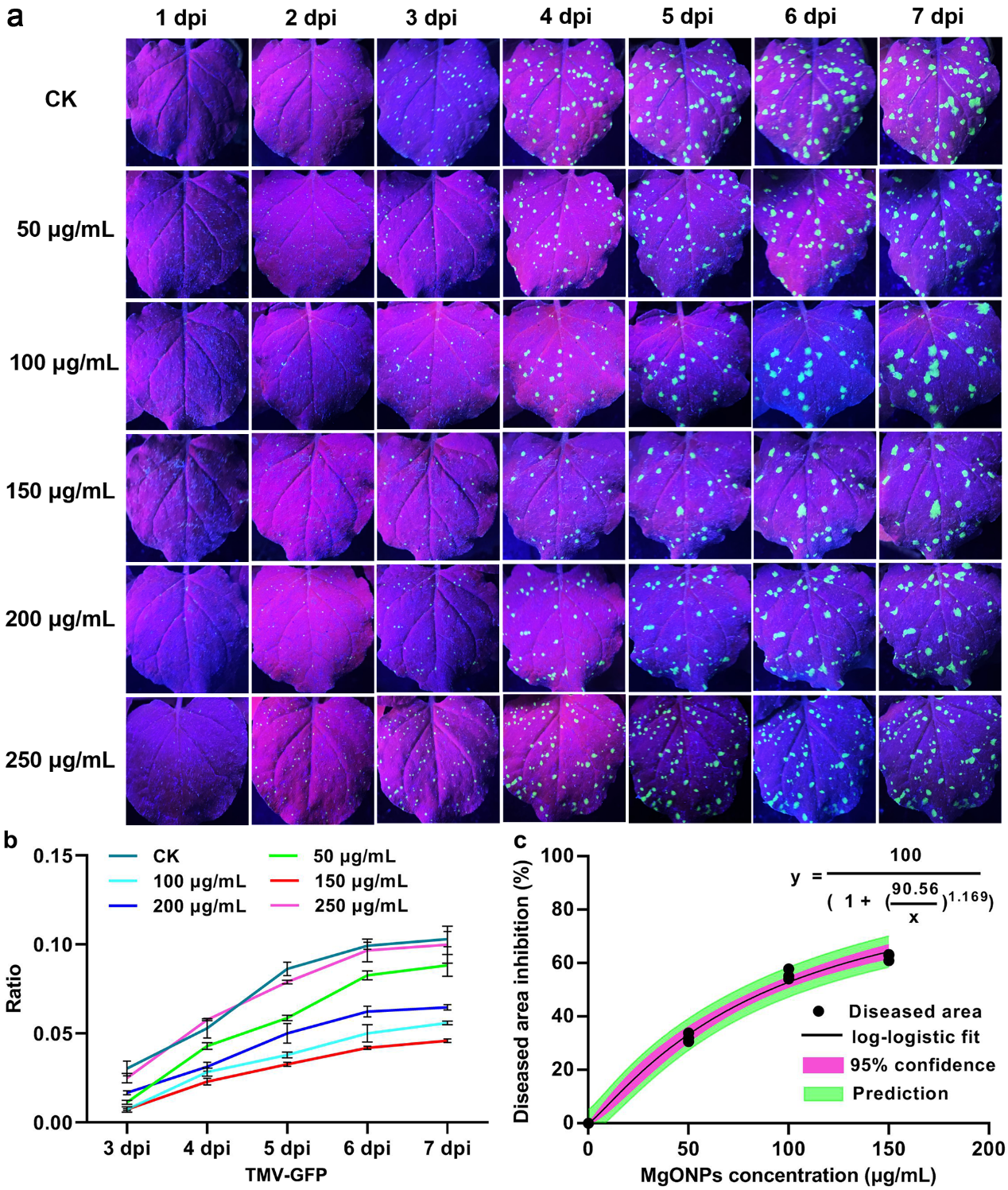
**

**Figure S3 Screening of concentrations and MgONPs trigger dose-dependent plant immunity.** **a**, Representative images of GFP fluorescence visualized in the inoculated leaves of different concentrations of MgONPs-treated plants and water-treated plants (CK) at different time-points after infection with TMV-GFP. **b**, The ratio of GFP fluorescence area to the total area of the inoculated leaves of MgONPs-treated plants and water-treated plants (CK) shown in **a**. **c**, A standard log-logistic dose–response model. MgONPs-triggered dose-dependent viral inhibition at 7 d after infection in wild-type *N. benthamiana* with TMV-GFP.

**
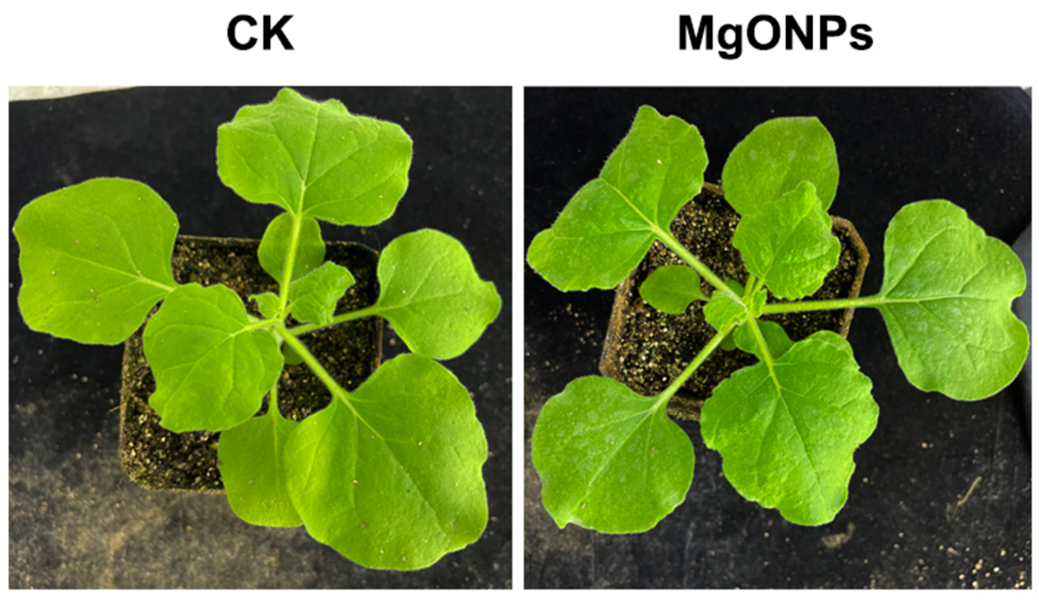
**

**Figure S4 The phenotype of *N. benthamiana* plants treated with 150 μg/mL MgONPs or ddH_2_O for 3 days.**


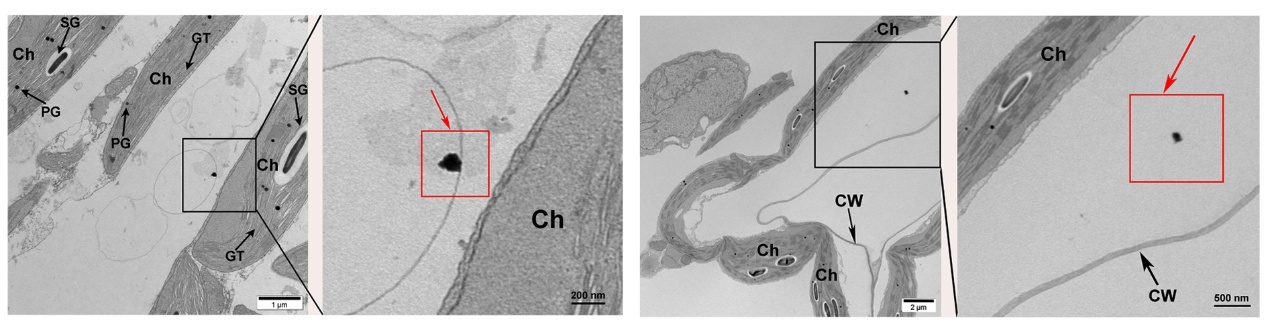


**Figure S5** **TEM of MgONPs distribution in *N. benthamiana* leaves.** TEM observation of MgONPs distribution in *N. benthamiana* leaves under foliar treatment. Leaves from 4-5-week-old *N. benthamiana* plants were exposed to 150 μg/mL of MgONPs for 3 days before observation. Red arrows point to the nanoparticles. The boxes with solid lines represent the magnification of the part.

**
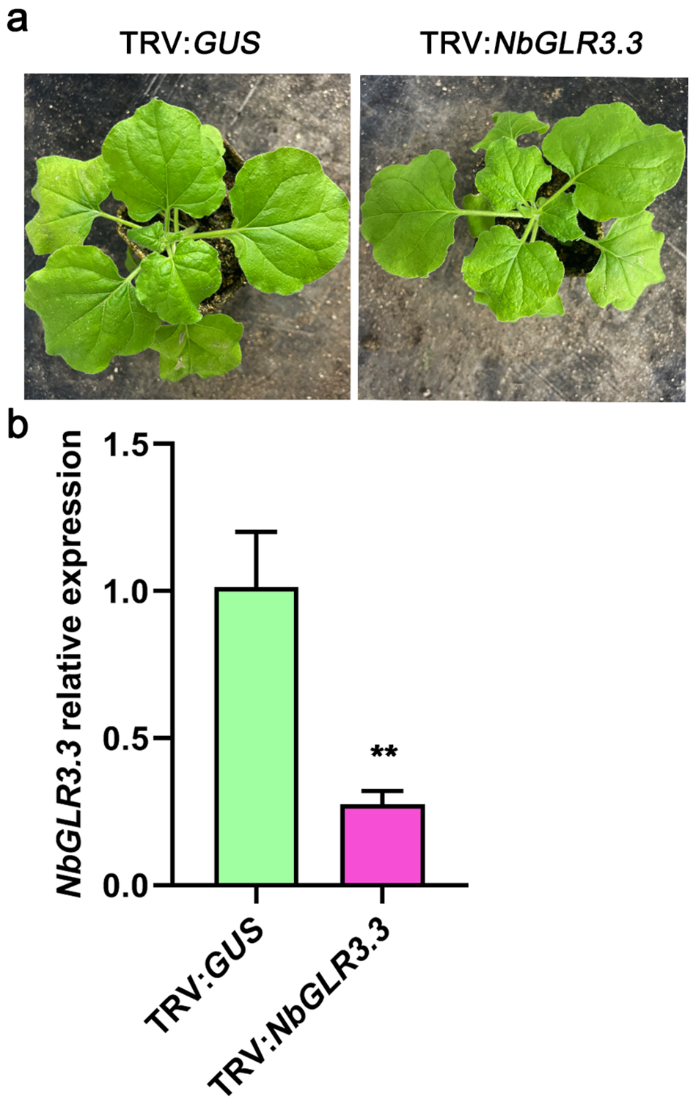
**

**Figure S6** **Silencing the expression of *NbGLR3.3* in *N. benthamiana* plants by TRV-mediated virus induced gene-silencing (VIGS).** (a) Phenotype of *NbGLR3.3*-silenced *N. benthamiana* and control plants (TRV:*GUS*). (b) RT-qPCR analysis of the expression levels of *NbGLR3.3* in *NbGLR3.3*-silenced *N. benthamiana* plants. *ACTIN* was used as the internal reference gene, and the expression is relative to that in the control plants (TRV:*GUS*), the value of which was set as 1. Bars represent mean and standard deviation of values obtained from three biological replicates. Asterisks represent significant difference determined by Student’s *t* test (** *P* < 0.01).

**
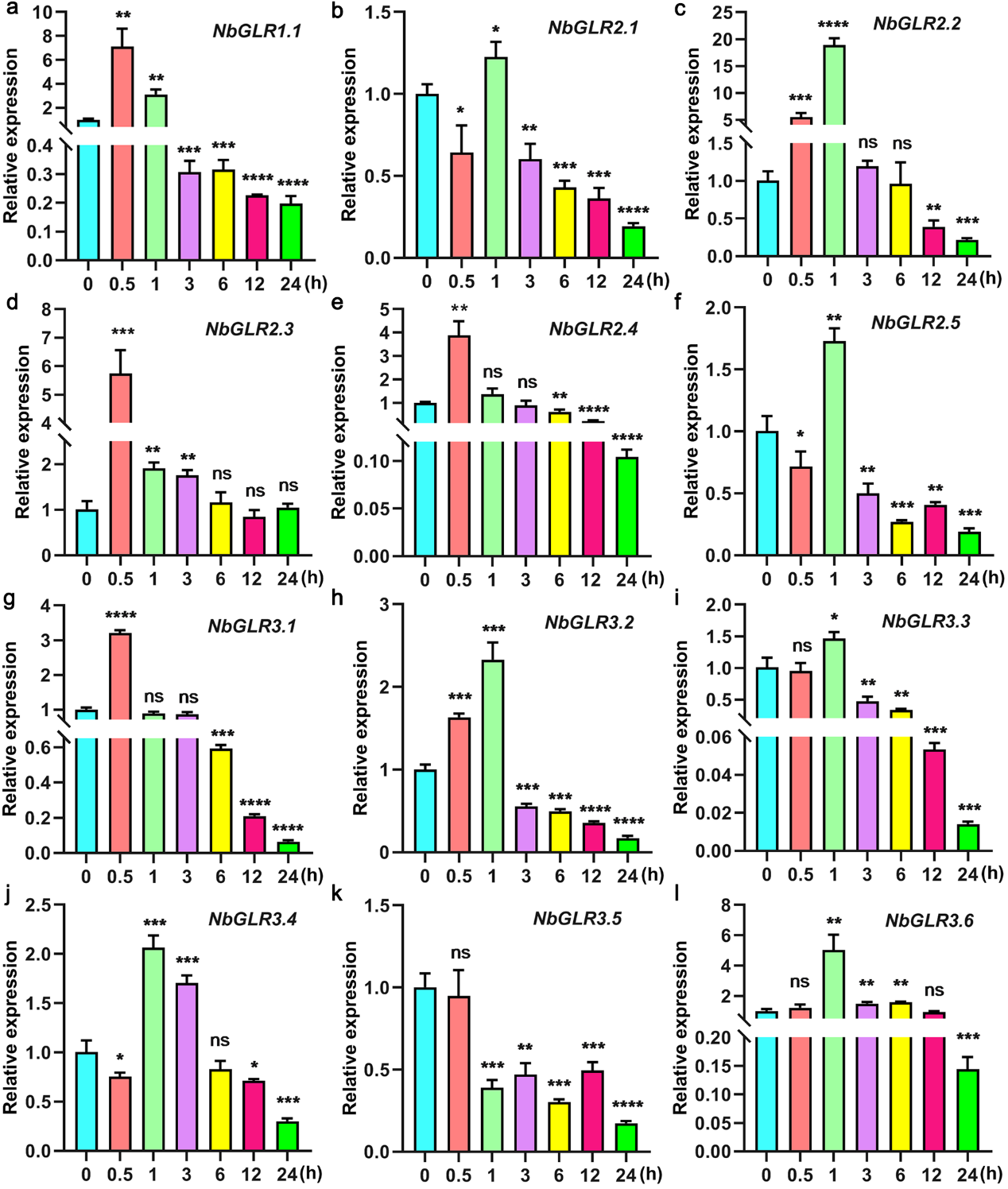
**

**Figure S7** **MgONPs induce the expression of *NbGLRs*. a-l,** RT-qPCR analysis of the expression of the glutamate receptor genes *NbGLR1.1*(**a**), *NbGLR2.1*(**b**), *NbGLR2.2*(**c**), *NbGLR2.3*(**d**), *NbGLR2.4*(**e**), *NbGLR2.5*(**f**), *NbGLR3.1*(**g**), *NbGLR3.2*(**h**), *NbGLR3.3*(**i**), *NbGLR3.4*(**j**), *NbGLR3.5*(**k**) and *NbGLR3.6*(**l**) in *N. benthamiana* plants treated with MgONPs at different time points. *ACTIN* was used as the internal reference gene, and the expression is relative to that in the ddH_2_O treatment control (0 h), the value of which was set as 1. All data are means ± SD, *n*=3. Significant differences compared with the 0 h were determined using Student’s *t*-test: **P*<0.05, ***P*<0.01, ****P*<0.001, *****P*<0.0001.

**
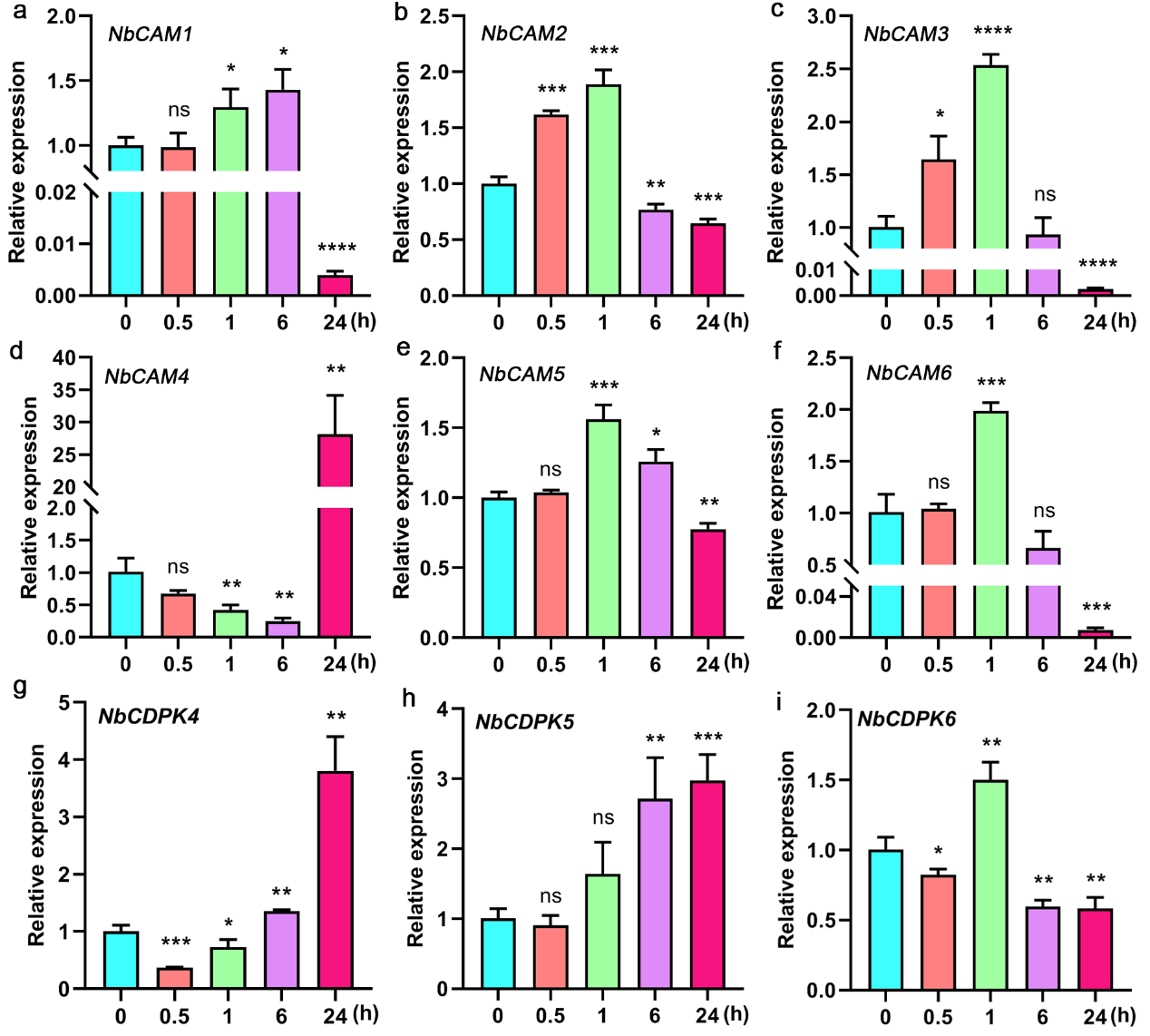
**

**Figure S8** **MgONPs trigger the expression of Ca^2+^ sensor genes. a-i**, RT-qPCR analysis of the expression of the *NbCAMs* (**a-f**) and *NbCDPKs* (**g-i**) in *N. benthamiana* plants treated with MgONPs at different time points. *ACTIN* was used as the internal reference gene, and the expression is relative to that in the ddH_2_O treatment control (0 h), the value of which was set as 1. All data are means ± SD, *n*=3. Significant differences compared with the 0 h were determined using Student’s *t*-test: **P*<0.05, ***P*<0.01, ****P*<0.001, *****P*<0.0001.

**
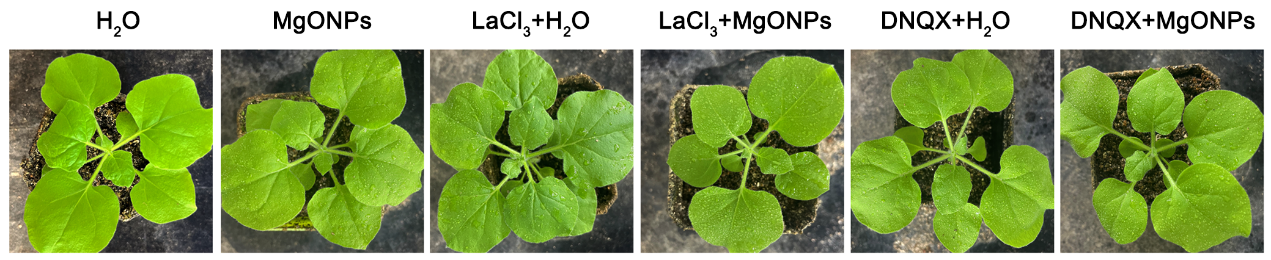
**

**Figure S9** **Phenotype of *N. benthamiana* plants treated with ddH_2_O, MgONPs, LaCl_3_ + ddH_2_O, LaCl_3_ + MgONPs, DNQX + ddH_2_O, or DNQX + MgONPs.**

**
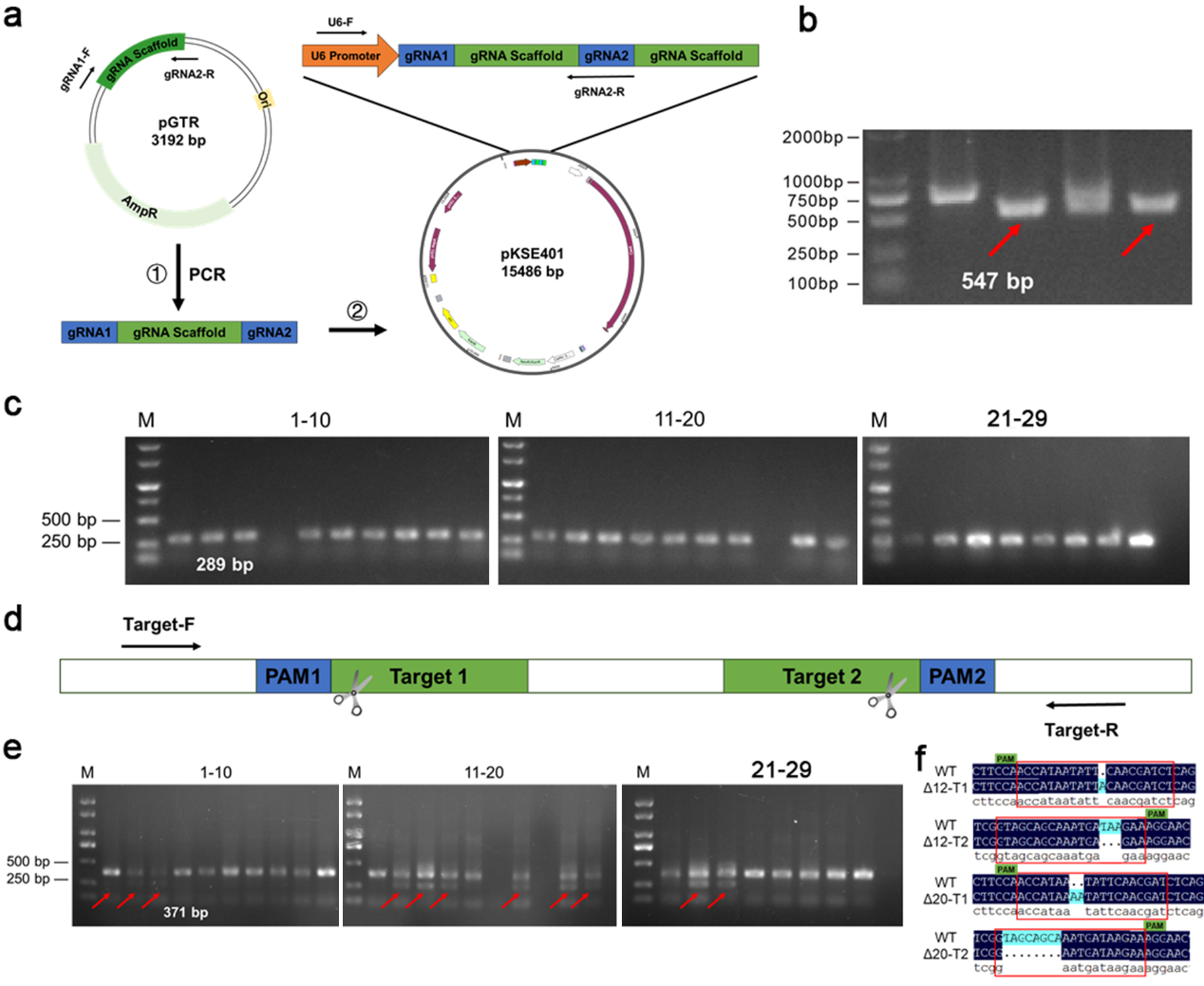
**

**Figure S10** **CRISPR-Cas9-mediated targeted mutation of *NbGLR3.3.*** (a) The CRISPR-Cas9 vector structure used for targeted mutation of *NbGLR3.3*. (b) Positive clones were screened by PCR. (c) PCR identification of positive transformation lines. (d) Targeting sites and gRNA sequences used for *NbGLR3.3* genes editing. The three base protospacer adjacent motif (PAM) was in blue. (e) PCR-based genotyping results for the *Cas9* gene in the positive mutant lines. (f) Nucleotide sequence alignment of *NbGLR3.3* in pKSE401-*NbGLR3.3* transformants. Target sites of *NbGLR3.3* are represented by boxes. Insertions and deletions are shown with marked differences in the sequences, with hyphens representing deletions.

**
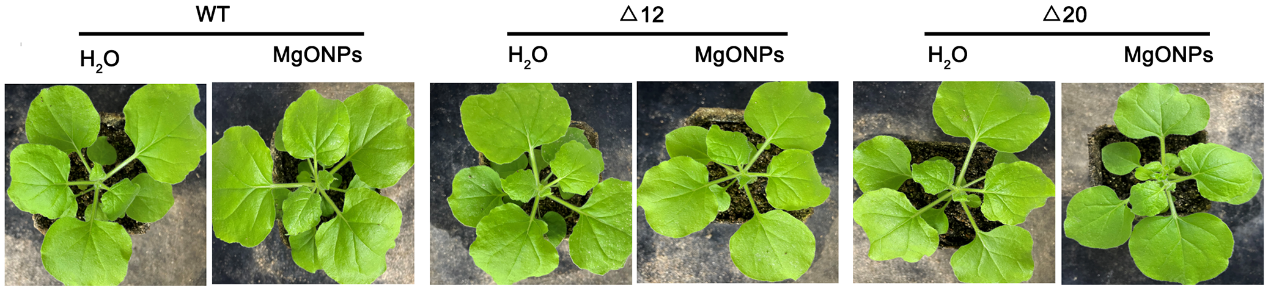
**

**Figure S11** **Phenotype of WT and *Nbglr3.3* mutants (∆12 and ∆20) treated with 150 μg/mL MgONPs or ddH_2_O for 3 days.**

**
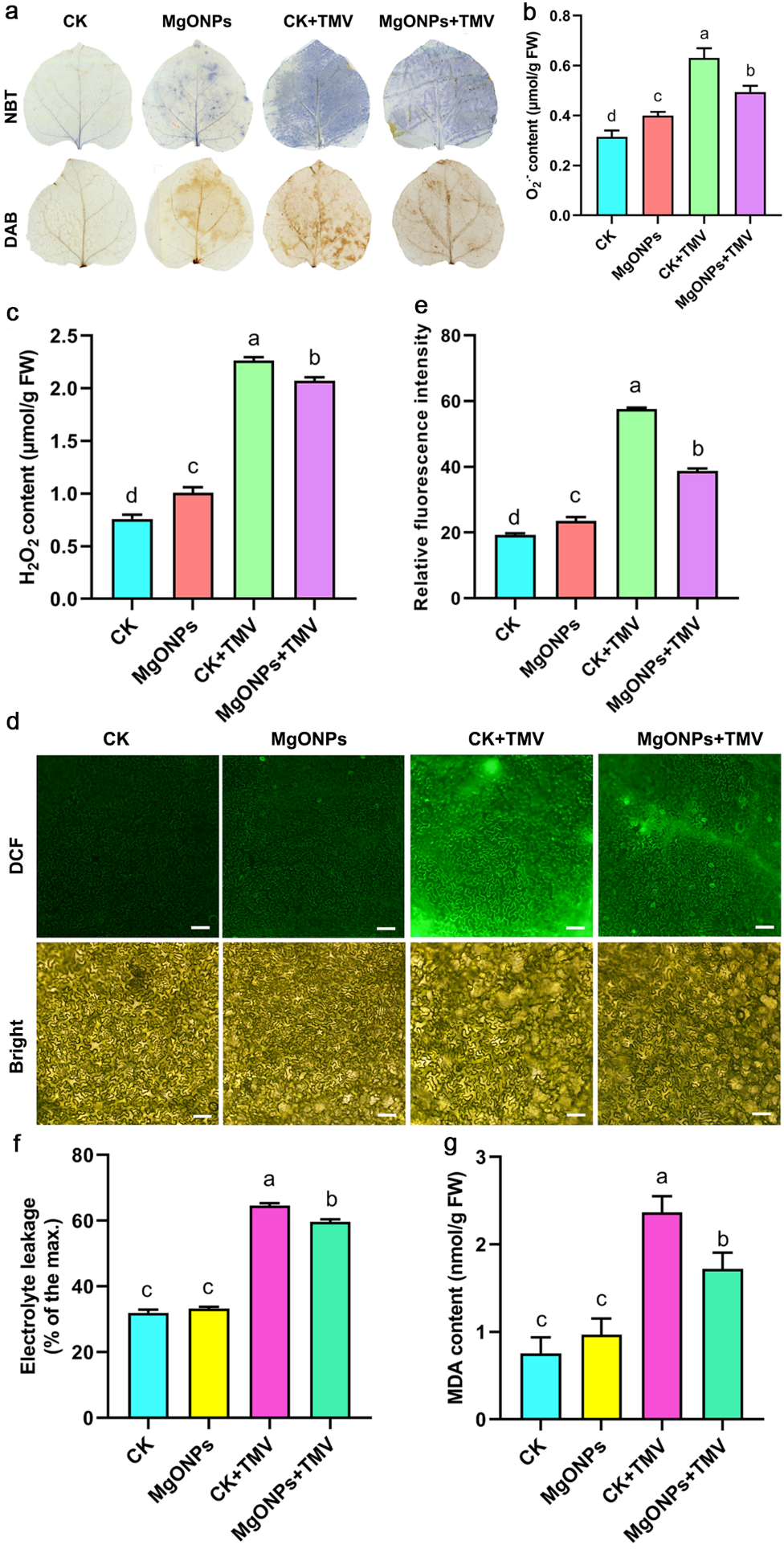
**

**Figure S12** **MgONPs reduce oxidative damage and the accumulation of ROS after TMV infection at late stages. a**, The levels of H_2_O_2_ and O_2_^•-^ were visualized by DAB and NBT staining, respectively, in MgONPs- or water-treated leaves at 3 dpi with TMV-GFP. **b,c**, The O_2_^•-^ (**b**) and H_2_O_2_ (**c**) contents were measured in MgONPs- or water-treated leaves at 3 dpi after infection with TMV-GFP by a Superoxide Anion Activity Content Assay Kit and a Hydrogen Peroxide Assay Kit, respectively. **d**, H_2_O_2_ levels in MgONPs- or water-treated leaves at 3 dpi with TMV-GFP stained with H_2_DCF fluorescent probe were detected by Fluorescence microscope. Scale bar, 100 μm. **e**, Relative fluorescence intensity of H_2_O_2_ signals of the leaves shown in **d**. **f,g**, Electrolyte leakage (**f**) and MDA contents (**g**) measured in the leaves of MgONPs- or water-treated plants at 3 dpi with TMV-GFP. Different letters indicate statistically significant differences among the means after one-way ANOVA analysis (*P*<0.05).

**
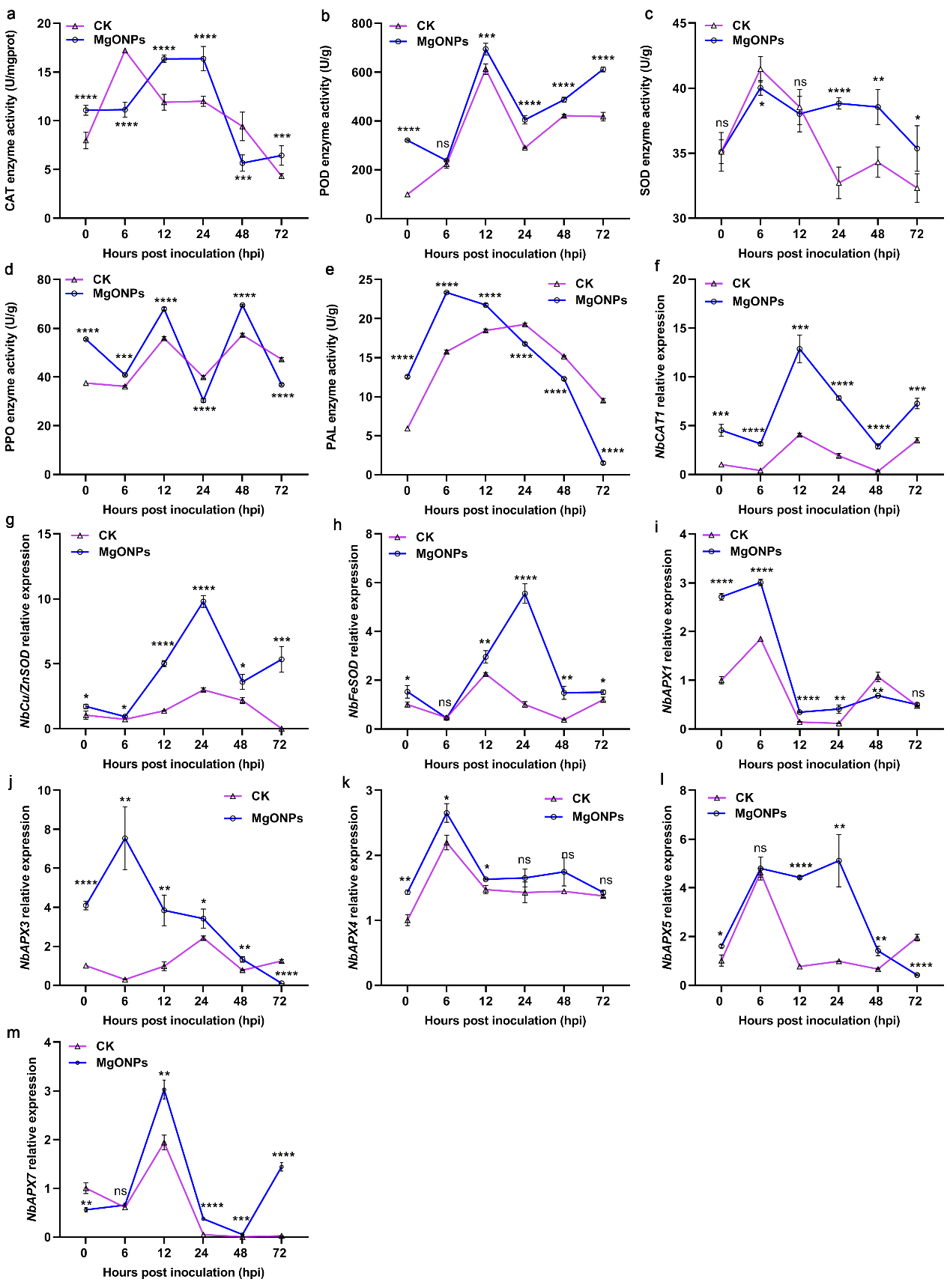
**

**Figure S13** **MgONPs induce the activities of antioxidant enzymes and the expression of ROS-scavenging enzyme genes. a-e,** The activities of antioxidant enzymes CAT (**a**), POD (**b**), SOD (**c**) and PPO (**d**) PAL (**e**) in the *N. benthamiana* plants under MgONPs or water treatments at different time points after infection with TMV-GFP. **f-m**, RT-qPCR analysis of the expression of ROS-scavenging enzyme genes in *N. benthamiana* plants under MgONPs or water treatments at different time points after infection with TMV-GFP. *ACTIN* was used as the internal reference gene, and the expression is relative to that in the WT with ddH_2_O treatment, the value of which was set as 1. Asterisks represent signiﬁcant differences determined by Student’s t-test (**P* < 0.05; ***P* < 0.01; ****P* < 0.001; *****P* < 0.0001).

**
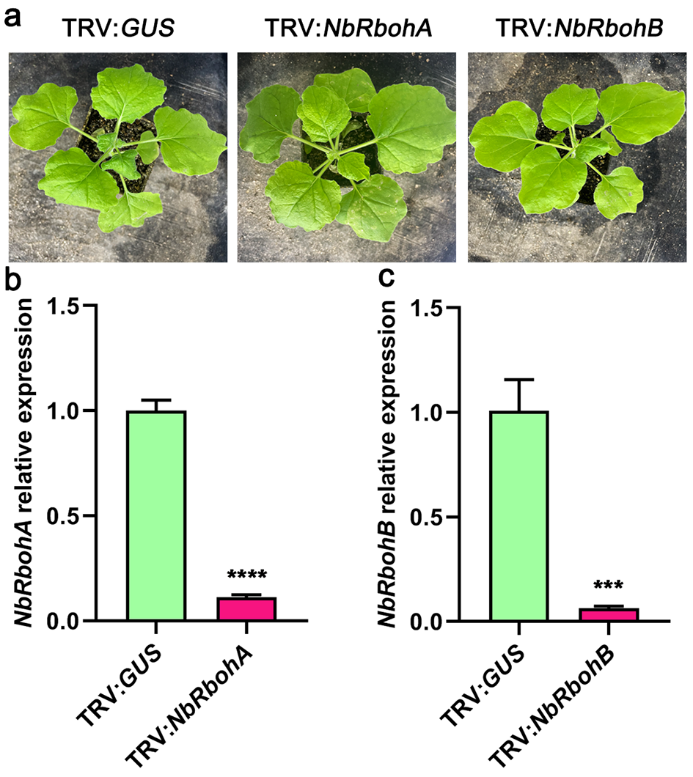
**

**Figure S14** **Silencing of *NbRbohA* and *NbRbohB* in *N. benthamiana* plants by TRV-mediated VIGS.** (a) Phenotype of *NbRbohA*- or *NbRbohB*-silenced *N. benthamiana* and control plants (TRV:*GUS*). (b) RT-qPCR analysis of the expression levels of *NbRbohA* in *NbRbohA*-silenced *N. benthamiana* plants. *ACTIN* was used as the internal reference gene, and the expression is relative to that in the control plants (TRV:*GUS*), the value of which was set as 1. (c) RT-qPCR analysis of the expression levels of *NbRbohB* in *NbRbohB*-silenced *N. benthamiana* plants. *ACTIN* was used as the internal reference gene, and the expression is relative to that in the control plants (TRV:*GUS*), the value of which was set as 1. Bars represent mean and standard deviation of values obtained from three biological replicates. Asterisks represent significant difference determined by Student’s t test (*** *P* < 0.001, **** *P* < 0.0001).

**
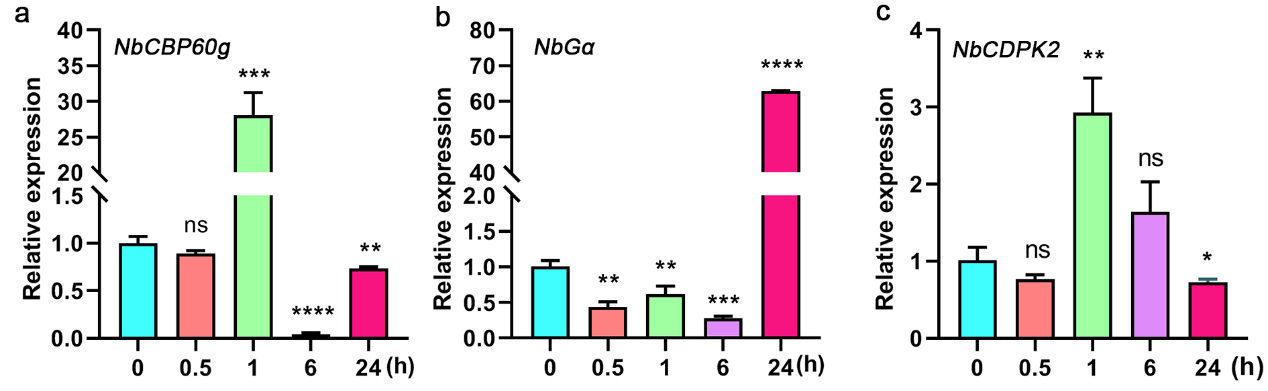
**

**Figure S15** **MgONPs induce the expression of Ca^2+^ downstream-related genes in *N. benthamiana*.** RT-qPCR analysis of the expression of calmodulin (CaM)-binding protein *NbCBP60g* (a), guanosine triphosphate (GTP)-binding protein *NbGα* (b), and Ca^2+^-dependent protein kinase *NbCDPK2* (c) in *N. benthamiana* plants treated with MgONPs at different time points. *ACTIN* was used as the internal reference gene, and the expression is relative to that in the WT with ddH_2_O treatment, the value of which was set as 1. Bars represent mean and standard deviation of values obtained from three biological replicates. Asterisks represent significant difference determined by Student’s t test (* *P* < 0.05, ** *P* < 0.01, *** *P* < 0.001, **** *P* < 0.0001).


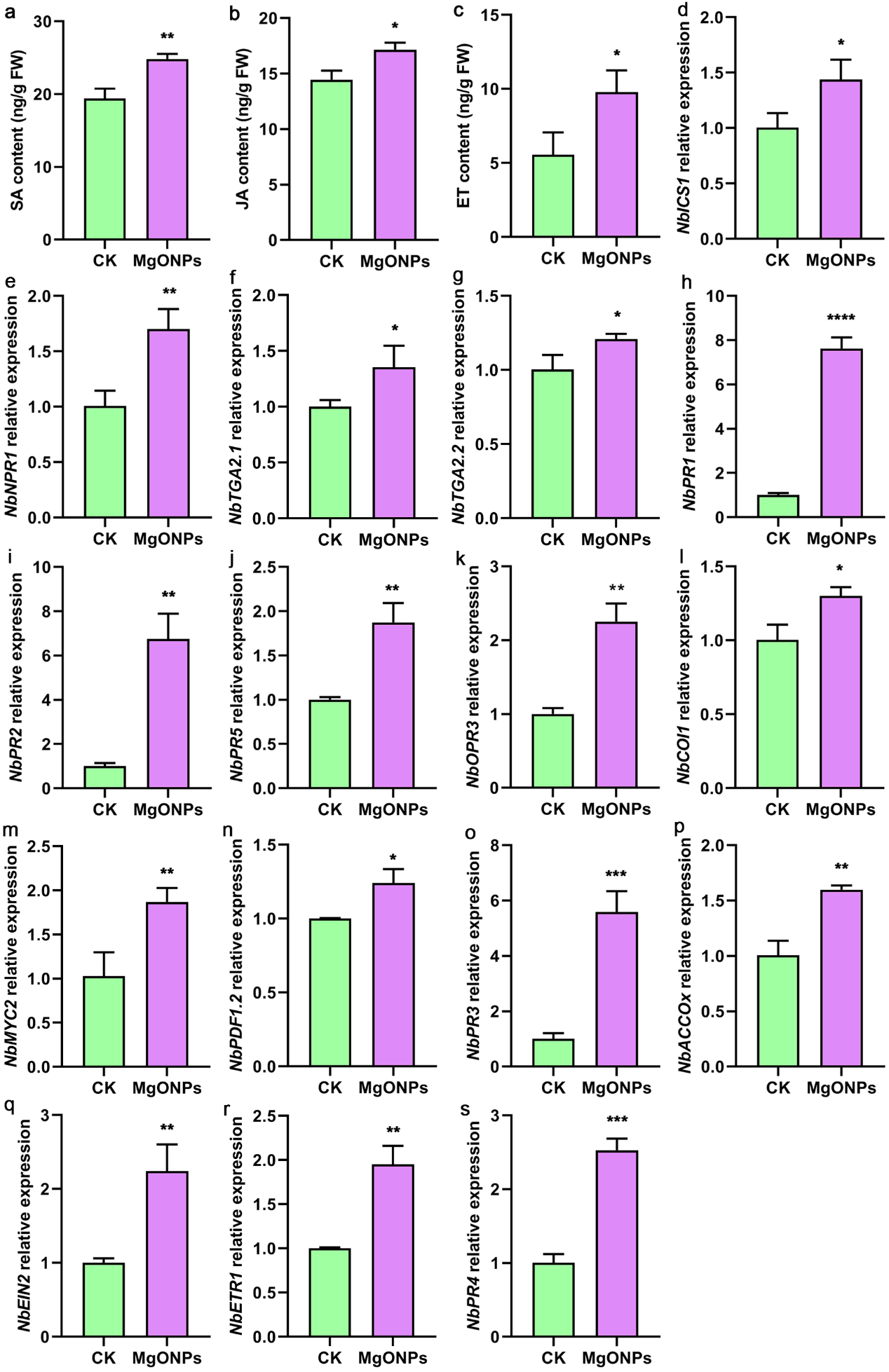


**Figure S16** **MgONPs treatment activates the SA, JA and ET-mediated signaling pathways. a-c,** Analysis of SA (**a**), JA (**b**) and ET (**c**) concentrations in *N. benthamiana* plants treated with MgONPs (150 μg/mL) or ddH_2_O for 3 days. **d-j,** RT-qPCR analysis of the expression of the SA biosynthesis gene *NbICS1* (**d**), the SA-signaling pathway genes *NbNPR1* (**e**), *NbTGA2.1* (**f**), *NbTGA2.2* (**g**), and SA-related defense genes *NbPR1* (**h**), *NbPR2* (**i**), *NbPR5* (**j**) in *N. benthamiana* plants treated with MgONPs (150 μg/mL) or ddH_2_O for 3 days. **k-o,** Relative expression of the JA biosynthesis gene *NbOPR3* (**k**), the JA-signaling pathway genes *NbCOI1* (**l**), *NbMYC2* (**m**), and JA-related defense genes *NbPDF1.2* (**n**), *NbPR3* (**o**) in *N. benthamiana* plants treated with MgONPs or ddH_2_O, as determined by RT-qPCR. **p-s,** RT-qPCR analysis of the expression of the ET biosynthesis gene *NbACCO_X_* (**p**), the ET-signaling pathway genes *NbEIN2* (**q**), *NbETR1* (**r**), and ET-related defense gene *NbPR4* (**s**) in *N. benthamiana* plants treated with MgONPs or ddH_2_O. *ACTIN* was used as the internal reference gene, and the expression is relative to that in the WT with ddH_2_O treatment, the value of which was set as 1. Asterisks represent signiﬁcant differences determined by Student’s t-test (**P* < 0.05; ***P* < 0.01; ****P* < 0.001; *****P* < 0.0001).

**
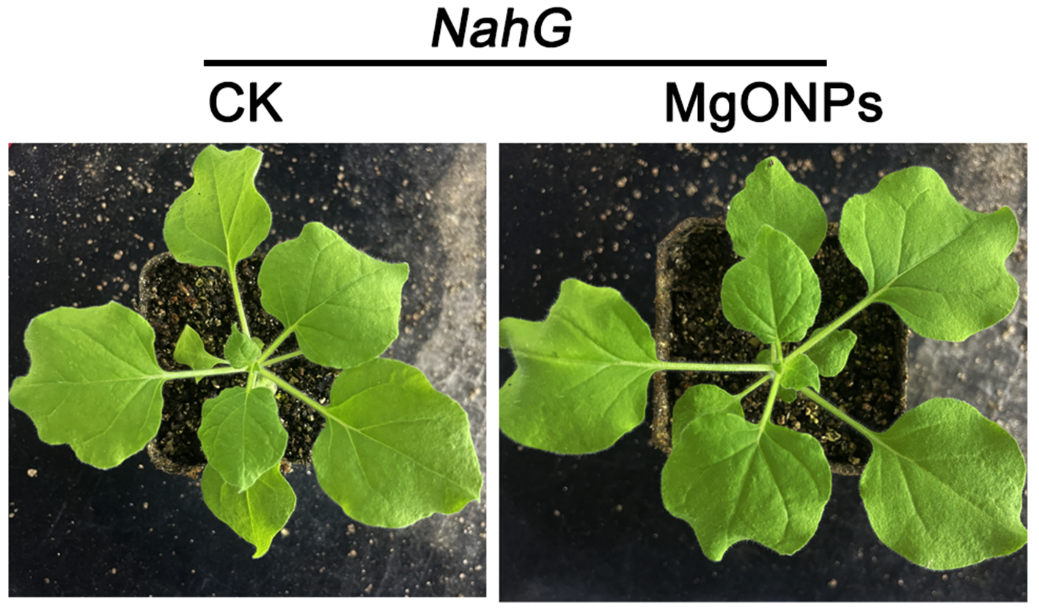
**

**Figure S17** **Phenotype of *NahG-*transgenic plants treated with 150 μg/mL MgONPs or ddH_2_O for 3 days.**

**
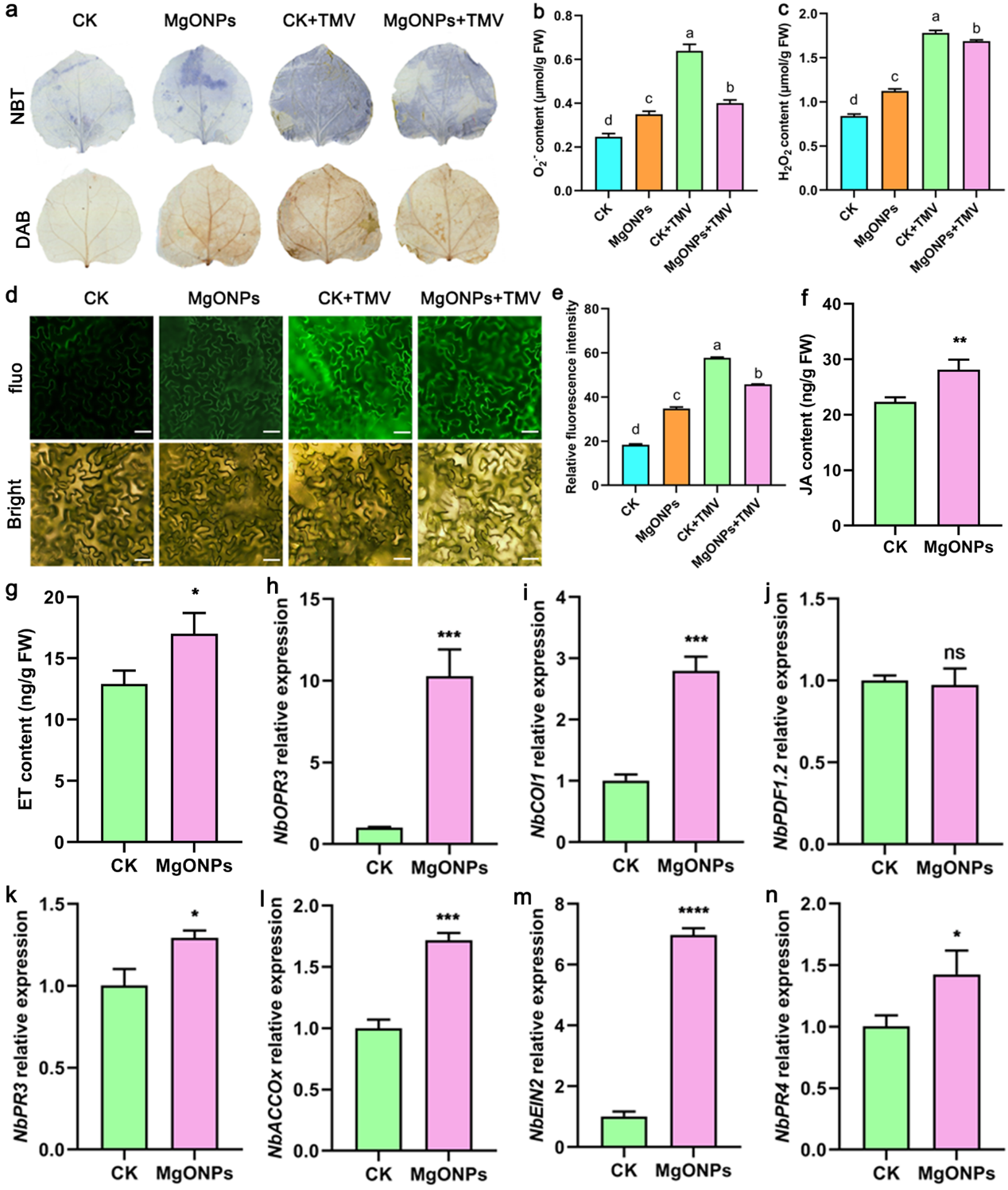
**

**Figure S18** **MgONPs reduce TMV-induced ROS and activate JA- and ET-mediated defense pathways in *NahG-*transgenic plants. a**, The levels of O_2_^•-^ and H_2_O_2_ were visualized by NBT and DAB staining, respectively, in *NahG*-transgenic plants under MgONPs or water treatments at 3 dpi with TMV-GFP. **b,c**, The O_2_^•-^ (**b**) and H_2_O_2_ (**c**) content was measured in leaves of *NahG*-transgenic plants under MgONPs or water treatments at 3 dpi with TMV-GFP by a Micro Superoxide Anion Assay Kit and a Hydrogen Peroxide Assay Kit, respectively. **d**, H_2_O_2_ levels in *NahG*-transgenic plants under MgONPs or water treatments at 3 dpi with TMV-GFP stained with H_2_DCF fluorescent probe were detected by fluorescence microscope. Scale bar, 50 μm. **e**, Relative fluorescence intensity of H_2_O_2_ signals in the leaves shown in **d**. **f,g**, Analysis of JA (**f**) and ET (**g**) concentrations in *NahG*-transgenic plants treated with MgONPs or ddH_2_O. **h-k**, RT-qPCR analysis of the expression of the JA biosynthesis gene *NbOPR3* (**h**), the JA-signaling pathway gene *NbCOI1* (**i**), and JA-related defense genes *NbPDF1.2* (**j**), *NbPR3* (**k**) in *NahG*-transgenic plants under MgONPs or water treatments. **l-n**, Relative expression of the ET biosynthesis gene *NbACCO_X_* (**l**), the ET-signaling pathway gene *NbEIN2* (**m**), and ET-related defense gene *NbPR4* (**n**) in *NahG*-transgenic plants under MgONPs or water treatments, as determined by RT-qPCR. *ACTIN* was used as the internal reference gene, and the expression is relative to that in the *NahG*-transgenic plants with ddH_2_O treatment, the value of which was set as 1. Asterisks represent significant difference determined by Student’s t test between two groups (**P* < 0.05, ** *P* < 0.01, *** *P* < 0.001, **** *P* < 0.0001). Different letters indicate significant differences as determined using one-way ANOVA followed by Tukey’s test between multiple groups (*P* < 0.05).

**
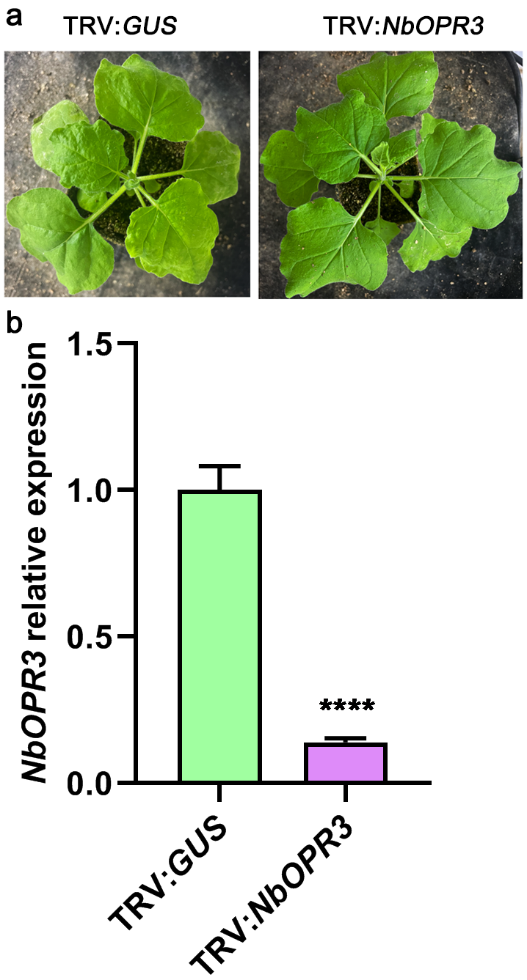
**

**Figure S19.** **Silencing *NbOPR3* in *N. benthamiana* plants by TRV-mediated VIGS.** (a) Phenotype of *NbOPR3*-silenced *N. benthamiana* and control plants (TRV:*GUS*). (b) RT-qPCR analysis of the expression levels of *NbOPR3* in *NbOPR3*-silenced *N. benthamiana* plants. *ACTIN* was used as the internal reference gene, and the expression is relative to that in the control plants (TRV:*GUS*), the value of which was set as 1. Bars represent mean and standard deviation of values obtained from three biological replicates. Asterisks represent significant difference determined by Student’s *t* test (**** *P* < 0.0001).

**
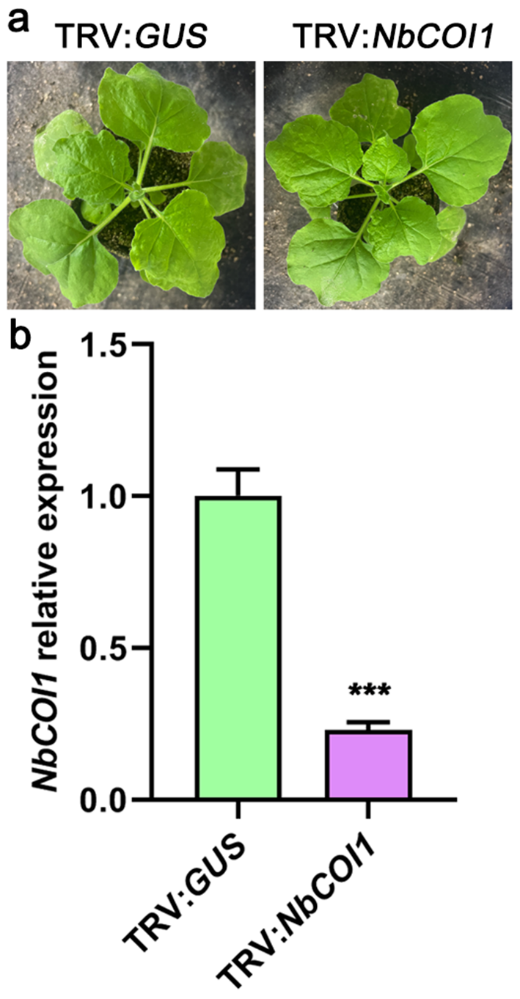
**

**Figure S20** **Silencing *NbCOI1* in *N. benthamiana* plants by TRV-mediated VIGS.** (a) Phenotype of *NbCOI1*-silenced *N. benthamiana* and control plants (TRV:*GUS*). (b) RT-qPCR analysis of the expression levels of *NbCOI1* in *NbCOI1*-silenced *N. benthamiana* plants. *ACTIN* was used as the internal reference gene, and the expression is relative to that in the control plants (TRV:*GUS*), the value of which was set as 1. Bars represent mean and standard deviation of values obtained from three biological replicates. Asterisks represent significant difference determined by Student’s *t* test (*** *P* < 0.001).

**
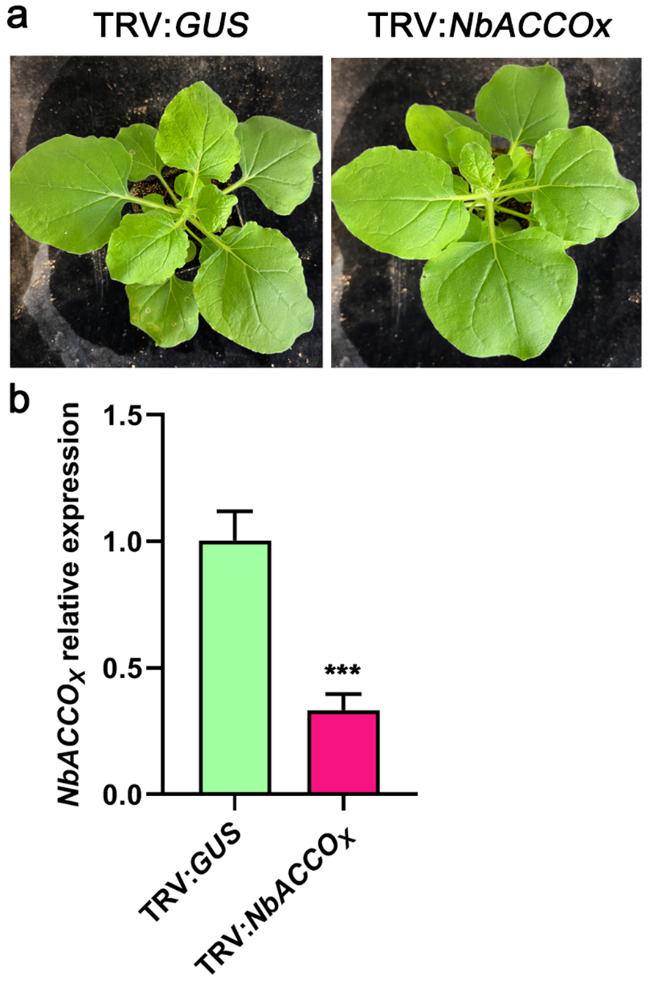
**

**Figure S21** **Silencing *NbACCOx* in *N. benthamiana* plants by TRV-mediated VIGS.** (a) Phenotype of *NbACCOx*-silenced *N. benthamiana* and control plants (TRV:*GUS*). (b) RT-qPCR analysis of the expression levels of *NbACCOx* in *NbACCOx*-silenced *N. benthamiana* plants. *ACTIN* was used as the internal reference gene, and the expression is relative to that in the control plants (TRV:*GUS*), the value of which was set as 1. Bars represent mean and standard deviation of values obtained from three biological replicates. Asterisks represent significant difference determined by Student’s *t* test (*** *P* < 0.001).

**
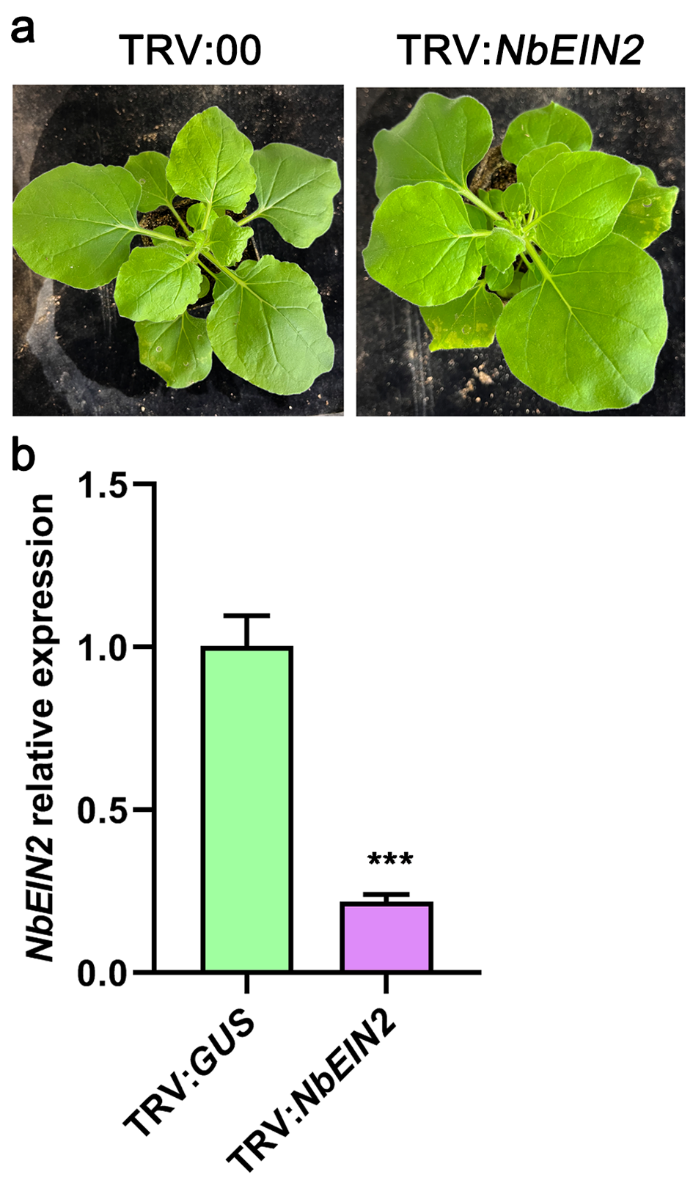
**

**Figure S22** **Silencing *NbEIN2* in *N. benthamiana* plants by TRV-mediated VIGS.** (a) Phenotype of *NbEIN2*-silenced *N. benthamiana* and control plants (TRV:*GUS*). (b) RT-qPCR analysis of the expression levels of *NbEIN2* in *NbEIN2*-silenced *N. benthamiana* plants. *ACTIN* was used as the internal reference gene, and the expression is relative to that in the control plants (TRV:*GUS*), the value of which was set as 1. Bars represent mean and standard deviation of values obtained from three biological replicates. Asterisks represent significant difference determined by Student’s *t* test (*** *P* < 0.001).

**
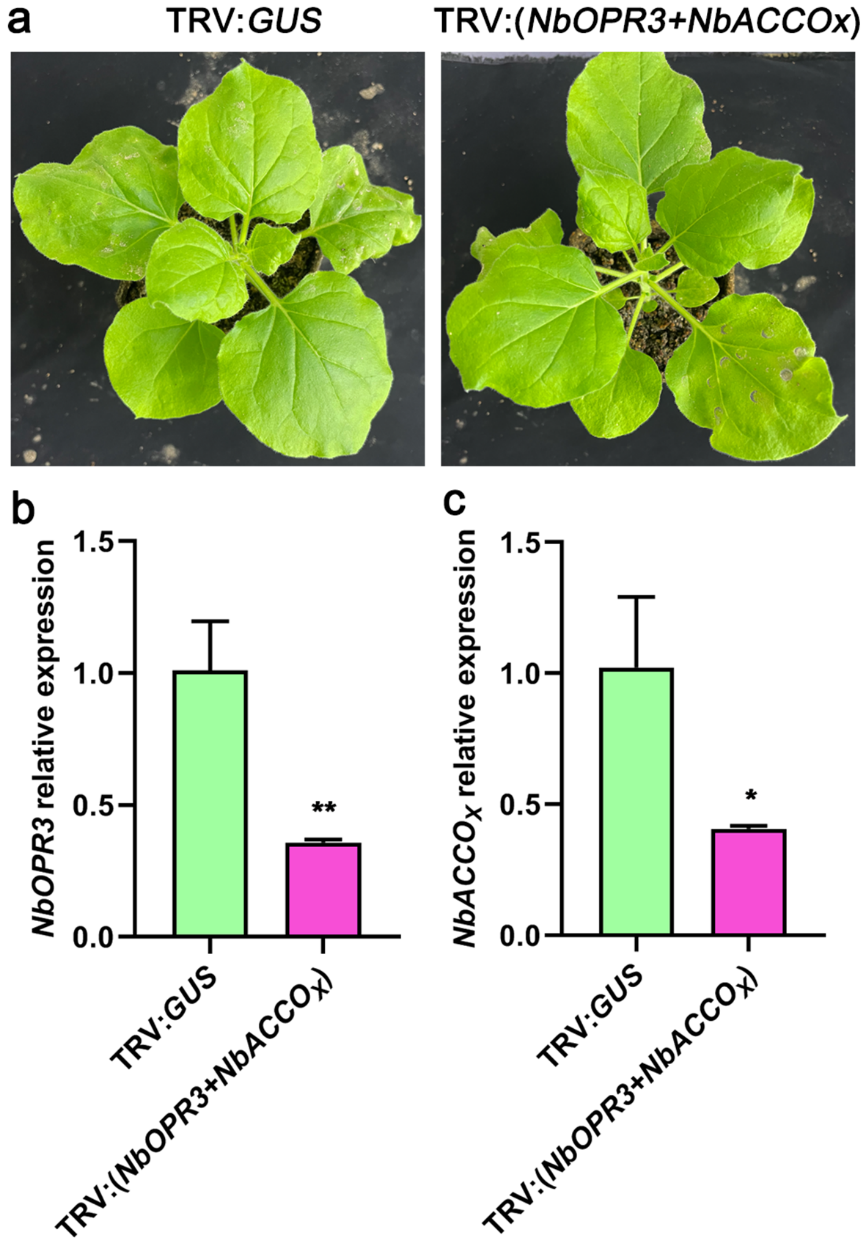
**

**Figure S23** **Simultaneously silencing *NbOPR3* and *NbACCOx* in *NahG*-transgenic plants by TRV-mediated VIGS.** (a) Phenotype of (*NbOPR3+NbACCOx*)-silenced *NahG* plants and control plants (TRV:*GUS*). RT-qPCR analysis of the expression levels of *NbOPR3* (b) and *NbACCOx* (c) in (*NbOPR3+NbACCOx*)-silenced *NahG* plants. *ACTIN* was used as the internal reference gene, and the expression is relative to that in the control plants (TRV:*GUS*), the value of which was set as 1. Bars represent mean and standard deviation of values obtained from three biological replicates. Asterisks represent significant difference determined by Student’s *t* test (* *P* < 0.05, ** *P* < 0.01).

**
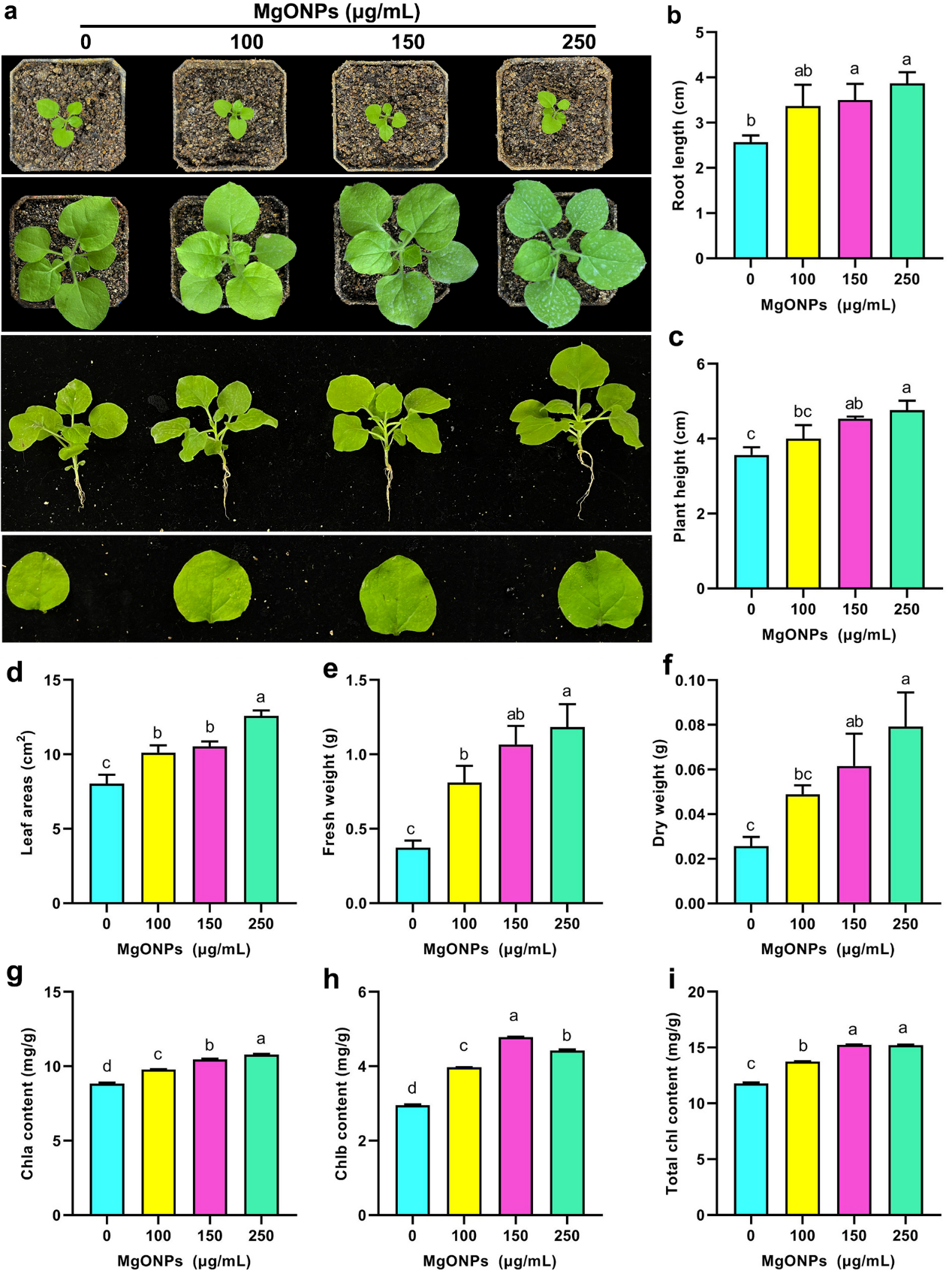
**

**Figure 24** **MgONPs promote plant growth. a**, The phenotype of *N. benthamiana* plants treated with different concentrations of MgONPs. **b-i**, The root length (**b**), plant height (**c**), leaf area (**d**), fresh weight (**e**), dry weight (**f**), chlorophyll *a* content (**g**), chlorophyll *b* content (**h**), and total chlorophyll content (**i**) were analyzed in different concentrations of MgONPs-treated *N. benthamiana* plants. Different letters indicate significant differences as determined using one-way ANOVA followed by Tukey’s test between multiple groups (*P* < 0.05).

**
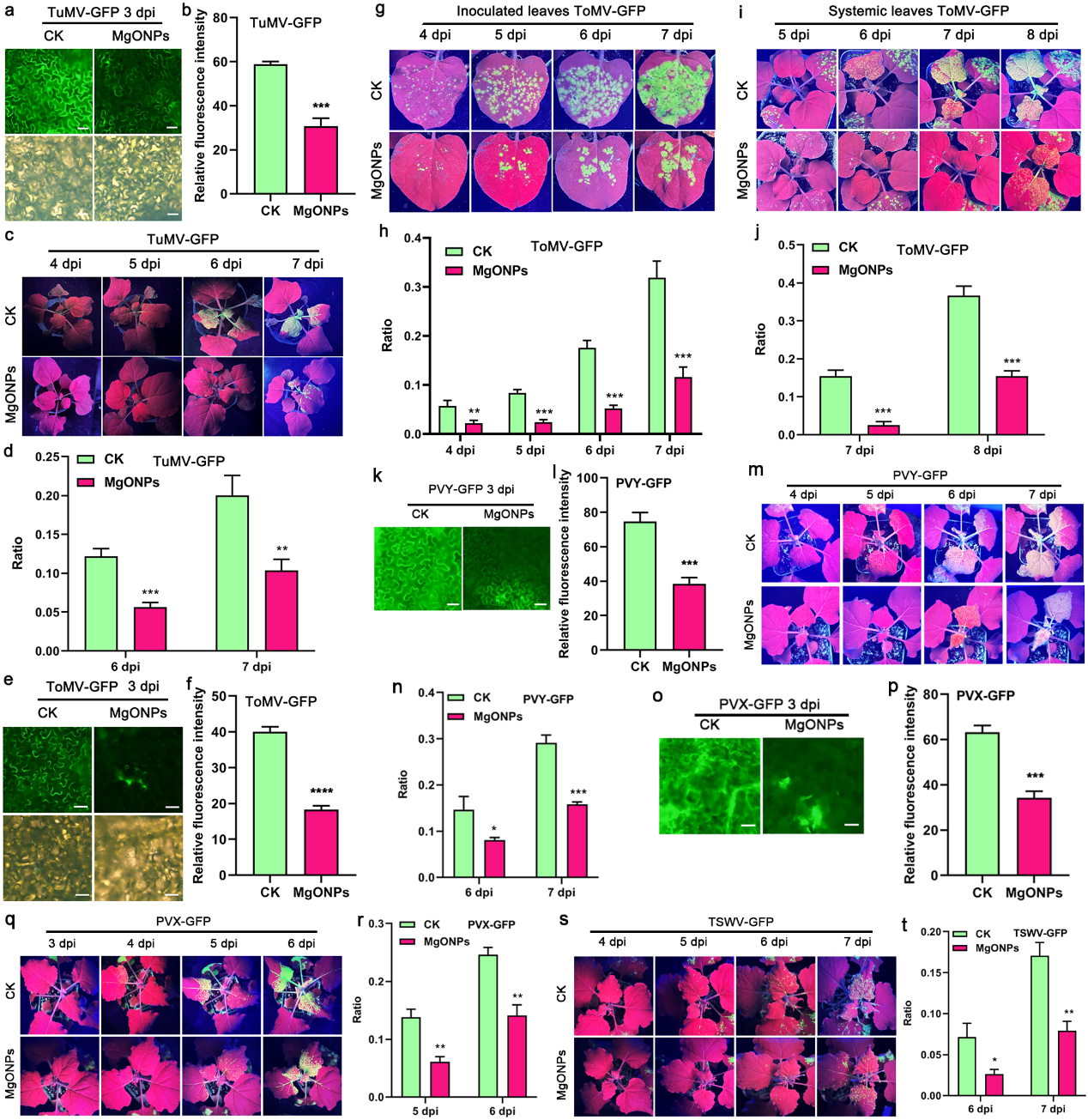
**

**Figure S25** **Foliar application of MgONPs induces broad-spectrum resistance of *N. benthamiana* to major viral diseases of vegetable crops. a-d,** Foliar application of MgONPs enhances resistance to TuMV-GFP in *N. benthamiana*. **a**, Fluorescence images of GFP signaling in the inoculated leaves of MgONPs-treated plants and water-treated plants (CK) at 3 dpi after infection with TuMV-GFP by Fluorescence microscope. Scale bar, 50 μm. **b**, Relative fluorescence intensity of GFP signals of the leaves shown in **a**. **c**, Representative images of GFP fluorescence visualized in the systemic leaves of MgONPs-treated plants and water-treated plants (CK) at different time points after infection with TuMV-GFP*.* **d**, The ratio of GFP fluorescence area to the total area of the systemic leaves of MgONPs-treated plants and water-treated plants (CK) shown in **c** at 6 dpi and 7 dpi. **e-j,** Foliar application of MgONPs increases *N. benthamiana* resistance to ToMV-GFP infection. **e**, Fluorescence images of GFP signaling in the inoculated leaves of MgONPs-treated plants and water-treated plants (CK) at 3 dpi after infection with ToMV-GFP by Fluorescence microscope. Scale bar, 50 μm. **f**, Relative fluorescence intensity of GFP signals of the leaves shown in **e**. **g**, Representative images of GFP fluorescence visualized in the inoculated leaves of MgONPs-treated plants and water-treated plants (CK) at different time points after infection with ToMV-GFP. **h**, The ratio of GFP fluorescence area to the total area of the inoculated leaves of MgONPs-treated plants and water-treated plants (CK) shown in **g** at 4-7 dpi. **i**, Representative images of GFP fluorescence visualized in the systemic leaves of MgONPs-treated plants and water-treated plants (CK) at different time points after infection with ToMV-GFP*.* **j**, The ratio of GFP fluorescence area to the total area of the systemic leaves of MgONPs-treated plants and water-treated plants (CK) shown in **i** at 7 dpi and 8 dpi. **k-n,** Foliar application of MgONPs enhances *N. benthamiana* resistance to PVY-GFP infection. **k**, Fluorescence images of GFP signaling in the inoculated leaves of MgONPs-treated plants and water-treated plants (CK) at 3 dpi after infection with PVY-GFP by Fluorescence microscope. Scale bar, 50 μm. **l**, Relative fluorescence intensity of GFP signals of the leaves shown in **k**. **m**, Representative images of GFP fluorescence visualized in the systemic leaves of MgONPs-treated plants and water-treated plants (CK) at different time points after infection with PVY-GFP*.* **n**, The ratio of GFP fluorescence area to the total area of the systemic leaves of MgONPs-treated plants and water-treated plants (CK) shown in **m** at 6 dpi and 7 dpi . **o-r,** Foliar application of MgONPs enhances resistance to PVX-GFP infection in *N. benthamiana*. **o**, Fluorescence images of GFP signaling in the inoculated leaves of MgONPs-treated plants and water-treated plants (CK) at 3 dpi after infection with PVX-GFP by Fluorescence microscope. Scale bar, 50 μm. **p**, Relative fluorescence intensity of GFP signals of the leaves shown in **o**. **q**, Representative images of GFP fluorescence visualized in the systemic leaves of MgONPs-treated plants and water-treated plants (CK) at different time points after infection with PVX-GFP*.* **r**, The ratio of GFP fluorescence area to the total area of the systemic leaves of MgONPs-treated plants and water-treated plants (CK) shown in **q** at 5 dpi and 6 dpi. **s,t,** Foliar application of MgONPs enhances *N. benthamiana* resistance to TSWV-GFP infection. **s**, Representative images of GFP fluorescence visualized in the systemic leaves of MgONPs-treated plants and water-treated plants (CK) at different time points after infection with TSWV-GFP*.* **t**, The ratio of GFP fluorescence area to the total area of the systemic leaves of MgONPs-treated plants and water-treated plants (CK) shown in **s** at 6 dpi and 7 dpi. Asterisks represent significant difference determined by Student’s t test (**P* < 0.05, ** *P* < 0.01, *** *P* < 0.001, **** *P* < 0.0001).

**
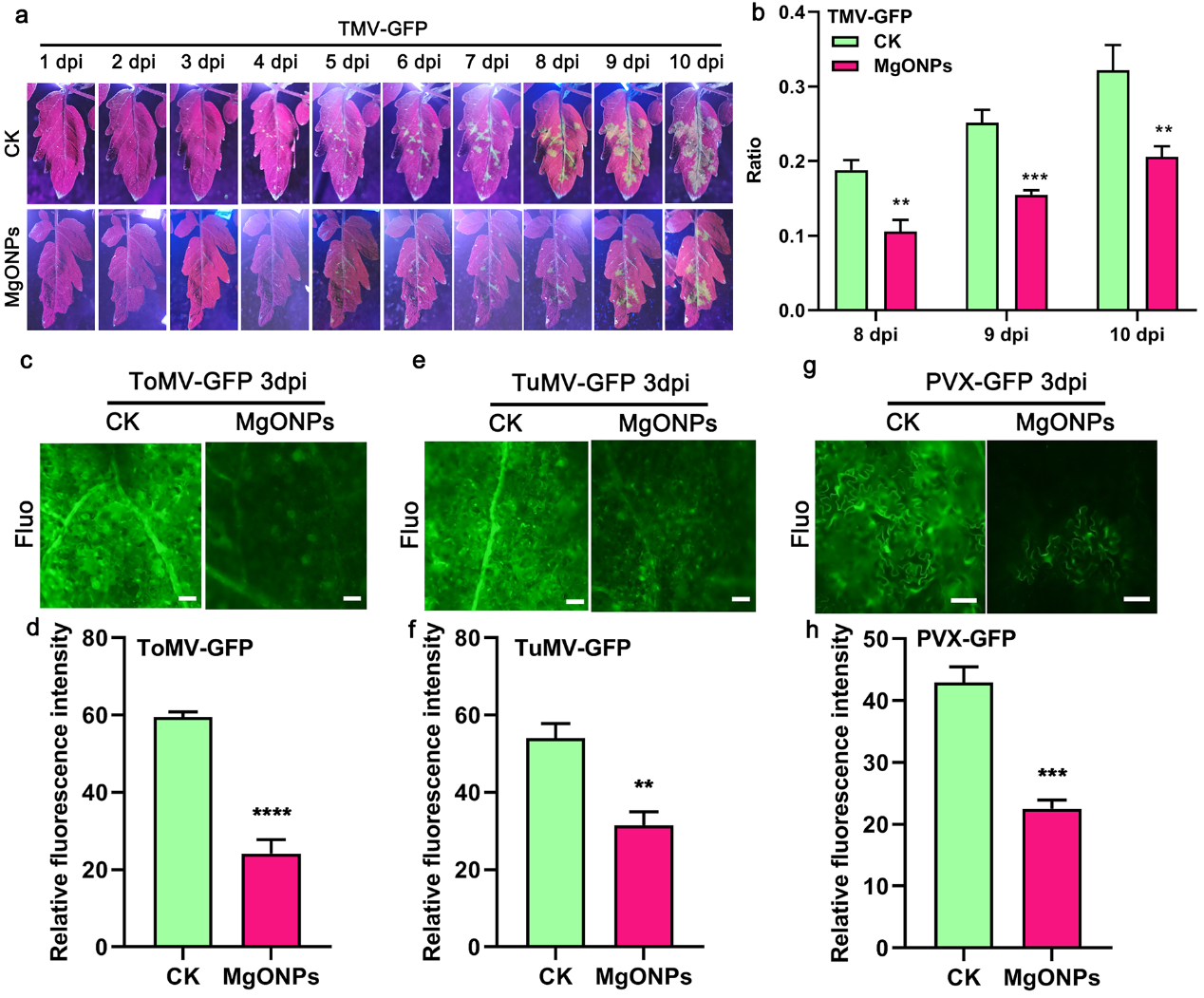
**

**Figure S26** **MgONPs induce broad-spectrum resistance of *S. lycopersicum* to major viral diseases of vegetable crops. a-b**, Foliar application of MgONPs enhances resistance to TMV-GFP in *S. lycopersicum*. (**a**) Representative images of GFP fluorescence visualized in the inoculated leaves of MgONPs-treated plants and water-treated plants (CK) at different time points after infection with TMV-GFP. (**b**) The ratio of GFP fluorescence area to the total area of the systemic leaves of MgONPs-treated plants and water-treated plants (CK) shown in **a** at 8-10 dpi. **c-d**, Foliar application of MgONPs enhances *S. lycopersicum* resistance to ToMV-GFP infection. (**c**) Fluorescence images of GFP signaling in the inoculated leaves of MgONPs-treated plants and water-treated plants (CK) at 3 dpi after infection with ToMV-GFP by Fluorescence microscope. Scale bar, 50 μm. (**d**) Relative fluorescence intensity of GFP signals of the leaves shown in **c**. **e-f**, Foliar application of MgONPs increases resistance to TuMV-GFP in *S. lycopersicum*. (**e**) Fluorescence images of GFP signaling in the inoculated leaves of MgONPs-treated plants and water-treated plants (CK) at 3 dpi after infection with TuMV-GFP by Fluorescence microscope. Scale bar, 50 μm. (**f**) Relative fluorescence intensity of GFP signals of the leaves shown in **e**. **g-h**, Foliar application of MgONPs enhances *S. lycopersicum* resistance to PVX-GFP infection. (**g**) Fluorescence images of GFP signaling in the inoculated leaves of MgONPs-treated plants and water-treated plants (CK) at 3 dpi after infection with PVX-GFP by Fluorescence microscope. Scale bar, 50 μm. (**h**) Relative fluorescence intensity of GFP signals of the leaves shown in **g**. Asterisks represent significant difference determined by Student’s t test (** *P* < 0.01, *** *P* < 0.001, **** *P* < 0.0001).

**
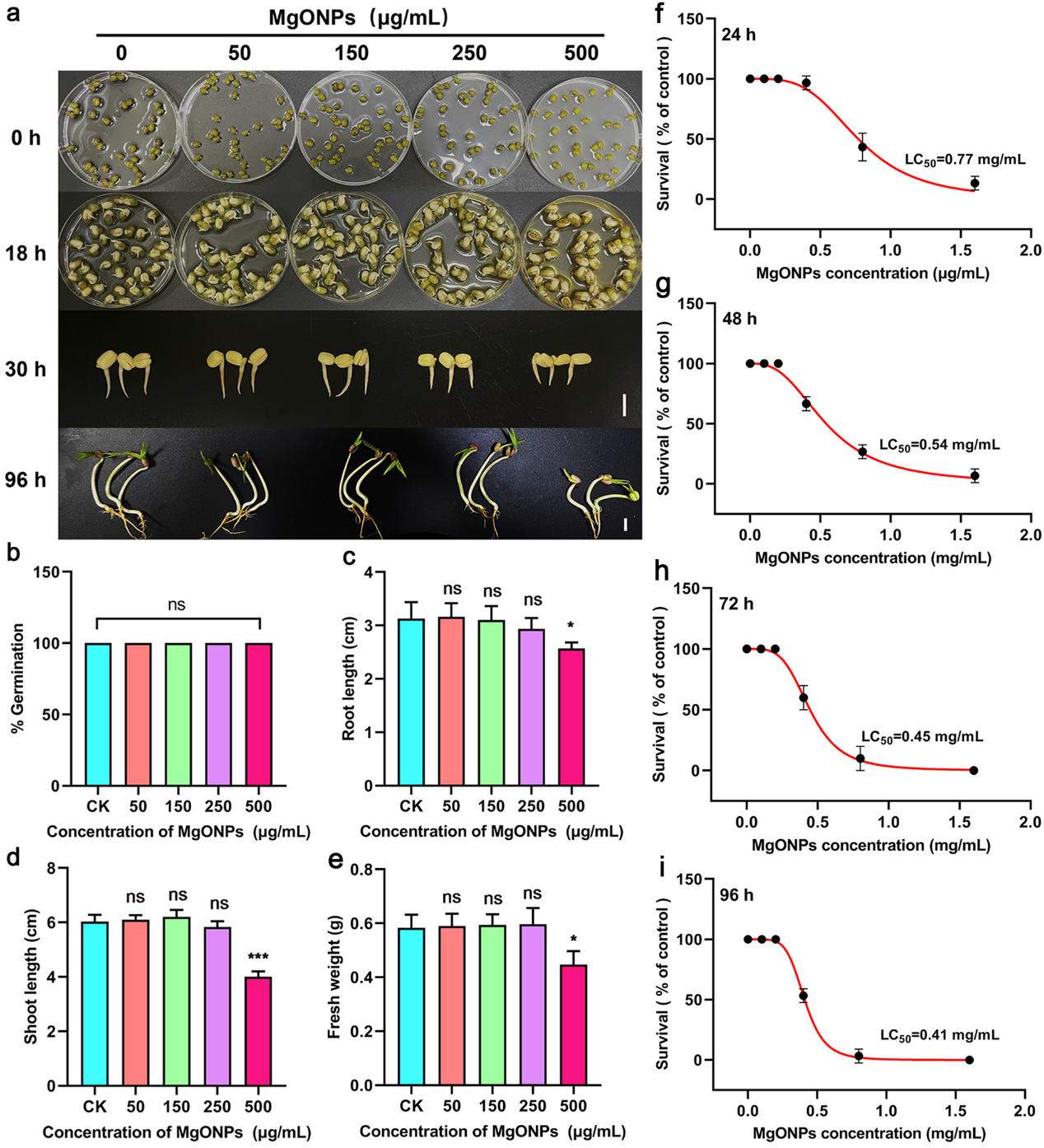
**

**Figure S27** **Safety evaluation of MgONPs in mung bean plants and zebrafish. a-e,** Phytotoxicity analysis of MgONPs with different concentrations to mung bean plants. **a**, Representative images of mung bean seeds treated with different concentrations of MgONPs (0–500 μg/mL) at different time points (0, 18, 30 and 96 h). Scale bar, 1 cm. **b**, Percent of germination of mung bean seeds treated with MgONPs at different concentrations (0–500 μg/mL) (NS: Not significant). **c**, Root length analysis on the 4th-day of MgONPs exposure at different concentrations. **d**, Shoot length analysis on the 4th-day of MgONPs exposure at different concentrations. **e**, Fresh weight analysis on the 4th-day of MgONPs exposure at different concentrations. **f-i,** Dose-dependent effect of MgONPs on survival in zebrafish after 24 h (**f**), 48 h (**g**),72 h (**h**), 96 h (**i**) of incubation. Asterisks represent significant difference determined by Student’s t test (**P* < 0.05, *** *P* < 0.001).

**Table S1 Primer sequences used for quantitative real-time PCR analysis of gene expression**

| **Gene** | **F primer (5’→3’)** | **R primer (5’→3’)** |
| --- | --- | --- |
| *NbActin* | AACTGATGAAGATACTCACA | CAGGATACGGGGAGCTAAT |
| *TMV-MP* | GACCTGACAAAAATGGAGAAGATCT | GAAAGCGGACAGAAACCCGCTG |
| *NbGLR1.1* | AGGGGAATTGAACCGTGTCC | GGTTCTCCCTGGAACACCAA |
| *NbGLR2.1* | AGAGCAGCACTTCCTTCTTCC | GCATCGGTCAAGTGTGGAAC |
| *NbGLR2.2* | CGGGTGTAGGCAATGAAAGC | GGCTTTGCGAGGGAAAGTTG |
| *NbGLR2.3*  *NbGLR2.4* | TCCGGGCGAAGTCAATGAAA GGACTGAAAGAGCCACCCAA | GCTTTGTGAGGGAAGGTTGG GGCCGACTTCTGATTTCCCA |
| *NbGLR2.5* | CACAGAATCTGGTGTCACAATG | CCACCATTCGCACTACC |
| *NbGLR3.1* | GGGCCTCGTGTTTTTGTTGT | CACACGTAATCGCTCCCAGT |
| *NbGLR3.2* | CAGGAGTTCACTAAGGGCGG | GCATCCGTTGTTCGTTAGCC |
| *NbGLR3.3* | TGCCGTATGCTGTTCCACAT | GGTCTTAGGAAGGCCCAACC |
| *NbGLR3.4* | GAGCTTCTGGGGCCTGTATG | GGTTCACTGATCTCCGGCTC |
| *NbGLR3.5* | CTGCCACCATTATCCCGAGT | GTCCAGTTGCCTTCGTTTGG |
| *NbGLR3.6* | TTGTGGACTTCACGCAACCT | CCCCAGTTACGGACCACATT |
| *NbCaM1* | ACCCCAAAAATACTGGCACA | TCTGAGATTTTTCTTTGTTGAAAGG |
| *NbCaM2* | AGAGGCGTTCAGAGTGTTCG | TTTGGCCATCACCATCGACA |
| *NbCaM3* | GATCACGTCCGCATACATTG | TCGGAACTATGGGATTATTGC |
| *NbCaM4* | TTGGAACTGTGATGCGGTCA | GGTCGCTGGGACAAAATCCA |
| *NbCaM5* | GGTCGCTGGGACAAAATCCA | TCATCTTGCGAGCCATCAGG |
| *NbCaM6* | TGAGGAGAAACGAAGGGTAAA | TCTCACCTCTTGGATTTCAGG |
| *NbCBP60g* | TCCTTTCCATGCCCGTTTGA | ACCCCTGCCAAGGATACTGA |
| *NbCDPK2* | ACTGCAGACGAGCTTCAACA | ACCGGATTCCCCTTTTGCAT |
| *NbCDPK4* | GCCTACAATTTCCCATGGTGC | GCAATTACCCGGAGAGCAAC |
| *NbCDPK5* | CCTGCAATTTCCCATGGTGC | GGGCAAGTCCTTGTTTTAGCTC |
| *NbCDPK6* | GGGAGGCATTATCCGACGAG | TACCGGCCTTCATCATCACG |
| *NbGɑ* | AGTGCCTCTGAACGTCTGTG | TTAAAGACCCGGTCCACACG |
| *NbRbohA* | ACATTCGAACAGTGGGCGAT | GCTTATAGTCGGCCTTCGCT |
| *NbRbohB* | CAAACGAGGCGGCAAAAAGA | GGACAGCGACGGAATCTTCA |
| *NbRbohC* | GGGCTGGATTCTGCTGCTAT | GCTGTTGCCAAGCAGTAACC |
| *NbRbohD* | CCTGGATCGTTCGAGTGGTT | TAAGTGCCTGGAGCATGGTG |
| *NbRbohE* | CGCTCTTTCATACGAGCTGC | TGCCAGGGATAGATGCAACG |
| *NbRbohF* | ATGTGAAATTGGGCGGGCTA | TTGATAGTCCTGTGCTGCGG |
| *NbRbohG* | GTCTTGGCATTGGAGCAACG | TGCAACGCTCATGTCTGAGT |
| *NbRbohH* | TCCACATGATGCCGACCAAT | ACGGTGTTGCTCCAATTCCT |
| *NbCAT1* | AGCCAAATCCTAAGTCCC | CCAGCAGCAATAGAGTCAT |
| *NbAPX1* | GTGCTCCTATTATGCTCCGTCTT | GGTGGCTCTGGCTTGTCCT |
| *NbAPX3* | TACTTCGGTTTCAGATGGC | TCAGCGTAGGATAGGATTGG |
| *NbAPX4* | GGTTTACCCCAAGTTCTG | CTTCATCCTTTTCCGCAC |
| *NbAPX5* | GAGAGCATTTAGGAGAGG | TCAGCAGACAAGGACCAG |
| *NbAPX7* | CAGTTGAAAAGTGCGAGA | AATGAGCAGCAGGGGAAG |
| *NbFeSOD* | CCTATGGATGCTTTGGGG | TGGTGAGGAGCGGTGTGT |
| *NbCu/ZnSOD* | ATTTTTGGTGTTGTTCGC | AGTTTGTTGGTTGCTTCC |
| *NbICS1* | CGTGCTTATGGGGCAATTCG | ATTGCCATCTGGTACGTGCA |
| *NbNPR1* | ACATCAGCGGAAGCAGTAG | GTCGGCGAAGTAGTCAAAC |
| *NbPR1* | ATGGTCAATACGGCGAAAAC | CCTAGCACATCCAACACGAA |
| *NbPR2* | CTAATGGCATCAGAAAGA | ATTGGCTAAGAGTGGAAG |
| *NbPR5* | AGCCGTATCAGGAATGCTGC | CGAGCTTGTTCTGGCTCTCA |
| *NbCOI1* | GCCACTTGATAATGGTGT | AGGCCTTCATCGGATTCC |
| *NbOPR3* | ATGGTGGATCGCTAGGCAAC | TAGGCAACGTATCGTGGCTG |
| *NbPR3* | GTACCCCAAGTGGTCAGTGG | CTTCCACAAGGCCCGTAGTT |
| *NbPDF1.2* | CCAACGACAATTGCAGAGGC | CCTTCGGTCAAACAGACGGT |
| *NbEIN2* | ACCATCTCCTCGTTCT | ATTATTTCGGGCTACA |
| *NbACCOx* | TATGGTTCAAAGGGTC | TGGTGCTGGATAGATG |
| *NbPR4* | TAGATGCTTGAGGGTG | TTGACAGTAAGGTGGC |
| *NbTGA2.1* | CAACTAATCCAGCCGTGGGT | AGTGCCTGATGCTGAAACCA |
| *NbTGA2.2* | AATGGGGAAGCTCGGAACTC | AGCTGATTGGCGAGTTGTCA |
| *NbMYC2* | CCTGCAGCTGTACCTGTGAA | GCTGAGAATGGTGCTGTTGC |
| *NbETR1* | AAGGCTTGAGGATGGAAGCC | ATTCCGGCAGATCCGAAGAC |

**Table S2 Primer sequences used for semiquantitative RT-PCR analysis of vector construction for VIGS.**

| **Gene** | **F primer (5’→3’)** | **R primer (5’→3’)** |
| --- | --- | --- |
| *NbGLR3.3-VIGS* | TGATGAACCTATTGGG | ATGTATTCGCTGGAGA |
| *NbRbohA-VIGS* | GCCGGTGGACATCCGATACG | TCGTCGGCGATCGAGATTCCG |
| *NbRbohB-VIGS* | AGAGCGTCGTACAGTGGTC | GTAGGAATTGGGCGTTTAT |
| *NbACCOx-VIGS* | GGTTCAAATGGTCCTACT | ATTCTGGTGCTGGATAGA |
| *NbEIN2-VIGS* | ACCATTTGCTCGTGCTTTG | TGCCCTCAGTGAACCAGTC |
| *NbOPR3-VIGS* | GCCAAACAGAGGCAGGTA | TCACAGACGCGACAAGGA |
| *NbCOI1-VIGS* | TAATGGTGTCCGTGCTT | TGCCCTGTATCCTTGTA |

**Table S3 Primer sequences used for semiquantitative RT-PCR analysis of *NbGLR3.3* vector construction for CRISPR and knockout validation.**

| **Gene** | **F primer (5’→3’)** | **R primer (5’→3’)** |
| --- | --- | --- |
| *NbGLR3.3-gRNA* | CGATTCCCGGCTGGTGCAGTAGCAGCAAATGATAAGAAGTTTTAGAGCTAGAAATA | TTCTAGCTCTAAAACACCATAAT ATTCAACGATCTTGCACCAGCC GGGAAT |
| *NptII* | ACTGGGCACAACAGACAATCG | GCATCAGCCATGATGGATACTTT |
| *Target* | CAGAACATCCATAACATCACCTC | TGGAGACTGATGTTGTTGCAG |
